# Single-cell analysis of heterogeneity in reverted hiPSC-derived human hepatic stellate cells

**DOI:** 10.1016/j.jhepr.2025.101669

**Published:** 2025-11-10

**Authors:** Xinjia Wang, Eun Hee Ha, Lu Bian, Zhuoying Feng, Fan Zhang, Kyle O’Shaughnessy, Lei Wang, Andrea Hochwald, Yifei Zheng, Weibo Chen, Yujie Zhang, Xianfang Wu

**Affiliations:** 1 Department of Infection Biology, Lerner Research Institute, Cleveland Clinic, Cleveland, OH, USA; 2 Department of Genetics and Genome Sciences, School of Medicine, Case Western Reserve University, Cleveland, OH, USA; 3 Department of Pathology School of Medicine, Case Western Reserve University and University Hospitals Cleveland Medical Center, Cleveland, OH, USA; 4 Cleveland Clinic College of Medicine at Case Western Reserve University, Cleveland, OH, USA

**Keywords:** Hepatic stellate cells (HSC), HSC reversion, Single-cell RNA sequencing, Heterogeneity, Re-stimulation, HSC-macrophage interaction, IL-10 signaling, Multicellular liver culture, Disease modeling

## Abstract

**Background & Aims:**

Activated HSCs are known to drive fibrogenesis, but their fate following injury resolution remains unclear. We aimed to investigate whether human activated HSCs revert to a less activated state, and to characterize features of such reversion using a human induced pluripotent stem cell (hiPSC)-derived multicellular liver model.

**Methods:**

We used a hiPSC-derived liver culture containing hepatocytes, HSCs, and macrophages. HSCs were activated by HCV infection or a lipotoxic milieu modeling metabolic dysfunction-associated steatotic liver disease (MASLD) and subjected to injury resolution through antiviral treatment or replacement with a healthy medium. Reverted HSCs were characterized via gene expression profiling, functional assays, and single-cell RNA sequencing (scRNA-seq). The role of macrophage-derived IL-10 in HSC reversion was investigated through receptor knockdown and cytokine treatment experiments.

**Results:**

Following either HCV clearance or withdrawal of lipotoxic stress, activated HSCs reverted to a less activated state, regaining lipid droplets and vitamin A storage while re-expressing quiescent HSC markers. scRNA-seq revealed heterogeneity among reverted HSCs, identifying subpopulations expressing apoptotic, senescent, or quiescent-like signatures. A distinct lipid-high, PTK2-low population closely resembled naïve quiescent HSCs. Functional assays demonstrated that rHSCs retained partial quiescence but exhibited heightened sensitivity to fibrogenic re-stimulation (n = 4, *p* <0.05). Mechanistically, macrophage-derived IL-10 promoted HSC reversion by inducing vitamin A metabolism-related genes, including *LRAT* and *RBP1* (n = 4, *p* <0.01).

**Conclusions:**

Activated human HSCs demonstrate plasticity, reverting to a quiescent-like state following resolution of viral or metabolic injury, although they remain primed for reactivation. Macrophage-derived IL-10 plays a critical role in driving this reversion by regulating vitamin A metabolism. These findings provide insights into HSC dynamics and suggest potential therapeutic avenues for liver fibrosis by targeting HSC reversion.

**Impact and implications:**

Removing the cause of liver injury—curing hepatitis C or withdrawing lipotoxic stress—allows scar-forming liver cells (hepatic stellate cells) to partly revert to a healthier, vitamin-A-storing state; single-cell profiling reveals its heterogeneity and identify a subset nearing true quiescence. This rebound depends on intercellular interaction, in part on the immune signal IL-10 from macrophages, yet reverted cells remain easier to re-activate. These findings provide insights into dynamics of hepatic stellate cells and suggest potential therapeutic avenues for liver fibrosis by targeting stellate cell reversion.

## Highlights

• HSCs revert toward quiescence after HCV cure or lipotoxic withdrawal.
• Reverted HSCs regain vitamin A and quiescent markers but remain primed.
• ScRNA-seq reveals heterogeneity and a lipid-high, PTK2-low rHSC subset.
• Macrophage IL-10 drives reversion via vitamin A metabolism genes (*LRAT* and *RBP1*).

## Graphical abstract

**Figure:**
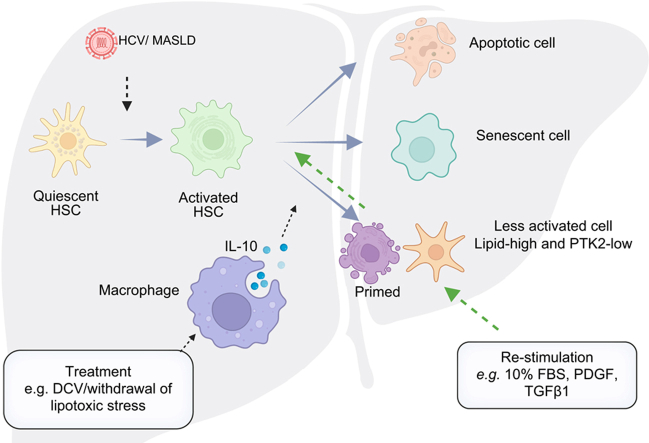

## Introduction

Liver fibrosis, a hallmark of chronic liver disease, arises from the excessive accumulation of extracellular matrix (ECM) components, driven primarily by the activation of hepatic stellate cells (HSCs).^1^ In response to persistent injury—such as viral infection, alcohol exposure, or metabolic stress—HSCs transition from a quiescent, vitamin A-storing state to a myofibroblast-like phenotype characterized by enhanced proliferation, contractility, and production of fibrogenic proteins such as collagen type I. This activation process is well established as the central driver of fibrosis progression, ultimately leading to cirrhosis if unchecked. However, the fate of activated HSCs (aHSCs) following the resolution of the underlying injury remains poorly defined,^1^ particularly in human systems, limiting the development of therapies aimed at reversing fibrosis.

Evidence from rodent models and clinical observations suggests that liver fibrosis can regress when the causative insult is removed,[2], [3], [4] with activated HSCs undergoing apoptosis,^5^^,^^6^ senescence,^1^^,^^7^ or reversion to a less activated, quiescent-like state.^8^^,^^9^ In mice, reverted HSCs regain some quiescent features, such as lipid-droplet storage, but retain a ‘primed’ phenotype, distinct from naïve quiescent HSCs, indicating incomplete restoration.^8^^,^^9^ Although these findings highlight the plasticity of HSCs, their relevance to human liver fibrosis remains uncertain because of the species-specific differences and the lack of robust human-relevant models. Understanding whether human HSCs can revert to a functional, quiescent-like state—and the mechanisms governing this process—is critical for harnessing this plasticity to mitigate fibrosis.

To address this gap, we utilized a human induced pluripotent stem cell (hiPSC)-derived multicellular liver culture system, previously developed by our laboratory, which integrates hiPSC-derived hepatocytes, HSCs, and macrophages to mimic the human liver microenvironment.^10^ This platform enables the study of HSC dynamics in a physiologically relevant context. In this study, we investigated the fate of activated human HSCs following HCV infection and metabolic stress and its subsequent ‘cure’, characterizing their reversion potential and sensitivity to re-stimulation with both familiar and novel fibrogenic stimuli. Additionally, we explored the role of co-cultured macrophages in modulating HSC reversion, focusing on key molecular mediators. Our findings reveal that human HSCs exhibit significant reversion capacity, mediated in part by macrophage-derived IL-10, yet remain functionally distinct from naïve quiescent HSCs (qHSCs), offering new insights into HSC plasticity and potential therapeutic targets for fibrosis resolution.

## Materials and methods

### Single-cell RNA sequencing

Single-cell RNA sequencing (scRNA-seq) was performed at the Genomic Core of Rockefeller University using the 10 × Genomics Chromium platform following the manufacturer’s protocol. Briefly, single-cell suspensions were prepared and adjusted to the desired concentration to achieve optimal cell capture efficiency (∼10,000 cells per sample). Sequencing was performed on an Illumina NovaSeq 6000 platform using paired-end sequencing to achieve a depth of approximately 50,000 reads per cell.

The returned Fastq files of DMSO and DCV samples (DMSO-1, DMSO-2, DCV-1, and DCV-2) were analyzed with Cell Ranger and converted to Seurat objects by running the corresponding scRNA-seq codes provided by the Cleveland Clinic Center for Immunotherapy and Precision Immuno-Oncology (CITI) on a High Performance Computing (HPC) platform. The final integrated Seurat object generated from the four Fastq files was uploaded to BioTuring. All subsequent analyses and graphs (*e.g.* heatmap, differential gene analysis, gene ontology (GO) enrichment analysis, Umap, t-SNE, and violin plots) were generated with the BBrowserX software (BioTuring Inc., San Diego, CA, USA).

### Statistical analysis

Detailed information regarding all statistical tests, the specific value of ‘n’ and its representation (*e.g.* number of cell clones or experimental replicates) can be found in the respective figure legends. Graphs depict results presented as means ± SD. Group comparisons were conducted using the unpaired *t* test with Welch’s correction or one-way ANOVA/Tukey's *post-hoc* test, as appropriate, to calculate precise *p* values unless otherwise specified. Statistical analyses were carried out using Graph Pad PRISM 10 (GraphPad Software, San Diego, CA, USA). All *p* values are reported, and a *p* >0.05 denotes statistical non-significance and is marked as gray.

## Results

### Establishment of a human-relevant *in vitro* system to study HSC activation and reversion

In our recent study modeling fatty liver disease, we developed a multicellular *in vitro* culture system by co-culturing hiPSC-derived hepatocytes, HSCs, and macrophages at an 8:1:1 ratio, closely reflecting the cellular composition of a healthy liver.^10^ We termed this system the ‘multicellular liver culture’. In this system, the co-cultured HSCs remain in a quiescent-like state, characterized by low expression levels of activation markers, while maintaining high levels of quiescence-related markers. Importantly, in response to various stimuli, these HSCs become activated and transdifferentiate into a myofibroblast-like phenotype, exhibiting high expression of activation markers. These features make the multicellular liver culture an ideal platform for investigating HSC activation and potentially the reversion of aHSCs.

To test this possibility, we infected liver cultures with HCV (Fig. S1A), a virus well known for its ability to induce stellate cell activation in patients with this infection.^11^ We first characterized hepatocyte responses: progressively-increasing interferon-stimulated genes, lipid-droplet accumulation with upregulation of lipogenic enzymes, elevated triglyceride and cholesterol, and increased lipid-metabolism genes. Reactive oxygen species (ROS) and malondialdehyde (MDA) were also elevated, indicating oxidative stress and lipid peroxidation (Fig. S1A–K). In parallel, HSCs from HCV-infected cultures showed higher activation markers than HSCs from uninfected cultures and displayed increased cytokine transcripts (Fig. S1L–N). To define the role of macrophages, we compared cultures with and without them: HSC activation was markedly reduced without macrophages. In infected cultures, macrophages shifted from an M2-like (anti-inflammatory) to an M1-like (pro-inflammatory) phenotype, secreting interferon-β, transforming growth factor beta 1 (TGF-β1), and IL-1β, and upregulating *TNF* and *PDGFB* (Fig. S2A–F). Because neither HSCs nor macrophages are permissive to HCV,^12^ these data indicate that infection-induced responses in hepatocytes—and the secondary inflammatory program in macrophages—drive HSC activation.

**Fig. 1:**
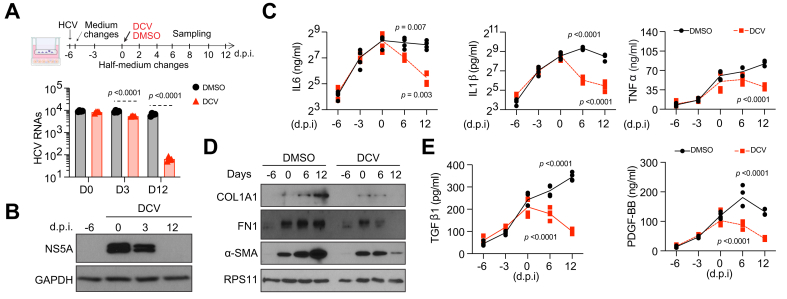
Establishment of a human-relevant *in vitro* system to study HSC activation and reversion. Top: Schematic representation of HCV infection, DCV treatment, and sample collection. Bottom: HCV RNA quantification by qRT-PCR (n = 4, mean ± SD, ANOVA). (B) Western blot analysis of HCV nonstructural protein 5A (NS5A) and housekeeping protein GAPDH in purified hepatocytes. (C, E) ELISA analysis of selected cytokines in cell culture supernatants (n = 4, mean ± SD, ANOVA). (D) Western blot analysis of activation markers and housekeeping protein RPS11 in purified HSCs. α-SMA, alpha-smooth muscle actin; COL1A1, collagen type I alpha 1 chain; DCV, daclatasvir; FN1, fibronectin; GAPDH, glyceraldehyde 3-phosphate dehydrogenase; HSCs, hepatic stellate cells; PDGFB, platelet derived growth factor subunit B; qRT-PCR, quantitative real time-PCR; TGFβ1, transforming growth factor beta 1; TNFα, tumor necrosis factor-alpha; ALDH1A1, aldehyde Dehydrogenase 1A1; LRAT, lecithin-retinol acyltransferase; CYP26A1, cytochrome P450 26A1; PNPLA3, patatin-like phospholipase domain-containing protein 3; HGF, hepatocyte growth factor.

We next examined whether HCV cure reverses HSC activation. Liver cultures were infected with HCV for 1 week before being treated with either daclatasvir (DCV), an HCV nonstructural protein 5A (NS5A) inhibitor, or vehicle (Fig. 1A). As expected, DCV effectively cleared HCV infection (Fig. 1A and B), which by Day 12 reduced to background levels. This trend was accompanied by gradually decreasing levels of inflammatory cytokines (Fig. 1C). Importantly, DCV treatment had no noticeable effects on hepatic marker expression or functions (Fig. S1O–Q).

In vehicle-treated cultures, HSC activation markers continued to rise, whereas in DCV-treated cultures they peaked at Day 0 and then decreased significantly, reaching very low levels comparable to qHSCs by Day 12 (Fig. 1D). These findings indicate that HCV infection clearly induces HSC activation, while curing HCV reduces activation, suggesting a possible HSC reversion. This is further supported by the progressive decline in cytokines known to promote HSC activation (Fig. 1E), reinforcing the notion that HCV cure likely induces HSC reversion.

### Detailed characterization of reverted HSCs

**Fig. 2:**
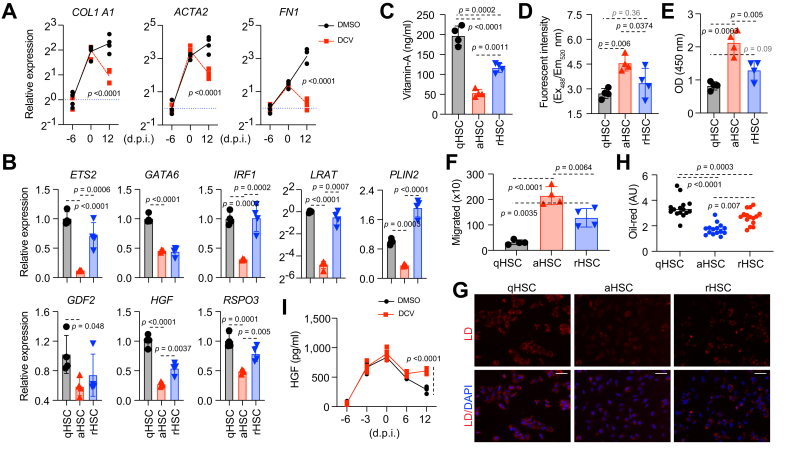
Detailed characterization of reverted HSCs. In the experiments described in Fig. 1A, qHSCs were isolated from uninfected liver cultures; aHSCs and rHSCs were from infected liver cultures treated with DMSO or DCV (10 nM). (A) Transcript analysis of activation marker by qRT-PCR (n = 4, mean ± SD, ANOVA). (B) Transcript analysis of selected genes in qHSC, aHSC, and rHSC by qRT-PCR (n =, mean ± SD, ANOVA). Purified qHSC, aHSC, and rHSC were quantified for vitamin A by ELISA (C), reactive oxygen species (ROS) by DCFH-DA fluorescence assay (D), BrdU incorporation assay (E), and PDGF-BB-induced cell migration assay (F) (n = 4, mean ± SD, ANOVA). (G-H) Oil-red signals of purified qHSC, aHSC, and rHSC quantified using ImageJ (1.52k) (Scale bars, 100 μm, ANOVA). (I) ELISA analysis of HGF in cell culture supernatants (n = 4, mean ± SD, ANOVA). aHSCs: activated hepatic stellate cells; COL1A1, collagen type I alpha 1 chain; DCV, daclatasvir; ETS1/ETS2, ETS proto-oncogene 1/ETS proto-oncogene 2; FN1, fibronectin; GATA6, GATA binding protein 6; GDF2/BMP9, growth differentiation factor 2; HGF, hepatocyte growth factor; IRF1/IRF2, interferon regulatory factor 1/2; LRAT, lecithin-retinol acyltransferase; PDGFB, platelet derived growth factor subunit B; PLIN2, perilipin 2; qHSCs: quiescent hepatic stellate cells; qRT-PCR, quantitative real time-PCR; rHSCs, reverted hepatic stellate cells; RSPO3, R-spondin 3.

The above findings suggest that following HCV cure, aHSCs undergo reversion to a less activated state. Although protein levels of activation markers return to near-quiescent levels, their transcript levels remain elevated except *FN1* (Fig. 2A). This indicates that reverted human HSCs are transcriptionally—and likely functionally—distinct from qHSCs, reflecting a partial but incomplete reversion to the quiescent state, consistent with findings from mouse models.^8^^,^^9^

To further characterize these reverted HSCs (rHSCs), we first compared their expression of key genes important for HSC quiescence^13^ and homeostatic and hepatoprotective functions[14], [15], [16], [17], [18] with those in naïve qHSCs. Many genes were comparable to those observed in qHSCs (Fig. 2B and S3A), however, some exhibited either no restoration or incomplete restoration (Fig. 2B and S3A).

Next, we performed functional characterizations to compare rHSCs with quiescent and activated HSCs. Compared with aHSCs, rHSCs exhibited higher levels of vitamin A, although these levels remained significantly lower than those observed in qHSCs (Fig. 2C). They also showed higher ROS levels than qHSCs but slightly lower than aHSCs (Fig. 2D). Similarly, rHSCs displayed intermediate levels of proliferation and migration between qHSCs and aHSCs (Fig. 2E and F).

In addition to these bulk analyses, we performed a microscopic assessment of rHSCs at single-cell resolution. We stained lipid droplets, where vitamin A is stored, to evaluate heterogeneity within the rHSC population (Fig. 2G). Consistent with the bulk vitamin A quantification, Oil-red staining generally followed the same trend (Fig. 2H). However, when examining individual cells, we observed clear heterogeneity in red signal intensity within the rHSC population. Some cells displayed red signal levels comparable to qHSCs, while others resembled aHSCs (Fig. 2G). This is further supported by quantification of intracellular lipid using flow cytometry analysis (Fig. S3B). By contrast, before DCV treatment, the majority of HSCs display collagen type I alpha 1 chain (COL1A1)^+^ and alpha-smooth muscle actin (α-SMA)^+^ in immunostaining. Flow cytometry for α-SMA similarly showed a highly uniform positive population, indicating limited heterogeneity at baseline (Fig. S3C and D). These findings indicate that rHSCs undergo partial restoration of quiescent characteristics yet retain functional properties that are intermediate between quiescent and activated states.

Finally, we assessed the paracrine effects of rHSCs on co-cultured hepatocytes and macrophages. Following DCV treatment, *HGF* transcripts increased in rHSCs (Fig. 2B), partially restoring culture-wide HGF levels (Fig. 2I). Accordingly, hepatocytes and macrophages exhibited upregulation of canonical HGF-MET proto-oncogene, receptor tyrosine kinase (MET) target genes (Fig. S3E and F).

### Single-cell RNA sequencing analysis to compare activated and reverted HSCs

Given the heterogeneous features of rHSCs, we performed single-cell RNA sequencing (scRNA-seq) to further investigate their molecular characteristics. As shown in Fig. S4A, at 12 days post DCV treatment, we prepared two control liver cultures treated with DMSO and two experimental liver cultures treated with DCV for scRNA-seq analysis. Across the four groups, a total of 15,218 individual HSCs were analyzed, with an average sequencing depth of 39,473 reads per cell (Fig. S4A).

**Fig. 3:**
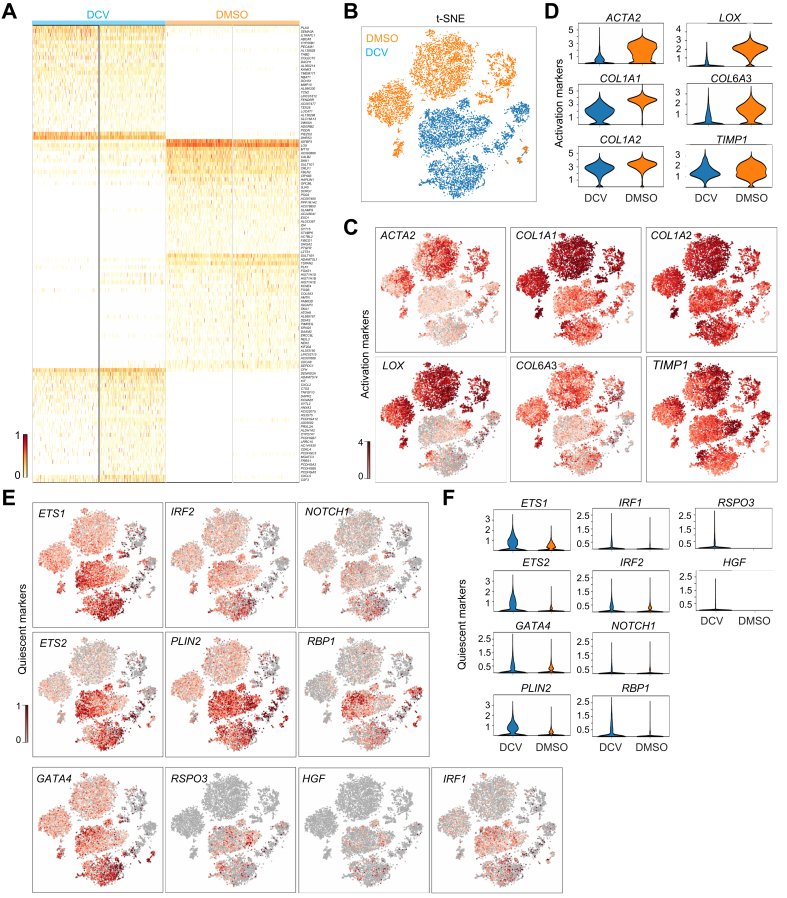
Single-cell RNA sequencing analysis to compare activated and reverted HSCs. (A) Heatmap comparing differentially expressed genes between DMSO- and DCV-treated HSC populations with (*p* <0.05) and (log_2_ fold change >0). The maximum number of marker genes (30) was selected per group. (B) *t*-SNE visualization of DMSO- and DCV-treated cells. *t*-SNE and violin plots were generated based on individual activation markers (C,D), quiescent and homeostatic markers (E,F). All scRNA-seq analyses were generated with the BBrowserX software (BioTuring Inc., San Diego, CA, USA). COL1A1/COL1A2/COL6A3, collagen type I alpha 1 chain/alpha 2 chain/collagen type VI alpha 3; DCV, daclatasvir; ETS1/ETS2, ETS proto-oncogene 1/ETS proto-oncogene 2; FN1, fibronectin; GATA4, GATA binding protein 4; HGF, hepatocyte growth factor; HSCs, hepatic stellate cells; IRF1/IRF2, interferon regulatory factor 1/2; NOTCH, Notch receptor 1; PLIN2, perilipin 2; RBP1, retinol-binding protein 1; RSPO3, R-spondin 3; scRNA-seq, single-cell RNA sequencing; *t*-SNE, *t*-distributed Stochastic Neighbor Embedding.

Global transcriptomic analysis revealed distinct differences between DMSO and DCV groups (Fig. 3A). To evaluate the global and local clustering patterns, we generated Uniform Manifold Approximation and Projection (Umap) and *t*-distributed Stochastic Neighbor Embedding (t-SNE) plots. The Umap plot showed that DMSO- and DCV-treated cells clustered together in one large region (Fig. S4B), indicating a high degree of similarity in the global expression pattern between the two groups.

Distinct cluster separations between DMSO and DCV groups were observed in the t-SNE plots (Fig. 3B), indicating local differences between the two groups. We also identified a small mixed cluster at the bottom right side of the t-SNE plot, comprising both DMSO- and DCV-treated cells (Fig. S4C) as a hepatocyte contaminant resulting from HSC purification from liver cultures (Fig. S4D).

Using Louvain clustering, the DMSO group was subdivided into five distinct clusters (Fig. S4E). However, transcriptomic analysis focusing on HSC activation and quiescence markers revealed that these clusters expressed comparable levels of activation markers, while showing consistently low expression of quiescent markers (Fig. S4F). To further compare HSC status between the treatment groups, we selected a panel of well-established activation markers and assessed their expression levels using t-SNE visualization (Fig. 3C). For more quantitative analysis, we generated violin plots for each activation marker individually (Fig. 3D). The analysis revealed that all activation markers exhibited higher expression levels in the DMSO compared with the DCV group.

In contrast, HSC quiescent markers were expressed at higher levels in the DCV compared with the DMSO group (Fig. 3E and F), indicating a more quiescent-like phenotypes. These results were further supported by GO analysis, which demonstrated that the DMSO group exhibited significantly higher enrichment for GO terms associated with HSC activation and associated functionalities (Fig. S4G).

Finally, to further validate our findings, we compared our data with a published human liver scRNA-seq dataset.^19^ Reverted hiPSC-derived HSCs (rHSCs; DCV group) clustered closer to patient quiescent HSCs (pHSCs) than hiPSC-derived activated HSCs (aHSCs; DMSO group), as evidenced by shorter distances in t-SNE space and concordant expression patterns in gene sets related to collagen regulation (Fig. S5A and B). Consistently, rHSCs in the DCV group display quiescence markers at levels comparable to pHSCs and clearly higher than aHSCs, whereas activation markers are markedly lower in both rHSCs and pHSCs relative to aHSCs (Fig. S5C–F).

These results collectively confirm the effectiveness of DCV treatment in reverting activated HSCs, as evidenced by the downregulation of activation markers and upregulation of quiescent markers.

### Single-cell RNA sequencing analysis of reverted HSCs

**Fig. 4:**
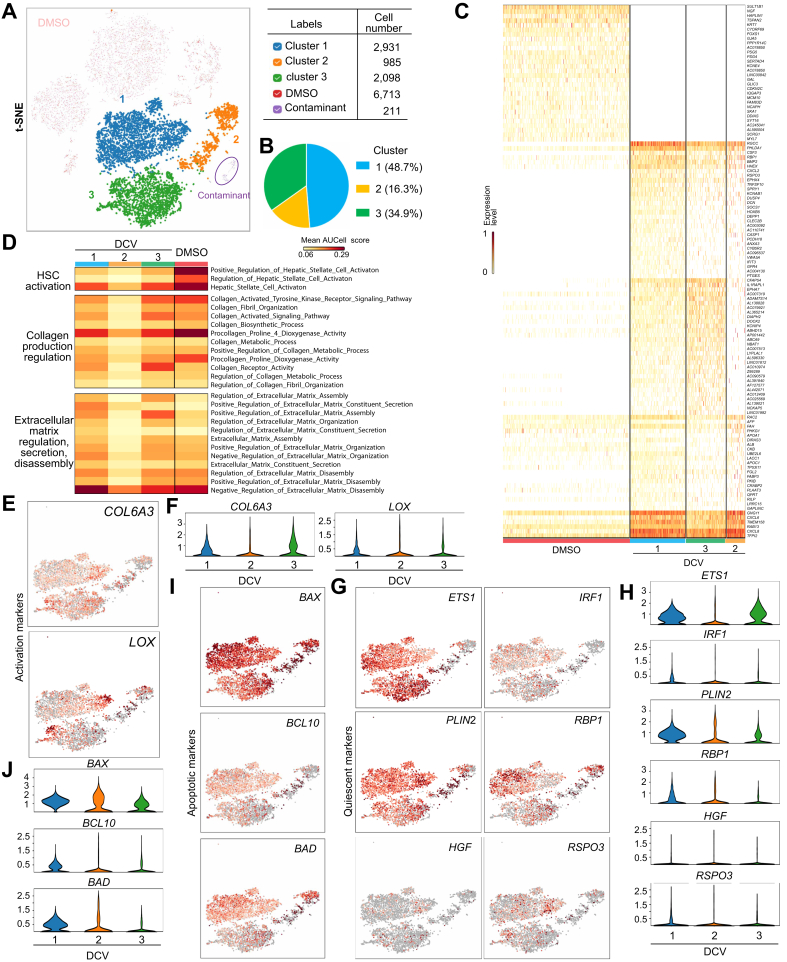
Single-cell RNA sequencing analysis of reverted HSCs. (A) t-SNE plot with three rHSC subclusters, grouped via the Louvain clustering method (resolution = 0.1). (B) Pie chart of the rHSC subclusters. (C) Heatmap of differentially expressed genes between DMSO and rHSC subclusters. (D) GO enrichment test comparing DMSO to rHSC subclusters. t-SNE and violin plots demonstrating relative expression levels of activation markers (E,F), quiescence-associated markers (G,H), and apoptotic markers (I,J). COL1A1/COL1A2/COL6A3, collagen type I alpha 1 chain/alpha 2 chain/collagen type VI alpha 3; DCV, daclatasvir; ETS1/ETS2, ETS proto-oncogene 1/ETS proto-oncogene 2; GO, Gene Ontology; HGF, hepatocyte growth factor; HSCs, hepatic stellate cells; LOX, lysyl oxidase; PLIN2, perilipin 2; RBP1, retinol-binding protein 1; rHSCs, reverted hepatic stellate cells; RSPO3, R-spondin 3; *t*-SNE, *t*-distributed Stochastic Neighbor Embedding.

Next, we used the same Louvain clustering to divide the DCV group into three distinct clusters (Fig. 4A and B). Global transcriptomic analysis revealed that all three DCV clusters exhibited distinct gene expression profiles compared with the DMSO group (Fig. 4C). Notably, clusters 1 and 3 shared a higher number of commonly expressed genes compared with cluster 2 (Fig. 4C and S6A). This trend was further supported by GO analysis, indicating a greater similarity between clusters 1 and 3 (Fig. 4D). Further examination of the GO plot revealed that cluster 2 had the lowest expression levels of genes associated with HSC activation or activation-related processes (Fig. 4D). These analyses suggest that cluster 2 represents the least activated and likely the most reverted HSC group among the three DCV clusters.

In rodent models of fibrosis, aHSCs have been shown to undergo apoptosis, senescence, or a less activated, quiescent-like state.^8^^,^^9^ Based on this paradigm, we focused on evaluating HSC quiescent and activation markers, as well as reversion-associated apoptotic and senescent markers.^1^ Analysis of activation markers revealed that cluster 2 contained fewer cells expressing these markers compared with clusters 1 and 3 (Fig. 4E,F and S6B,C), consistent with the GO analysis (Fig. 4D).

When analyzing quiescent markers, we observed mixed outcomes. Some quiescent markers showed higher expression levels in cluster 2 compared with the other clusters, while others exhibited relatively low expression in cluster 2 (Fig. 4G,H and S6D,E). Further analysis of HSC reversion-associated markers, including apoptotic, senescent genes, and senescence-associated secretory phenotype (SASP) genes, revealed substantial heterogeneity across the three clusters (Figs. 4I,J and S6F–K).

Overall, these scRNA-seq analyses highlight the remarkable heterogeneity of rHSCs, revealing that the reversion process does not uniformly restore quiescence. Instead, rHSCs encompass a spectrum of cellular states, likely reflecting varying degrees of activation, quiescence, apoptosis, and senescence.

### Identification of a small population within rHSCs resembling quiescent HSCs

**Fig. 5:**
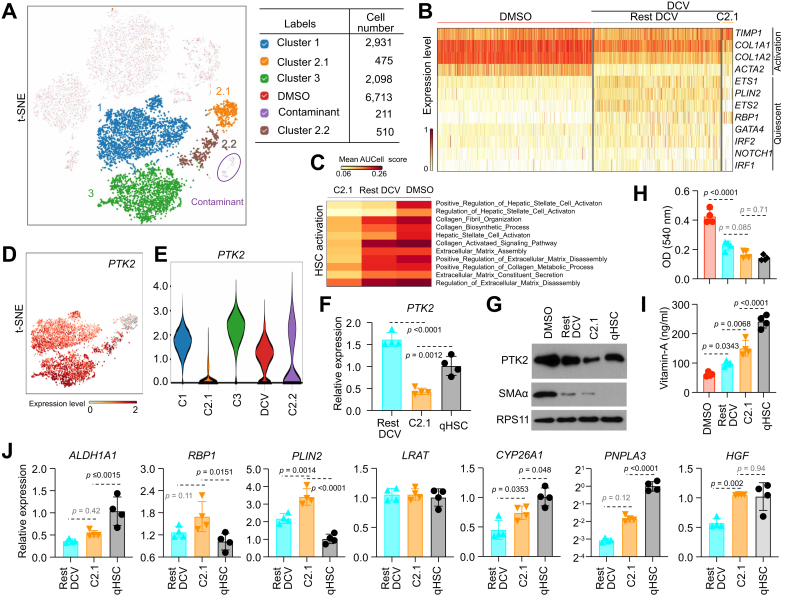
Identification of a small population within rHSCs resembling quiescent HSCs. (A) t-SNE plot of subcluster 2.1 and 2.2. (B) Heatmap of HSC activation and quiescent markers. (C) GO enrichment test between DMSO, cluster 2.1 (C2.1), and the remaining DCV population (Rest DCV). t-SNE plot (D) or violin plot (E) of expression levels of PTK2 in different DCV clusters. PTK2 in indicated cell groups was analyzed by qRT-PCR (F, n = 4, mean ± SD, ANOVA) and Western blot (G). Analysis of collagen levels (H), and intracellular vitamin A levels (I) in indicated cell groups by hydroxyproline assay and ELISA, respectively (n = 4, mean ± SD, ANOVA). (J) Transcript levels of selected genes related to vitamin A metabolism and storage by qRT-PCR (n = 4, mean ± SD, ANOVA). α-SMA/ACTA2, alpha-smooth muscle actin; COL1A1/COL1A2/COL6A3, collagen type I alpha 1 chain/alpha 2 chain/collagen type VI alpha 3; DCV, daclatasvir; ETS1/ETS2, ETS proto-oncogene 1/ETS proto-oncogene 2; GATA4, GATA binding protein 4; GO, Gene Ontology; HSCs, hepatic stellate cells; IRF1/IRF2, interferon regulatory factor 1/2; NOTCH, Notch receptor 1; PLIN2, perilipin 2; PTK2, protein tyrosine kinase 2; qHSCs, quiescent hepatic stellate cells; qRT-PCR, quantitative real time-PCR; Rest DCV, remaining cells in the DCV group; RBP1, retinol-binding protein 1; rHSCs, reverted hepatic stellate cells; *t*-SNE, *t*-distributed Stochastic Neighbor Embedding.

As cluster 2 exhibited the lowest levels of activation markers, we focused on cluster 2 for further analysis. We utilized Louvain clustering to further subdivide cluster 2 into two distinct subclusters, named cluster 2.1 and cluster 2.2 (Fig. 5A). Global transcriptomic analysis revealed that cluster 2.1 displayed distinct gene expression profiles compared with the other clusters (Fig. S7A). To gain further insights, we focused on genes associated with HSC activation and quiescence, which revealed a similarly distinct expression profile in cluster 2.1 (Fig. S7B). Cluster 2.1 exhibited higher levels of quiescent markers and lower levels of activation markers, suggesting that cluster 2.1 may represent a more quiescent-like cell population. This was further supported by a direct comparison between cluster 2.1 and the remaining cells in the DCV group (Rest DCV) (Fig. 5B). Additionally, GO analysis revealed a distinct gene expression profile in cluster 2.1, characterized by the lowest expression of genes associated with HSC activation and ECM regulation (Fig. 5C).

To identify cellular markers that could distinguish cluster 2.1 from the rest of the DCV group, we performed a deeper transcriptomic comparison between these two populations. This analysis identified *PTK2* gene as a suitable marker to differentiate cluster 2.1 from other cells (Fig. 5D and E). PTK2 is expressed at significantly higher levels in aHSCs than in qHSCs (Fig. S7C and D).

Because PTK2 is not a cell-surface protein, it cannot be used directly for cell purification. We therefore examined its relationship to intracellular lipid, a hallmark of qHSCs, within the DCV population. Flow cytometry revealed a ∼10% of lipid-high, PTK2-low subset (Fig. S7E), closely matching the proportion of cluster 2.1 observed by single-cell analysis with the DCV group (Fig. 5A). We accordingly sorted the top ∼10% lipid-high cells and operationally defined them as the C2.1 fraction for downstream analyses. Comparing PTK2 expression between the C2.1 fraction and the Rest DCV population revealed significantly lower PTK2 at both the transcript and protein levels in C2.1, further validating the isolation strategy (Fig. 5F,G and S7F).

Next, we performed functional characterizations to compare C2.1 with the Rest DCV cells. C2.1 exhibited collagen levels comparable to qHSCs (Fig. 5H) and higher vitamin A levels compared with the Rest DCV cells, with levels approaching those of qHSCs (Fig. 5I).

We further compared genes involved in vitamin A metabolism and storage between the two populations.^20^ Consistent with the analysis shown in Fig. 5B, both populations expressed *RBP1* and *PLIN2* at relatively higher levels compared with qHSCs. Notably, other genes were also expressed at higher levels in C2.1, making their expression profiles closer to qHSCs (Fig. 5J and S7G).

Together, these findings indicate that C2.1 exhibits features more closely resembling qHSCs, particularly with respect to collagen levels, vitamin A content, and the gene expression profiles related to vitamin A metabolism and storage.

### rHSCs are more sensitive to re-stimulation

Seeing similarities between C2.1 and naïve qHSCs, we next asked what distinguishes rHSCs from naïve qHSCs. A key factor was their sensitivity to re-stimulation. In mouse carbon tetrachloride (CCl_4_)-induced fibrosis models, reverted HSCs respond more strongly to fibrogenic signals than naïve qHSCs.^8^^,^^9^ To determine whether this heightened sensitivity also occurs in human rHSCs, we compared the responsiveness of human rHSCs and qHSCs.

**Fig. 6:**
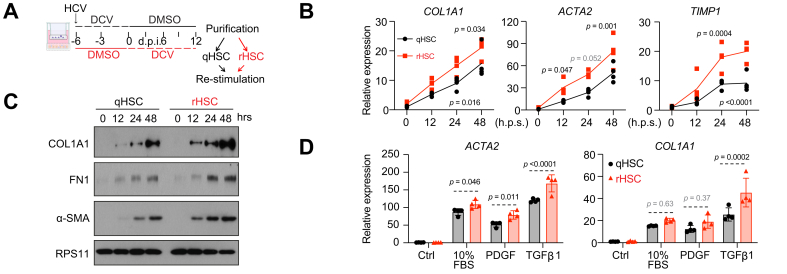
rHSCs are more sensitive to re-stimulation. (A) A schematic overview of HCV infection, DCV treatment, cell purification, and re-stimulation. Transcript levels of selected activation markers by qRT-PCR (B, n = 4, mean ± SD, ANOVA) or for the selected activation marker and housekeeping protein RPS11 by Western blot (C). (D) Transcript levels of activation markers ACTA2 and COL1A1 at 48 h post 10% FBS, PDGF-BB, or TGFβ1 treatment (n = 4, mean ± SD, ANOVA). α-SMA/ACTA2, alpha-smooth muscle actin; COL1A1/COL1A2/COL6A3, collagen type I alpha 1 chain/alpha 2 chain/collagen type VI alpha 3; DCV, daclatasvir; FN1, fibronectin; PDGFB, platelet derived growth factor subunit B; qRT-PCR, quantitative real time-PCR; rHSCs, reverted hepatic stellate cells; TGFβ1, transforming growth factor beta 1.

As shown in Fig. 6A, rHSCs were isolated from liver cultures infected with HCV and then cured with DCV, while control qHSCs came from cultures exposed to HCV in the presence of DCV for the same duration. Both HSC types were plated on Matrigel-coated dishes and treated with supernatant from HCV-infected cultures to assess activation. Consistent with findings from mouse models,^8^^,^^9^ human rHSCs—despite expressing activation markers at levels comparable to qHSCs—displayed a heightened response, as evidenced by increased transcript and protein levels of activation markers (Fig. 6B and C). To investigate whether this enhanced sensitivity is specific to stimuli encountered previously, we conducted similar experiments using various new stimuli. Remarkably, rHSCs also exhibited a stronger response to these novel stimuli (Fig. 6D).

These results suggest that human rHSCs, similar to their mouse counterparts, exhibit an intrinsically heightened sensitivity to both previously encountered and novel fibrogenic stimuli, highlighting the persistent priming effect of prior activation.

### Critical roles of macrophages in HSC reversion

Given the complexity and heterogeneity of rHSCs, we sought to identify key factors that drive their reversion across the full DCV population. Because HSCs constantly interact with neighboring cells in the liver, we examined whether these interactions influence HSC reversion. In mouse CCl_4_-induced fibrosis models, depleting Kupffer cells delays ECM degradation and impairs HSC reversion, indicating their active role in fibrosis resolution.^21^ However, whether human macrophages/Kupffer cells similarly promoter HSC reversion following the removal of the causative agents remains unclear.

**Fig. 7:**
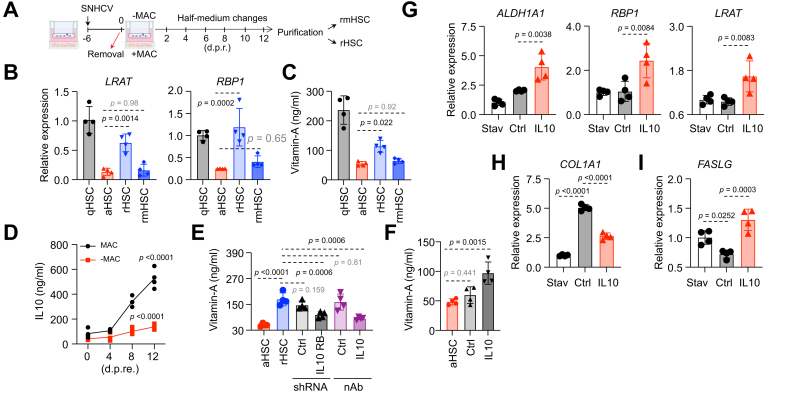
Critical roles of macrophages in HSC reversion. (A) A schematic of supernatant treatment, macrophage removal, cell purification, and re-stimulation. (B) Transcript levels of *LRAT* and *RBP1* in indicated cell groups by qRT-PCR (n = 4, mean ± SD, ANOVA). (C) ELISA analysis of intracellular vitamin A levels in indicated cell groups (n = 4, mean ± SD, ANOVA). (D) ELISA analysis of IL-10 secretion post macrophage removal (n = 4, mean ± SD, ANOVA). (E) ELISA analysis of the intracellular vitamin A levels in HSCs (n = 4, mean ± SD, ANOVA) purified after the following treatments: HCV-infected liver cultures with 10 nM BMS-790052, followed by switch of basal medium, shRNA transduction, and re-seeding in previous liver culture with IL-10 blocking antibody at medium switch. (F) ELISA analysis of vitamin A levels in HSCs (n = 4, mean ± SD, ANOVA) purified after the following treatments: HCV-infected liver culture with 10 nM BMS-790052, followed by BSA or 40 ng/ml IL-10. (G-I) qHSCs were activated by culturing in 10% FBS, then serum starved before re-exposure to 10% FBS, with BSA or IL-10. At 48 h, cells were analyzed for transcript levels of genes associated with vitamin A metabolism and storage (G), activation markers (H), and apoptotic markers (I) by qRT-PCR (n = 4, mean ± SD, ANOVA). aHSC, activated hepatic stellate cells; ALDH1A1, aldehyde dehydrogenase 1 family member a1; COL1A1, collagen type I alpha 1 chain; FASLG, fas ligand; HSCs, hepatic stellate cells; LRAT, lecithin-retinol acyltransferase; qHSCs, quiescent hepatic stellate cells; qRT-PCR, quantitative real time-PCR; RBP1, retinol-binding protein 1; rHSCs, reverted hepatic stellate cells; rmHSC, reverted hepatic stellate cells from MASLD; shRNA, short hairpin RNA.

To test this, we treated multicellular liver cultures with conditioned supernatant from HCV-infected cultures. DCV was included throughout to eliminate any residual infectivity, ensuring the supernatant served only as an activating stimulus for HSCs rather than causing new infection. On Day 6, the supernatant was replaced with basal medium to mimic a rapid ‘HCV cure’, under two conditions: macrophages retained or macrophages depleted. Twelve days after macrophage manipulation, HSCs were purified and designated as rHSCs (macrophages retained) and rmHSCs (reverted HSCs after macrophage depletion) (Fig. 7A). We analyzed the expression of markers related to HSC quiescence and homeostatic functions, and found that macrophage removal markedly impaired the restoration of these markers (Fig. 7B and S8A), indicating a failure of HSC reversion. This was further supported by vitamin A quantification, which showed that rmHSCs exhibited comparable vitamin A levels to aHSCs (Fig. 7C).

Kupffer cell polarization plays a crucial role in regulating HSC activation and deactivation.^22^ Studies show that M2-polarized Kupffer cells secrete IL-10, which can trigger apoptosis in M1 cells and help maintain the M1/M2 balance.^23^ This balance is critical for controlling liver inflammation and fibrosis. However, it is still unclear whether Kupffer cell-derived IL-10 directly promotes the reversion of activated HSCs to a quiescent state.

Following ‘HCV cure’ (Fig. 7A), we observed a steady increase in IL-10 secretion within the liver cultures, with macrophages as the predominant source (Fig. 7D and S8B). Using lentivirus-based shRNA, we downregulated IL-10 receptor (*IL1*0RB) in HSCs within liver cultures (Fig. S8C). Vitamin A quantification revealed that blocking IL-10 signaling in HSCs impaired the reversion of aHSCs, resulting in reduced vitamin A levels (Fig. 7E). A similar inhibitory effect was observed with an IL-10 neutralizing antibody (Fig. 7E and S8D). Conversely, the addition of exogenous IL-10 to liver cultures slightly increased vitamin A levels (Fig. 7F).

To assess physiological relevance of this finding, we analyzed published liver transcriptomic datasets spanning patients with HCV infection with and without antiviral therapy and cohorts with metabolic dysfunction-associated steatotic liver disease (MASLD) and alcohol-associated liver disease (ALD).[24], [25], [26] Hepatic *IL10* transcripts increased after anti-HCV treatment and declined with disease progression in MASLD and ALD (Fig. S8E), consistent with a possible role for IL-10 in promoting HSC reversion.

Finally, to elucidate the mechanism, we treated hiPSC-derived aHSCs with IL-10 and found that several genes related to vitamin A metabolism and storage were upregulated in response to IL-10 (Fig. 7G). Under the same conditions, IL-10 treatment downregulated the transcript levels of activation markers, while upregulating apoptotic markers (Fig.7H and I). Activated primary human HSCs showed similar IL-10 responses, albeit with donor-to-donor variability (Fig. S8F–H).

Together, these findings suggest that Kupffer cell-derived IL-10 plays a direct role in promoting HSC reversion, likely by inducing the expression of vitamin A metabolism-related genes and modulating the expression of activation and apoptotic markers.

### Reversion of MASLD-activated HSCs

**Fig. 8:**
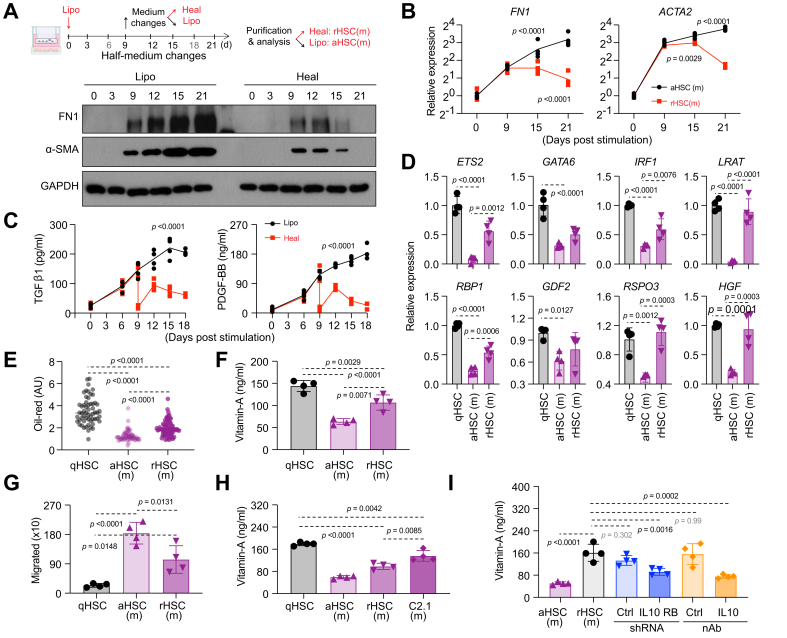
Reversion of MASLD-activated HSCs. (A) Top: Schematic of treatment and medium changes. For controls, aHSCs(m) were from lipotoxic milieu cultures; qHSCs from healthy milieu cultures. Bottom: HSCs were collected for Western blot analysis. (B) Analysis of the HSC activation marker transcripts by qRT-PCR (mean ± SD, n = 4). (C) ELISA analysis of selected cytokines (n = 4, mean ± SD, ANOVA). (D) Transcript levels of selected quiescence-related genes in qHSC, aHSC(m), and rmHSC by qRT-PCR (n = 4, mean ± SD, ANOVA). (E–G) Oil-red signals (E), quantification of vitamin A (F), and PDGF-BB-induced cell migration assay (G) of purified qHSC, aHSC(m), and rHSC(m) (n = 4, mean ± SD, ANOVA). (H) Quantification of intracellular vitamin A (n = 4, mean ± SD, ANOVA). (I) Quantification of intracellular vitamin A in HSCs (n = 4, mean ± SD, ANOVA) purified after the following treatments: lipotoxic milieu-treated liver cultures, followed by healthy milieu, shRNA-IL10 transduction, and re-seeding in previous liver culture with IL-10 neutralization at medium switch (n = 4, mean ± SD, ANOVA). aHSCs, activated hepatic stellate cells; α-SMA/ACTA2, alpha-smooth muscle actin; ETS2, ETS proto-oncogene 2; FN1, fibronectin; GAPDH, glyceraldehyde 3-phosphate dehydrogenase; GATA6, GATA binding protein 6; GDF2/BMP9, growth differentiation factor 2; HGF, hepatocyte growth factor; HSCs, hepatic stellate cells; IRF1, interferon regulatory factor 1; LRAT, lecithin-retinol acyltransferase; MASLD, metabolic dysfunction-associated steatotic liver disease; PDGFB, platelet derived growth factor subunit B; qHSCs, quiescent hepatic stellate cells; qRT-PCR, quantitative real time-PCR; RBP1, retinol-binding protein 1; rmHSC, reverted hepatic stellate cells from MASLD; RSPO3, R-spondin 3; TGFβ1, transforming growth factor beta 1.

Beyond HCV infection, multiple conditions can trigger HSC activation and liver fibrosis—notably MASLD. In our multicellular liver model, a lipotoxic milieu elicited MASLD-like phenotypes, including robust HSC activation.^10^ To test whether ‘treating’ this MASLD-like state promotes HSC reversion, we replaced the lipotoxic milieu with a healthy medium and performed the same analyses described above to assess reversion (Fig. 8A).

Following healthy medium replacement, activation markers in HSCs from the MASLD model (rmHSC) declined progressively, reaching levels comparable to naïve qHSCs by Day 12 (Fig. 8A and B). This reversion was accompanied by decreases in profibrotic cytokines (Fig. 8C), partial-to-complete restoration of quiescence-associated markers (Fig. 8D and S9A), and upregulation of canonical HGF-MET target genes in hepatocytes and macrophages (Fig. S9B and C). rmHSC also displayed heterogeneity in intracellular lipid content (Fig. 8E and S9D,E) and showed normalization toward qHSC baselines for intracellular lipids, vitamin A stores, and cell motility (Fig. 8E–G), paralleling the phenotype observed after HCV cure.

Using the same gating strategy, we identified within the reverted population a lipid-high, PTK2-low subset (∼20%), termed C2.1(m) (Fig. S9F and G). This subset exhibited higher vitamin A content (Fig. 8H), lower collagen expression (Fig. S9H), and elevated transcripts of genes involved in vitamin A metabolism and homeostatic/hepatoprotective functions (Fig. S9I), consistent with a more quiescent state, analogous to that observed after HCV cure.

Finally, blocking IL-10 signaling—either by *IL1*0RB downregulation in HSCs or with an IL-10–neutralizing antibody—impaired restoration of vitamin A in the MASLD reversal paradigm, indicating that IL-10 similarly supports reversion in this context (Fig. 8I).

## Discussion

Our study establishes that hiPSC-derived HSCs activated by either HCV infection or MASLD-like lipotoxic stress can undergo reversion when the injurious stimulus is removed. By integrating functional assays with scRNA-seq, we demonstrate that rHSCs exhibit a hybrid phenotype—partially restoring quiescent-like features while retaining distinct characteristics that differentiate them from naïve qHSCs. Importantly, we demonstrate that macrophage-derived IL-10 is a key regulator of this process, acting through transcriptional programs that enhance vitamin A metabolism and suppress fibrogenic activation.

By extending prior rodent studies^8^^,^^9^ to a human-relevant system, we highlight both the parallels and unique features of HSC plasticity. Similar to mouse models, rHSCs exhibit a ‘primed’ phenotype with incomplete transcriptional reversion and heightened sensitivity to re-stimulation. Our single-cell analysis further reveals that reversion produces a heterogeneous landscape, spanning cells with quiescent, apoptotic, or senescent signatures. Notably, a distinct subset (lipid-high, PTK2-low), ranging from 10% to 20%, more closely resembles naïve qHSCs, suggesting that a fraction of rHSCs may achieve higher restoration. Identifying and enriching this population could be key to therapeutic strategies that drive fibrosis regression more effectively.

The incorporation of both HCV and MASLD into our model underscores the generalizability of HSC reversion across etiologies. Withdrawal of lipotoxic stress led to phenotypic and functional reversion highly analogous to that observed after HCV cure. These results reinforce that HSC plasticity is not limited to viral injury, but extends to metabolic liver disease. Moreover, the observation that IL-10 promotes reversion in both contexts emphasizes its centrality as a mediator of fibrosis regression.

Despite these restorative tendencies, rHSCs remain transcriptionally and functionally distinct from naïve qHSCs. Elevated transcript levels of activation markers and incomplete restoration of certain quiescence-related factors indicate that reversion does not fully erase the molecular imprint of prior activation. Functionally, rHSCs exhibit intermediate levels of ROS, proliferation, and migration compared to quiescent and activated states, reflecting a poised phenotype.

A striking feature of rHSCs is their heightened sensitivity to re-stimulation. This enhanced responsiveness mirrors observations in CCl_4_-induced fibrosis mouse models,^8^^,^^9^ suggesting that prior activation likely leaves a lasting epigenetic or transcriptional memory in HSCs. This finding has significant clinical implications: while fibrosis may regress following the removal of causative agents, the liver may remain predisposed to rapid fibrogenesis upon re-exposure to injury. This vulnerability underscores the need for strategies that not only promote reversion, but also mitigate this primed state to prevent fibrosis recurrence.

The roles of human macrophages in facilitating HSC reversion emerged as a pivotal finding. Depletion of macrophages impaired the restoration of quiescent markers and vitamin A levels, while IL-10—a key cytokine secreted by M2-polarized macrophages—directly promoted reversion. These results build on rodent studies implicating Kupffer cells in fibrosis resolution^21^ and provide a mechanistic link in humans: IL-10 signaling enhances HSC plasticity, tipping the balance toward deactivation. This macrophage-HSC crosstalk highlights the multicellular nature of fibrosis resolution and suggests that therapeutic modulation of macrophage polarization or IL-10 delivery could amplify reversion, offering a novel avenue for antifibrotic interventions.

Although our hiPSC-derived liver culture system recapitulates key aspects of human liver biology,^10^ it has several limitations. It lacks the full complexity of the *in-vivo* microenvironment, such as endothelial cells, immune cells beyond macrophages, and systemic influences.^27^ Using HCV and MASLD as stimuli captures important disease aspects but does not cover all fibrogenic causes, such as alcohol, HBV, drugs/toxins. The heterogeneity of rHSCs, although a strength of our single-cell approach, also complicates the identification of uniform therapeutic targets. Lastly, the short culture duration raises the possibility that incomplete reversion of rHSCs may reflect insufficient recovery time. Still, by 12 days post-HCV cure, at the protein level, the expression of key activation markers returns to levels comparable to those observed in naïve qHSCs, suggesting a substantial degree of reversion within this timeframe.

Looking forward, our findings open several research avenues. Elucidating the epigenetic mechanisms underlying the heightened sensitivity of rHSCs to re-stimulation could reveal targets to ‘reset’ their primed state. Further exploration of C2.1’s quiescent-like properties—potentially through lineage tracing or advanced sorting techniques—may clarify whether these cells represent a stable endpoint of reversion or a transient state. In addition, modulating the expression of key transcription factors—particularly those not fully restored in rHSCs (*e.g.* GATA binding protein 4 [*GATA4*], peroxisome proliferator-activated receptor gamma [*PPARG*])—may offer strategies to promote a more complete return to quiescence. Finally, validating the IL-10-mediated reversion pathway in patient-derived samples or *in-vivo* models could accelerate its translation into clinical strategies.

In conclusion, this study demonstrates that activated human HSCs process significant reversion capacity across multiple etiologies, driven in part by macrophage-derived IL-10, yet they remain distinct from naïve quiescent HSCs due to their heterogeneity and sensitivity to re-stimulation. These insights advance our understanding of HSC plasticity and suggest that strategies enhancing reversion while overcoming residual priming may pave the way for durable fibrosis resolution in patients.

## Abbreviations

α-SMA/ACTA2, alpha-smooth muscle actin; aHSCs, activated hepatic stellate cells; ALD, alcohol-associated liver disease; ALDH1A1, aldehyde dehydrogenase 1 family member a1; ASGR1, asialoglycoprotein receptor 1; BMM, basal maintenance medium; CCl_4_, carbon tetrachloride; CITI, Cleveland Clinic Center for Immunotherapy and Precision Immuno-Oncology; COL1A1/COL1A2/COL6A3, collagen type I alpha 1 chain/alpha 2 chain/collagen type VI alpha 3; CYP26A1, cytochrome p450 family 26 subfamily a member 1; DCV, daclatasvir; ECM, extracellular matrix; EGF, epidermal growth factor; ETS1/ETS2, ETS proto-oncogene 1/ETS proto-oncogene 2; FASLG, fas ligand; FN1, fibronectin; GAPDH, glyceraldehyde 3-phosphate dehydrogenase; GATA4, GATA binding protein 4; GATA6, GATA binding protein 6; GDF2/BMP9, growth differentiation factor 2; GO, Gene Ontology; HGF, hepatocyte growth factor; hiPSC, human induced pluripotent stem cells; HPC, high performance computing; HSCs, hepatic stellate cells; IL10RB, interleukin 10 receptor beta; IRF1/IRF2, interferon regulatory factor 1/2; KOSR, knockout serum replacement; LOX, lysyl oxidase; LRAT, lecithin-retinol acyltransferase; MASLD, metabolic dysfunction-associated steatotic liver disease; MDA, malondialdehyde; MET, MET proto-oncogene, receptor tyrosine kinase; NOTCH, Notch receptor 1; NS5A, nonstructural protein 5A; PDGFB, platelet derived growth factor subunit B; pHSCs, patient quiescent HSCs; PLIN2, perilipin 2; PPARG, peroxisome proliferator-activated receptor gamma; PTK2, protein tyrosine kinase 2; qHSCs, quiescent hepatic stellate cells; RBP1, retinol-binding protein 1; Rest DCV, remaining cells in the DCV group; rHSCs, reverted hepatic stellate cells; rmHSC, reverted hepatic stellate cells from MASLD; ROS: reactive oxygen species; RSPO3, R-spondin 3; SASP, senescence-associated secretory phenotype; scRNA-seq, single-cell RNA sequencing; shRNA, short hairpin RNA; SPARCL1, SPARC like 1; TGFβ1, transforming growth factor beta 1; TNFα: tumor necrosis factor-alpha; *t*-SNE, *t*-distributed Stochastic Neighbor Embedding; Umap, Uniform Manifold Approximation and Projection.

## Financial support

XJW, EHH, LB, ZYF, FZ, KOS, LW, AH, YFZ, WBC, and XFW were supported by 10.13039/100000002National Institutes of Health grants R00-AI141742 (XFW), DP2-AI170515 (XFW), R01-DK125100 (subcontract to XFW), and R01- AA031226 (XFW), and by Seed Fund (XFW) provided by the 10.13039/100007312Cleveland Clinic Foundation. YJZ was supported by the 10.13039/100020950Department of Pathology 10.13039/100008564School of Medicine, Case Western Reserve University and 10.13039/100012324University Hospitals Cleveland Medical Center. The funders had no role in study design, data collection and analysis, decision to publish, or preparation of the manuscript.

## Authors’ contributions

Study concept and design, XJW, EHH, XFW. Methodology, XJW, EHH, LB, YJZ, XFW. Analysis and interpretation of data, XJW, EHH, XFW. Acquisition of data, XJW, EHH, LB, FZ, ZYF, AH, YFZ, LW, WBC, XFW. Drafting of the manuscript, XJW, EHH, XFW. Critical revision of manuscript, XJW, EHH, LB, FZ, KOS, ZYF, LW, AH, WBC, YJZ. Funding acquisition, XFW. Supervision, XFW.

## Data availability

The single-cell RNA sequencing data have been deposited in the Gene Expression Omnibus (GEO) under accession GSE304675. All analysis code—including R scripts, shell (sh) scripts, and HPC-associated workflows—have been released on GitHub at the time of publication. Upon publication, interactive plots and analyses will also be made publicly available on BioTuring (Talk2Data).

## Conflicts of interest

The authors declare that they have no conflict of interest.

Please refer to the accompanying ICMJE disclosure forms for further details.
