# Supplementary material for: Single-cell analysis of heterogeneity in reverted hiPSC-derived human hepatic stellate cells
Source: JHEP Rep. 2025 Nov 10;8(2):101669. doi: 10.1016/j.jhepr.2025.101669 (PMC12803900; doi:10.1016/j.jhepr.2025.101669)
Supplement: Multimedia component 1 [file mmc1.docx]

**Single-Cell Analysis of Heterogeneity in Reverted hiPSC-Derived Human Hepatic Stellate Cells**

Xinjia Wang, Eun Hee Ha, Lu Bian, Zhuoying Feng, Fan Zhang, Kyle O’Shaughnessy, Lei Wang, Andrea Hochwald, Yifei Zheng, Weibo Chen, Yujie Zhang, and Xianfang Wu

Table of contents

Supplementary materials and methods ...........................................................................2

Supplementary Fig. S1.....................................................................................................9

Supplementary Fig. S2...................................................................................................11

Supplementary Fig. S3...................................................................................................12

Supplementary Fig. S4...................................................................................................13

Supplementary Fig. S5...................................................................................................14

Supplementary Fig. S6...................................................................................................15

Supplementary Fig. S7...................................................................................................17

Supplementary Fig. S8...................................................................................................18

Supplementary Fig. S9...................................................................................................20

**SUPPLEMENTARY MATERIALS AND METHODS**

***Stem cell maintenance and differentiation***.

Human induced pluripotent stem cells (hiPSCs) including iPSC-9 and iPSC-W3 were cultured on growth factor-reduced matrigel according to the manufacturer’s recommendations in a feeder-independent mTeSR1-based medium (Stemcell Technologies, Canada). Cultures were replenished with fresh medium daily. Cells were passaged every 4 - 6 days as clumps using ReLeSR (Stemcell Technologies, Canada). For all the experiments in this study, hiPSCs were used between passages 30 and 40.

The two iPSC lines iPSC-9 and iPSC-W3 were generated from de-identified fibroblast cells purchased from a commercial supplier (ATCC, Virginia, USA) and their use was reviewed and approved by Cleveland Clinic Institutional Biosafety Committee (IBC 2101). Key experimental findings obtained from both iPSCs are comparable. Data shown in this manuscript are from iPSC-W3 line.

***Establishment of a multicellular coculture****.*

hiPSC-derived hepatocytes, HSCs, and macrophages were differentiated separately and assembled into a coculture as described previously ^[10]^. Cocultures were maintained in a basal maintenance medium (BMM) that is consisted of glucose-free DMEM (Thermo Fisher Scientific, Massachusetts, USA) supplemented with 4% knockout serum replacement (KOSR, Thermo Fisher Scientific, Massachusetts, USA), 2% B-27 (Thermo Fisher Scientific, Massachusetts, USA), 3U/ml heparin (Sigma-Aldrich, Missouri, USA), 200μg/ml Transferrin (Sigma-Aldrich, Missouri, USA), 30ng/ml EGF (Peprotech, New Jersey, USA), 5μM retinol (Sigma-Aldrich, Missouri, USA), and 0.5μM dexamethasone (Sigma-Aldrich, Missouri, USA). The 12-well plate containing the cocultures was placed on an orbital shaker platform at a speed of 30 rpm/min. The coculture was replenished with fresh medium every two days, or as indicated in the figure legend.

***Separation of hepatocytes from hepatic stellate cells****.*

Hepatocytes were isolated from cocultures as described previously ^[10]^. Briefly, hiPSC-derived co-cultures of hepatocytes and HSCs were washed once with Versene and then incubated in Versene at 37°C for 20–25 minutes to loosen cell–cell contacts. Cultures were subsequently washed with pre-warmed DMEM/F12 (Thermo Fisher Scientific, Massachusetts, USA) and incubated with a pre-warmed collagenase mixture (2.0 mg/ml collagenase, 1.0 mg/ml dispase, 100 U/ml DNase, 0.2% DMSO in HBM, all from Sigma-Aldrich) at 37°C for 35–40 minutes. During this incubation, cells were gently pipetted to disrupt clumps and facilitate dissociation. Following digestion, cells were collected by adding Versene and centrifuged at 400 g for 5 minutes at room temperature. The pellet was resuspended in Versene and incubated again at 37°C for 45 minutes. By the end of this incubation, the majority of cells were dissociated into single cells. If necessary, the suspension was passed through a 100 µm cell strainer to remove residual clumps.

To separate hepatocytes from HSCs, single-cell suspensions were first blocked in 1% BSA and incubated on ice for 1 hour. Cells were then stained with mouse anti-ASGR1 antibody (Proteintech, Illinois, USA), followed by incubation with anti-mouse IgG microbeads (Miltenyibiotec, Maryland, USA), and subjected to magnetic separation at 4°C. Hepatocytes were collected from the magnetically bound fraction, while HSCs were obtained from the flow-through after an additional round of hepatocyte depletion. The purified hepatocytes and HSCs were subsequently collected for downstream analysis such as single cell RNA sequencing, immunofluorescent staining, western blot, and RNA extraction.

***Lentivector-based transduction***.

Lentiviral particles-based shRNAs targeting *IL10RB* gene were cloned into the pLKO.1-puro vector using standard techniques. To generate lentiviral particles, packaging plasmids were transfected into Lenti-293T cells using Lipofectamine 2000 (Thermo Fisher Scientific, Massachusetts, USA) according to the manufacturer’s directions, and the medium was changed to DMEM supplement with 3% ESC-qualified FBS (Thermo Fisher Scientific, Massachusetts, USA) at 6 hr post-transfection. The medium containing the lentiviral particles was harvested at 24 and 48 hrs post transfection and filtered through a 0.2 μm filter. Subsequently, lentiviral particles were further concentrated (100:1) using Lenti-X Concentrator (Takara Bio, California, USA) according to the manufacturer’s directions.

To transduce hiPSC-derived HSCs, cells were exposed twice to a mixture of lentivectors and maintenance medium (1:10 ratio) in the presence of polybrene (4μg/ml, Thermo Fisher Scientific, Massachusetts, USA) and a JAK inhibitor (Pyridone 6, 100nM, Tocris Bioscience, Minnesota, USA), with each exposure lasting 4 hrs. Following transduction, thorough washing with pre-warmed DMEM/F12 was performed, and the cells were maintained in complete coculture maintenance medium until the experiments ended.

***Immunofluorescence analysis*.**

Cells were fixed in 4% para-formaldehyde in phosphate-buffered saline (PBS) at room temperature for 10 min and blocked with PBTG (PBS containing 10% normal goat serum (Lampire Biological Laboratories, Pennsylvania, USA), 1% bovine serum albumin (BSA), 0.1% Triton-X100) at room temperature for 2 to 3 hrs. Cells were incubated with primary antibodies (diluted in PBTG) at 4°C overnight or 2 hr at room temperature. Isotype mouse or rabbit IgGs were used as negative controls. After four washes with PBS, Alexa Fluro conjugated secondary antibodies (1:1000 diluted in PBTG, Thermo Fisher Scientific, Massachusetts, USA) were added and incubated in the dark at room temperature for 1 hr, followed by four washes with PBS.

To stain lipid droplets in hiPSC-derived HSCs, purified cells were cultured on a matrigel-coated plate in the respective medium for 6 to 8 hrs. Subsequently, the cells were fixed with 10% formalin for 30 minutes, followed by a 10-minute wash with 60% isopropanol at room temperature. Cells were then stained with 0.18% freshly prepared oil-red (Sigma-Aldrich, Missouri, USA) in PBS for 4 min at room temperature and washed thoroughly with double-distilled water four times. Nuclei were stained with DAPI for 1 min at room temperature. Images were captured using an Olympus IX73 Inverted Fluorescence Microscope System (Tokyo, Japan).

To quantify lipid content at the single-cell level, we analyzed fluorescence microscopy images in which lipid droplets and nuclei were co-stained and captured in a single channel. Using ImageJ software, each image was first converted to grayscale, and nuclei were segmented using Otsu thresholding followed by morphological filtering and watershed-based separation to identify individual cells. We then measured the lipid signal intensity within each segmented nucleus, extracting key parameters including nuclear area, mean lipid intensity, and the range of lipid signal (maximum and minimum intensity) for downstream analysis. Primary antibodies used for Immunofluorescence analysis are listed in **Supplementary CTAT Table**.

***Quantitative real-time RT-PCR (RT-qPCR)****.*

Total RNA was isolated from cell lysates using the RNAeasy Mini Kit (Qiagen, Germany) or PureLink RNA Mini Kit (Thermo Fisher Scientific, Massachusetts, USA) followed by reverse transcription using RevertAid First Strand cDNA Synthesis (Thermo Fisher Scientific, Massachusetts, USA). Gene expression was quantified using the AzuraView GreenFast qPCR Blue Mix (Azura Genomics, Massachusetts, USA) on a LightCycler 480 Instrument (Roche Life Science, Germany) or a QuantStudio3 Instrument (Applied Biosystems, California, USA) with gene-specific primers shown in **Supplementary CTAT Table**.

PCR conditions were as follows: initial denaturation step at 50°C for 2 min and 95°C for 10 min, then 45 cycles of 95°C for 15 sec, 56°C for 15 sec, and 72°C for 20 sec; followed by a melting step of 95°C for 10s, 65°C for 10s and a 0.07°C/s decrease from 95°C; and finally, a cooling step of 50°C for 5s. A melting-curve analysis confirmed PCR product specificity. The fold changes in mRNA expression were determined using the ΔΔCt method relative to the values in control samples as indicated in figure legends, after normalization to housekeeping genes (RPS11 or GAPDH). Unless stated otherwise, results are presented as means ± standard deviation (SD). Comparisons between groups/cells were made using the two-tailed t-test with Welch’s corrections or One-way ANOVA/Tukey’s post-hoc test to calculate exact p-values, unless stated otherwise. Statistical analysis was performed in Graph Pad PRISM 10.

***Western blot analysis***.

Cells were directly lysed in 2x SDS lysis buffer (2mL Tris-HCl (pH 6.8), 50% glycerol, 10% SDS, 0.5% bromophenol blue, and freshly added 10% β-mercaptoethanol) and cell lysates were separated by 7.5%, 10%, or 12% sodium dodecyl sulfate-polyacrylamide gel electrophoresis, in MES or MOPS buffer, followed by transfer onto polyvinylidene fluoride (PVDF) membrane (Sigma-Aldrich, Missouri, USA). GAPDH or RPS11 were used as housekeeping protein controls for comparison between different groups or treatments, as indicated in the figures and figures legends. Primary antibodies used for western blot analysis are listed in **Supplementary CTAT Table**.

***Hepatitis C Virus (HCV) stock***.

The HCV stock was prepared through a sequential process. Initially, the wildtype (WT) Jc1 HCVcc was generated by harvesting supernatant containing viral particles at 72 hours post electroporation of Huh-7.5 cells with *in vitro* transcribed HCV RNA. Subsequently, the WT stock was employed to infect naïve Huh-7.5 cells and subjected to serial passaging, surpassing 40 passages to establish the SAV HCVcc stock. All virus stocks were aliquoted and stored at -80°C. For infection, liver cultures were exposed to HCVcc at the indicated MOIs for 6 hrs. Following this, a thorough washing with DMEM/F12 was conducted, and the cells were then transitioned to a maintenance medium.

***Quantification of HCV infection***.

To quantify intracellular HCV RNA copies, RNA extraction from infected cells was carried out using the RNeasy kit (Qiagen, Germany) or PureLink RNA Mini Kit (Thermo Fisher Scientific), followed by cDNA synthesis using the RevertAid first strand cDNA synthesis kit (Thermo Fisher Scientific). Relative quantification of HCV RNA copies was performed by qPCR using a specific primer pair targeting HCV NS3. The expression levels were normalized to uninfected cells, and the results were presented as fold changes.

***ELISAs***.

To measure the secreted levels of human cytokines, including pro-inflammatory cytokines (IFNβ1, IL6, TNFα, and IL1β), profibrotic cytokines (TGFβ1 and PDGF-BB), and HGF in the liver cultures under different experiment settings, supernatant was collected at indicated time points and cytokine levels were quantified by ELISA kits (R&D Systems, Minnesota, USA), according to the manufacturers’ instructions. To quantify albumin secretion, liver cultures were washed thoroughly with DMEM/F12 medium three times, followed by an incubation with albumin-free medium for 3 hrs; they were then analyzed by ELISA kits (R&D Systems), according to the manufacturers’ instructions. Quantifications of human vitamin A in different HSCs were performed using a human vitamin A ELISA kit (CUSABIO, Texas, USA), according to the manufacturer’ instructions.

***Collagen quantification***.

Intracellular collagen levels in different HSCs were quantified by a colorimetric hydroxyproline assay kit (Sigma-Aldrich, Missouri, USA), according to the manufacturer’s instructions.

***Cell proliferation****.*

The proliferation of different HSCs was assessed using a BrdU cell proliferation assay kit (Cell Signaling Technologies, Massachusetts, USA), according to the manufacturer’s instructions.

***Cellular reactive oxygen species (ROS)****.*

The levels of ROS in different HSCs were assessed using a DCFA/H2DCFDA kit and detected by a VarioSkan LUX Multi-Mode Plate Reader (Thermo Fisher Scientific), following the manufacturers' instructions.

***Flow cytometry***.

Purified reverted HSCs were stained with BioTracker 488 green lipid droplet dye (Sigma-Aldrich, Missouri, USA) for 30 minutes in the dark at 37°C prior to flow cytometry sorting. Based on fluorescence intensity, the cells were sorted into two populations: the top 10% and the remaining 90%. These sorted populations were subsequently subjected to downstream analyses, including qRT-PCR, western blotting, intracellular vitamin A quantification, and collagen measurement.

***Hepatic function assays***.

Hepatic functional characterizations included the quantification of intracellular glycogen levels (Promega, Wisconsin, USA) and extracellular levels of total bile acids (Cell Biolabs, California, USA). These were performed using the purified hepatocytes from liver cultures, following the manufacturers’ instructions.

***Culture of primary human HSCs***.

pHSCs were purchased from Lonza (Pennsylvania, USA) and cultured according to the manufacturer’s instructions using the recommended stellate cell growth medium and supplements. To ensure identity and quality, the purity of pHSCs was assessed by flow cytometry using anti-αSMA staining. Only preparations that displayed high purity were used for experiments. Cells were used between passages 2 and 5 to avoid phenotypic drift and discarded beyond passage 5.

***Induction of MASLD-like phenotypes in liver cultures.***

Liver cultures are maintained in basal medium supplemented with the normal physiological level of insulin (0.7 nM, Sigma-Aldrich, Missouri, USA) and glucose (6 mM, Thermo Fisher Scientific) to mimic healthy condition (healthy) or high levels of insulin (7 nM), glucose (25 nM), and free fatty acids (oleic acid 68 µM and palmitic acid, 45 µM, both from Sigma-Aldrich, Missouri, USA) to mimic plasma concentrations of these factors in MASH patients (lipotoxic) for the indicated days.

**Supplementary Figure S1**

**
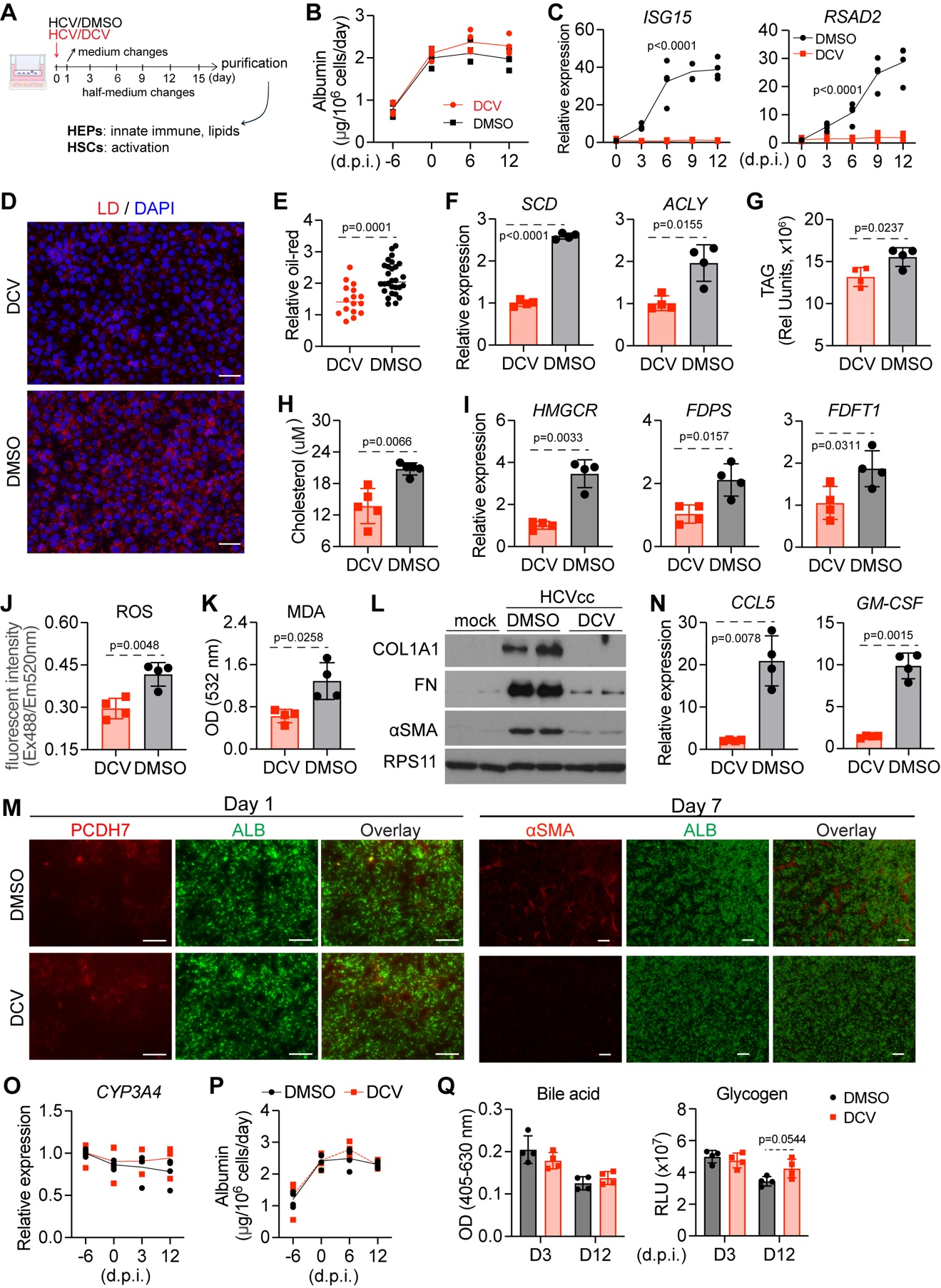
**

**Supplementary Figure S1. Establishment of a human-relevant *in vitro* system to study HSC activation and reversion.**

(**A**). Schematic representation of HCV infection and medium change. Liver cultures were infected with SAV HCVcc (MOI=1.0) in the presence or absence of DCV (BMS-790052, 10nM) for overnight before the inoculum was removed. At day 15 post infection, Hepatocytes (HEPs) and HSCs were purified for downstream analyses.

(**B**). To quantify albumin secretion at the indicated time points, the culture medium was replaced with albumin-free medium for 3 hrs and then analyzed by ELISA (B). Shown are mean ± SD from n=4 independent experiments.

(**C**). Analysis of transcript levels of ISG15 and RSAD2 in hepatocytes purified at the indicated time points by qRT-PCR. Shown are mean ± SD from n=4 independent experiments.

(**D-E**). At day 15 post infection, hepatocytes were purified and re-seeded onto matrigel-coated plates for 4 hrs before cells were fixed for oil-red staining (D, scale bars, 100μm) and red-signals were quantified using ImageJ software (1.52k) (E).

(**F, I**). At day 15 post infection, hepatocytes were purified for analysis of transcript levels of the selected lipogenesis-related genes (F) or cholesterol-related genes (I) by qRT-PCR. Shown are mean ± SD from n=4 independent experiments.

(**G, H**). At day 15 post infection, hepatocytes were purified for analysis of triacylglycerol (TAG, G) or cholesterol (H) by Triglyceride-Glo and Cholesterol-Glo kit, respectively. Shown are mean ± SD from n=4 independent experiments.

(**J-K**). At day 15 post infection, hepatocytes were purified for analysis of reactive oxygen species (ROS) by DCFH-DA fluorescence assay (Ex488/Em520nm, J), and for malondialdehyde (MDA) by ELISA kit. Shown are mean ± SD from n=4 independent experiments.

(**L, N**). At day 15 post infection, HSCs were purified for western blot analysis of activation markers and housekeeping protein RPS11 (L), and for analysis of transcript levels of the selected cytokine genes by qRT-PCR (N). Shown in (N) are mean ± SD from n=4 independent experiments.

(**M**). At day 1 and 7 post infection, liver cultures (the bottom compartment containing hepatocytes and HSCs) were fixed for immunofluorescent staining of HSC marker PCDH7 and α-SMA (Scale bars, 100μm).

(**O-P**). Analysis of transcript levels of *CYP3A4* in hepatocytes purified from the experiments described in Figure 1A by qRT-PCR (O). To quantify albumin secretion at the indicated time points, the culture medium was replaced with albumin-free medium for 3 hrs and then analyzed by ELISA (P). Shown are mean ± SD from n=4 independent experiments.

(**Q**). In the experiments described in Figure 1A, culture supernatants were collected to quantify total bile acids (C). Purified hepatocytes were used to assess intracellular glycogen levels (B). Shown are mean ± SD from n=4 independent experiments.

Statistical analysis was performed using Unpaired t tests with Welch’s correction or One-way ANOVA/Tukey’s post-hoc test to calculate exact p-values.

**Supplementary Figure S2**

**
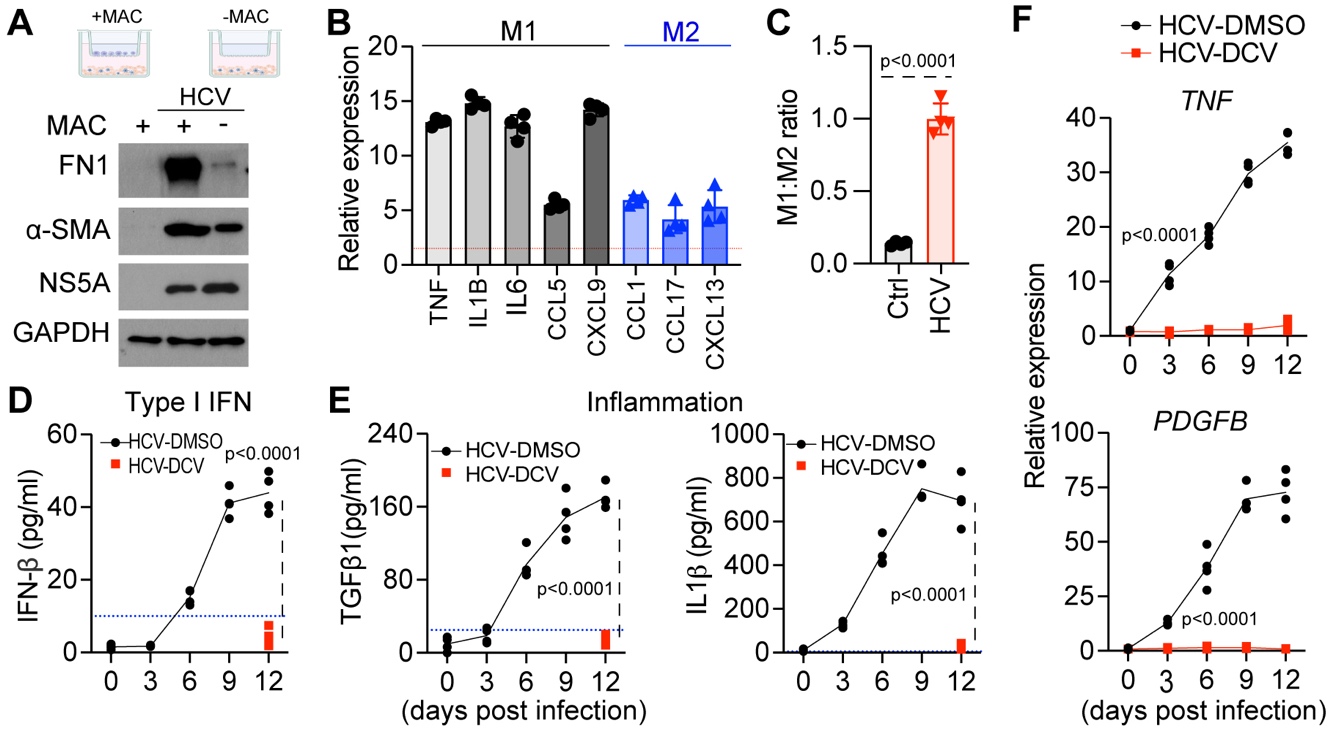
**

**Supplementary Figure S2. Critical roles of macrophages to HCV infection-induced HSC activation.**

(**A**). Top: Schematic representation of liver cultures with or without macrophages. Bottom: Liver cultures were infected with infected with SAV HCVcc (MOI=1.0) for one week and cells from the bottom compartments were collected for western blot analysis of HSC activation markers, HCV NS5A, and housekeeping protein GAPDH.

(**B**). Liver cultures were infected with SAV HCVcc (MOI=1.0) in the presence or absence of DCV (BMS-790052, 10nM) for 7 days. Macrophages were isolated and subject to qRT-PCR analysis of the selected marker genes that are associated with M1 or M2 macrophages. Shown are mean ± SD normalized to uninfected control from 4 independent experiments.

(**C**). Macrophages from the experiments in (B) were analyzed for cell surface markers CD86 (M1) and CD200R (M2) by flow cytometry. The percentages of CD86 or CD200R positive cells from 4 independent experiments were used to calculate M1:M2 ratio.

(**D-E**). From the experiments in (A), supernatants were collected for analysis of the selected cytokines by ELISA. For cultured with DCV, only samples from the last time points were included. Shown are mean ± SD from n=4 independent experiments.

(F). From the experiments in (A), macrophages were collected at the indicated time points for analysis of transcript levels of *TNF* and *PDGFB* by qRT-PCR. Shown are mean ± SD from n=4 independent experiments.

Statistical analysis was performed using Unpaired t tests with Welch’s correction or One-way ANOVA/Tukey’s post-hoc test to calculate exact p-values.

**Supplementary Figure S3**

**
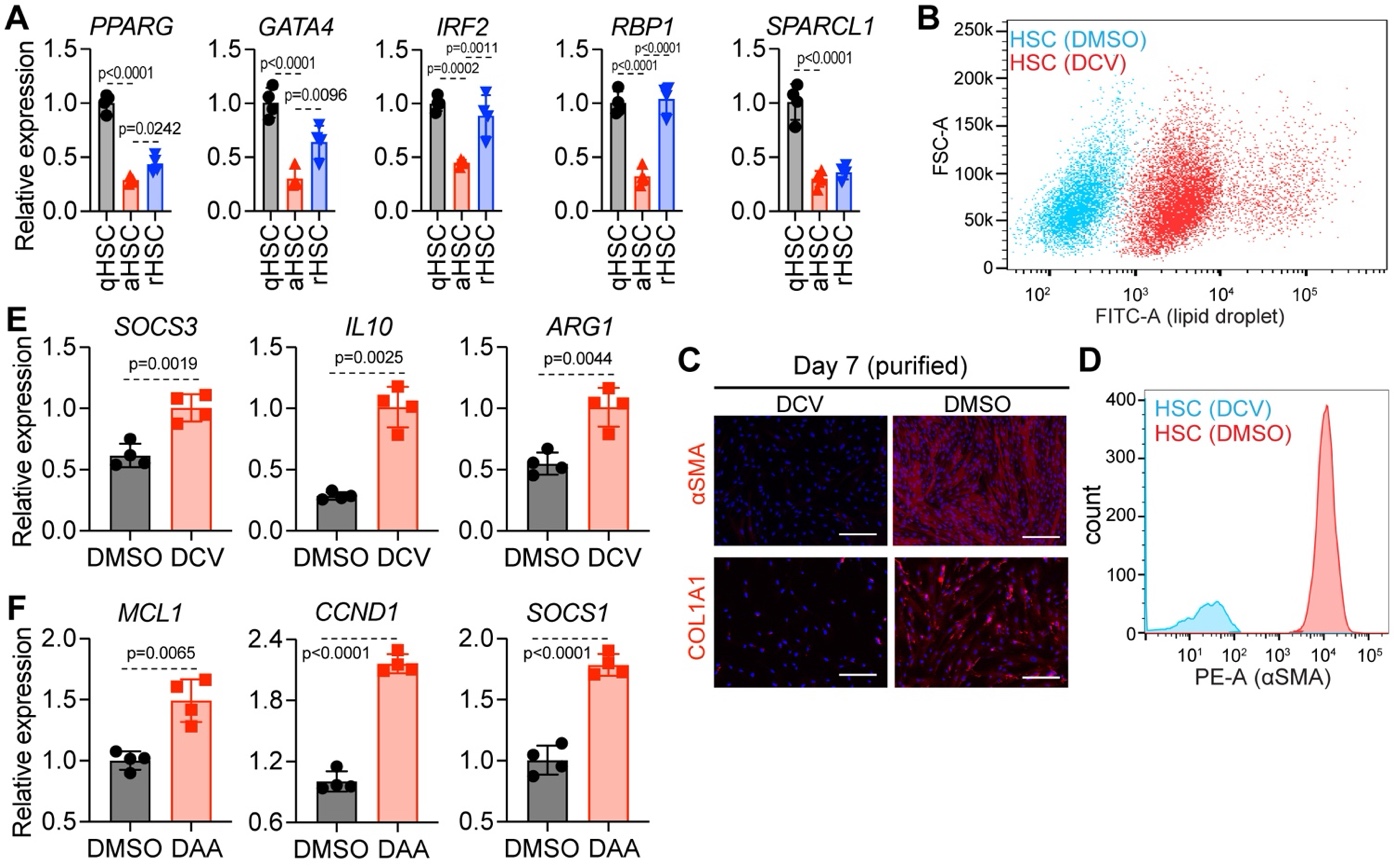
**

**Supplementary Figure S3. Detailed characterization of reverted HSCs.**

In the experiments described in Figure 1A, quiescent HSCs (qHSCs) were isolated from uninfected liver cultures; activated HSCs (aHSCs) were from infected liver cultures treated with DMSO; and reverted HSCs (rHSCs) were infected liver cultures treated with DCV (10nM). All HSCs were purified from liver cultures at 12 days post-treatment.

(**A**). Analysis of transcripts of the selected quiescence-related transcription factor and homeostatic function genes in qHSC, aHSC, and rHSC by qRT-PCR. Shown are mean ± SD from n=4 independent experiments.

(**B**). Representative flow cytometry analysis of intracellular lipid content in HSCs purified from DMSO (aHSCs) and DCV (rHSCs) treated liver cultures at day 12 post-treatment.

(**C-D**). At day 7 post-HCV exposure, HSCs in liver cultures with DMSO and DCV were purified and re-seeded on matrigel or poly-lysine-coated plates for 6 hrs before being fixed for immunofluorescent staining of HSC activation markers (C); or were subjected to flow cytometry analysis of αSMA (D). Nucleus were stained with DAPI (Scale bars, 100μm).

(**E-F**). At day 12 post-treatment, hepatocytes (E) and macrophages (F) were collected for analysis of transcript levels of HGF target genes by qRT-PCR. Shown are mean ± SD from n=4 independent experiments.

Statistical analysis was performed using Unpaired t tests with Welch’s correction or One-way ANOVA/Tukey’s post-hoc test to calculate exact p-values.

**Supplementary Figure S4**

**
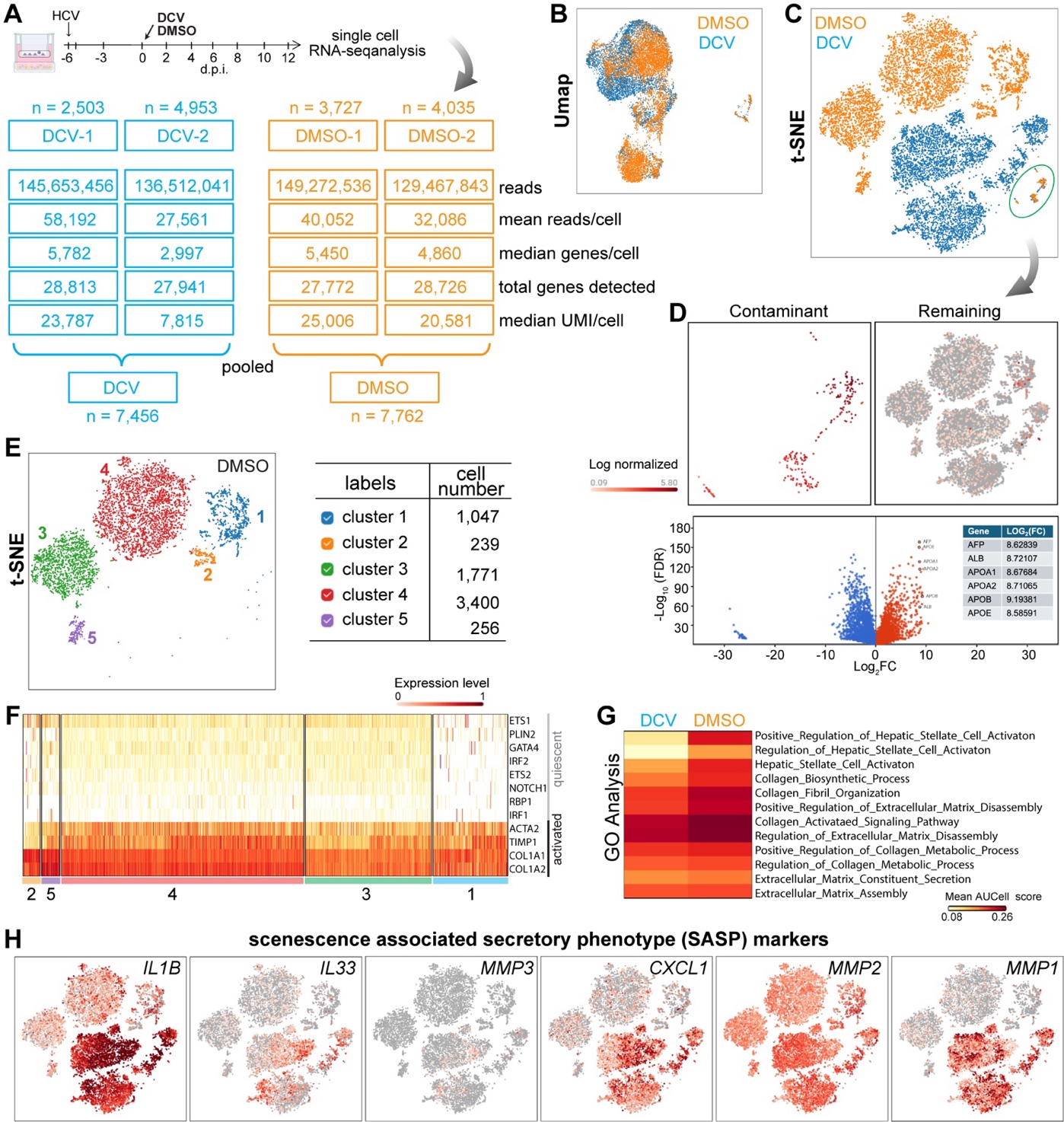
**

**Supplementary Figure S4. Single cell RNA-sequencing analysis to compare activated and reverted HSCs.**

(**A**). Schematic of the timeline of the experiments, including the date of administering DMSO and DCV, along with the subsequent summary consisting of the detailed parameters used for running single cell RNA sequencing analysis.

(**B**). Umap visualization of DMSO and DCV groups.

(**C**). t-SNE visualization of DMSO and DCV groups with the hepatocyte contamination region outlined in green. (**D**). Differential expression analysis between the contamination region and all other remaining single cell RNA sequencing population.

(**E**). t-SNE visualization of DMSO subclustered into five clusters via Louvain sub-clustering method with 0.1 resolution. Among 6,830 DMSO treated cell population, 1,047 cells, 239 cells, 1,771 cells, 3,400 cells, and 256 cells are subclustered from clusters 1 through 5, respectively.

(**F**). Heatmap depicting the gene expression profiles for activation and quiescent markers for the five DMSO subclusters.

(**G**). Gene ontology analysis of DMSO and DCV groups with the selected GO terms associated with HSC activation, collagen, and extracellular matrix.

**Supplementary Figure S5**

**
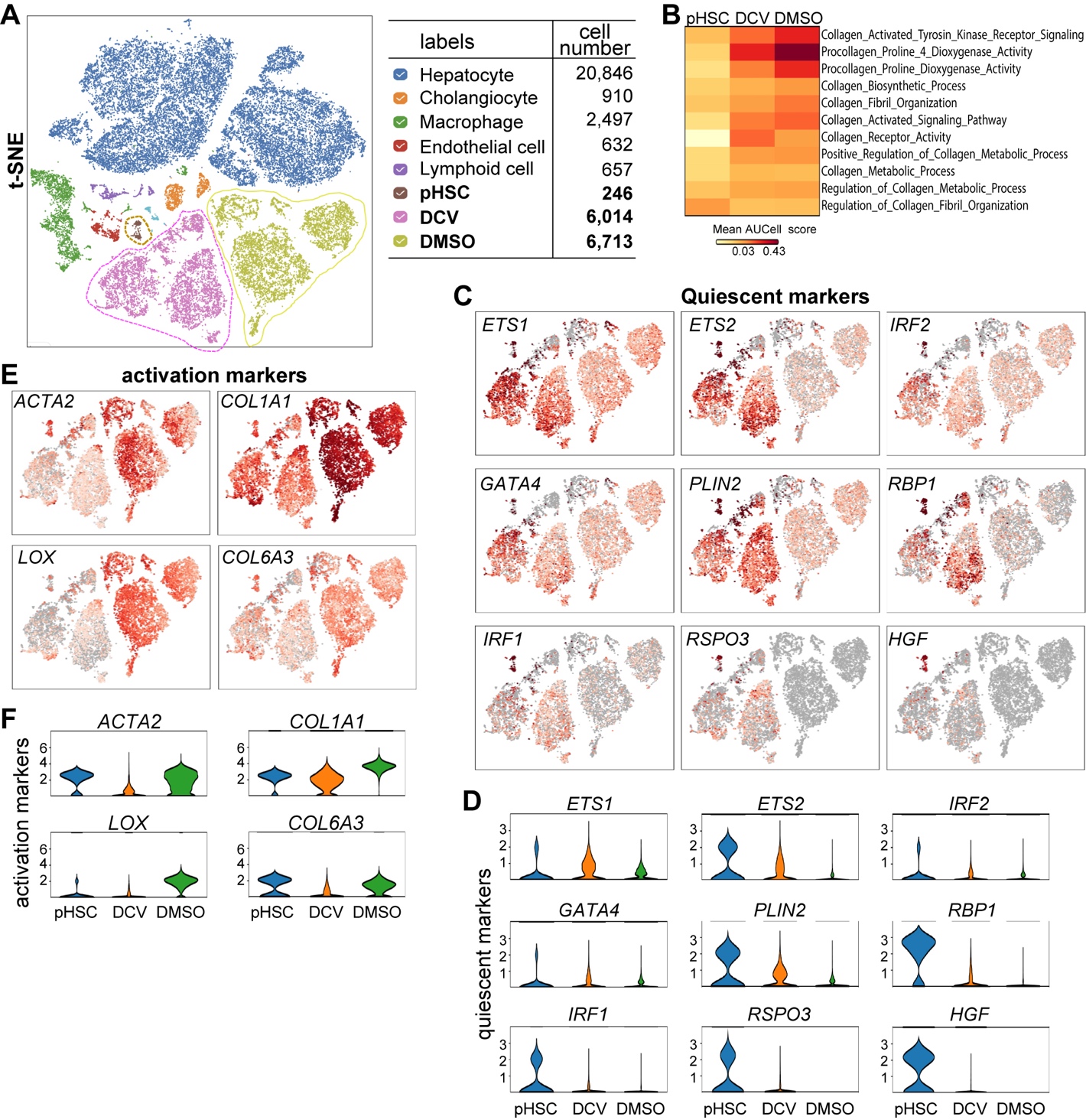
**

**Supplementary Figure S5. Comparison between hiPSC-derived reverted HSCs with primary HSCs.**

(**A**). t-SNE visualization of hiPSC-derived HSCs in DMSO (aHSCs) and DCV (rHSCs) groups with various human hepatic cells (data from GSE158723) including primary HSCs (pHSCs). Different HSC populations were circled in different colors: aHSCs in yellow-green; rHSCs in pink, and pHSCs in brown.

(**B**). Gene ontology enrichment test of collagen production and regulation genes comparing different HSC populations.

(**C-D**). t-SNE plots (C) and violin plots (D) of individual quiescent-related markers and their relative gene expression between different HSC populations.

(**E-F**). t-SNE plots (E) and violin plots (F) of individual activation markers and their relative gene expression between different HSC populations.

**Supplementary Figure S6**


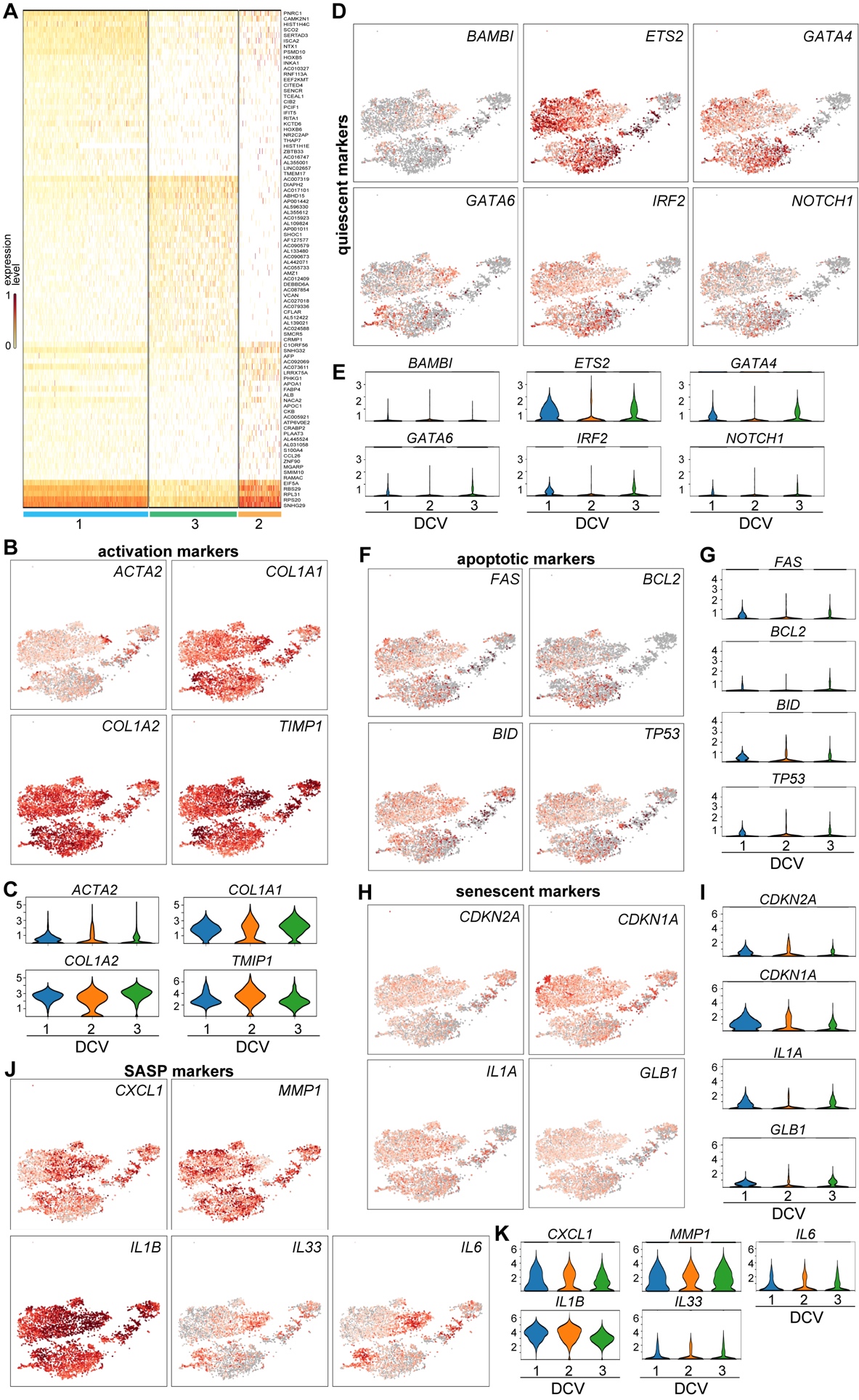


**Supplementary Figure S6. Single cell RNA-sequencing analysis of reverted HSCs.**

(**A**). Heatmap depicting the relative gene expression levels between the three subclusters of the DCV treated group with (p-value<0.05) and (log_2_ fold change>0), as well as the number of marker genes selected to maximum value (30 marker genes per group).

(**B-C**). t-SNE plots (B) and violin plots (C) of individual activation markers and their relative gene expression between different clusters within DCV group.

(**D-E**). t-SNE plots (D) and violin plots (E) of individual quiescent-related markers and their relative gene expression between different clusters within DCV group.

(**F-G**). t-SNE plots (F) and violin plots (G) of individual apoptosis-related markers and their relative gene expression between different clusters within DCV group.

(**H-I**). t-SNE plots (H) and violin plots (I) of individual senescence-related markers and their relative gene expression between different clusters within DCV group.

(**J-K**). t-SNE plots (J) and violin plots (K) of individual SASP-related markers and their relative gene expression between different clusters within DCV group.

**Supplementary Figure S7**

**
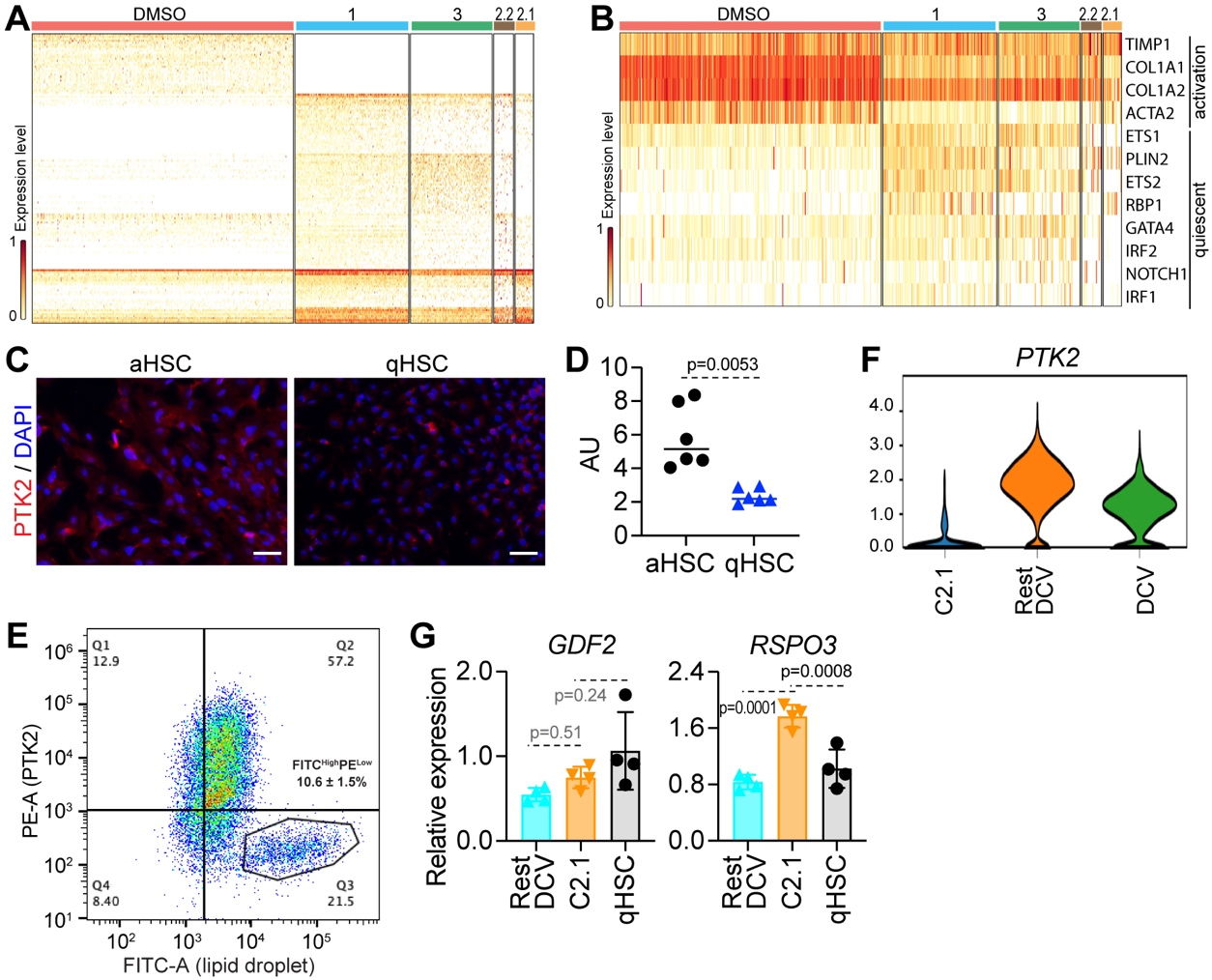
**

**Supplementary Figure S7. Identification of a small population within rHSCs resembling quiescent HSCs.**

(**A**). Heatmap demonstrating differentially expressed genes between DMSO and the four subclusters of DCV, with (p-value<0.05) and (log_2_ fold change>0), and number of marker genes set to maximum per group (30 genes).

(**B**). Heatmap demonstrating the difference in gene expression levels of HSC activation and quiescent markers between DMSO and the four subclusters of DCV.

(**C-D**). Representative immunofluorescent staining of PTK2 in hiPSC-derived activated HSCs (aHSC, activated by culturing in 10% FBS for 4 days) and quiescent HSCs (qHSC) (C, scale bars, 100μm) and red signals were quantified using ImageJ software (1.52k) (D, arbitrary unit/AU).

(**E**). Representative flow cytometry analysis of intracellular lipid content (FITC channel) and PTK2 (PE channel) of reverted HSCs (rHSCs). A subset of lipid-high, PTK2-low cells was selected, with percentage shown from n=3 independent experiments.

(**F**). Violin plots of PTK2 levels in different HSC populations.

(**G**). Analysis of transcript levels of the selected genes related to HSC homeostatic functions in the indicated cell groups by qRT-PCR. Shown are mean ± SD from n=4 independent experiments.

Statistical analysis was performed using Unpaired t tests with Welch’s correction or One-way ANOVA/Tukey’s post-hoc test to calculate exact p-values.

**Supplementary Figure S8**

**
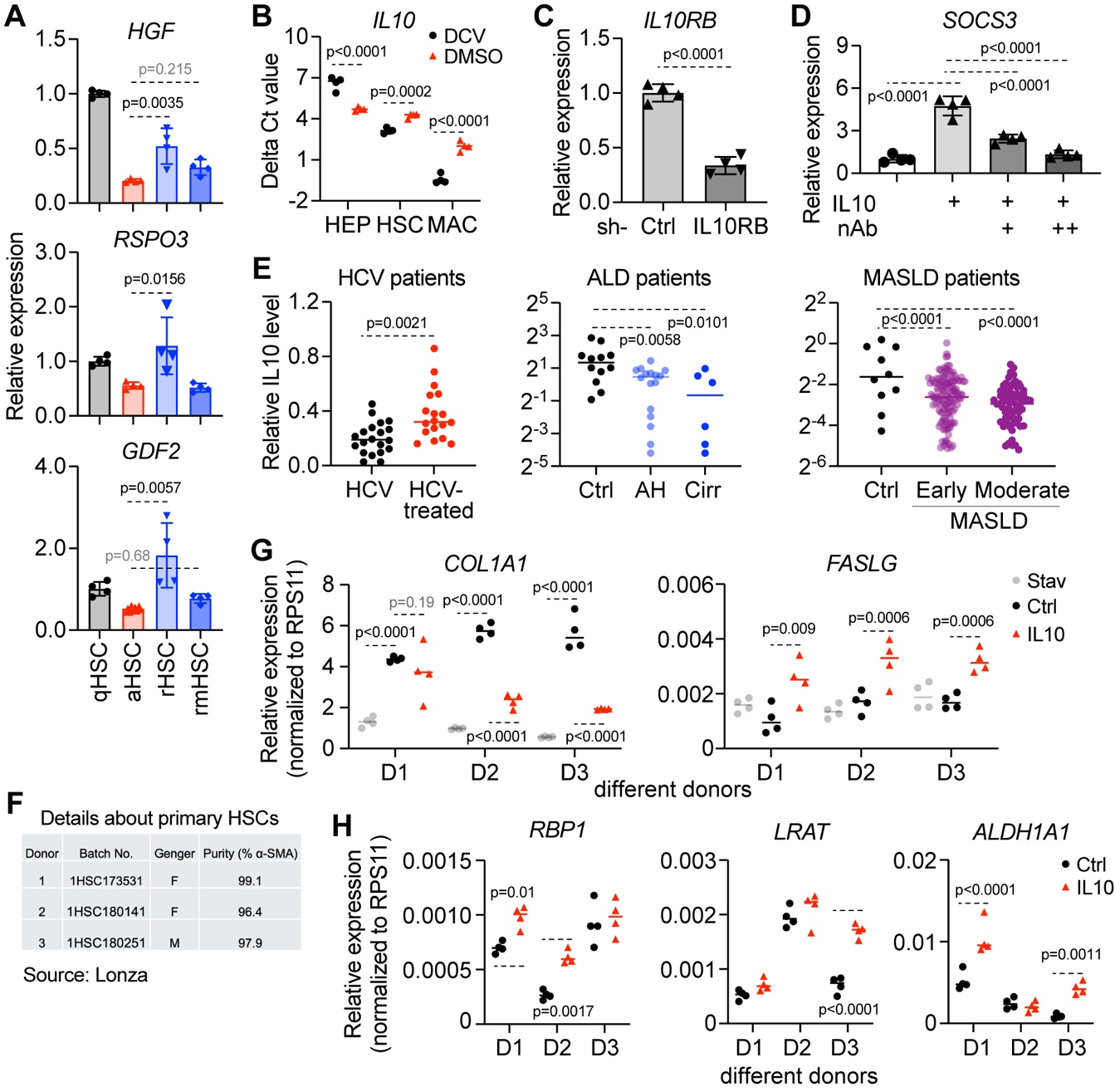
**

**Supplementary Figure S8. Critical roles of macrophages in HSC reversion.**

(**A**). In the experiments shown in Figure 7A, transcript levels of genes related to HSC homeostatic functions including *HGF*, *RSPO3*, and *GDF2* in the indicated cell groups were analyzed by qRT-PCR. Shown are mean ± SD from n=4 independent experiments.

(**B**). In the experiments described Figure S1A, at day 15 post-viral exposure, individual cell types were purified to analyze transcript levels of *IL-10*. Shown are mean ± SD of delta Ct from n=4 independent experiments.

(**C**). hiPSC-derived activated HSCs were transduced with lentivirus-based control shRNA or shRNA against *IL10RB* for 48 hrs before cells were harvested for analysis of *IL10RB* transcript. Shown are mean ± SD from n=4 independent experiments.

(**D**). hiPSC-derived hepatocytes were treated with either BSA control, or IL-10 (20ng/ml) in the presence of increasing concentration of IL-10 neutralizing antibody (0, 100, 300ng/ml) for 24 hrs before cells were collected for analysis of *SOCS3* transcript. Shown are mean ± SD from n=4 independent experiments.

(**E**). Analysis of transcript levels of IL-10 in liver biopsy samples from HCV patients with and without treatment (GSE84346), from MASLD patients (GSE135251), and from ALD patients (GSE142530).

(**F-H**). Activated pHSCs from three different donors (F) were serum starved (stav) for 24 hours before being re-exposed to 10% FBS, in the presence of BSA control or IL10 (40ng/ml). At 48 hours post re-exposure, the cells were collected to analyze the transcript levels of genes associated with activation marker and apoptotic marker (G), and of genes associated with vitamin A metabolism and storage (H), by qRT-PCR. Shown are mean ± SD of expression normalized to RPS11, from n=4 independent experiments.

Statistical analysis was performed using Unpaired t tests with Welch’s correction or One-way ANOVA/Tukey’s post-hoc test to calculate exact p-values.

**Supplementary Figure S9**

**
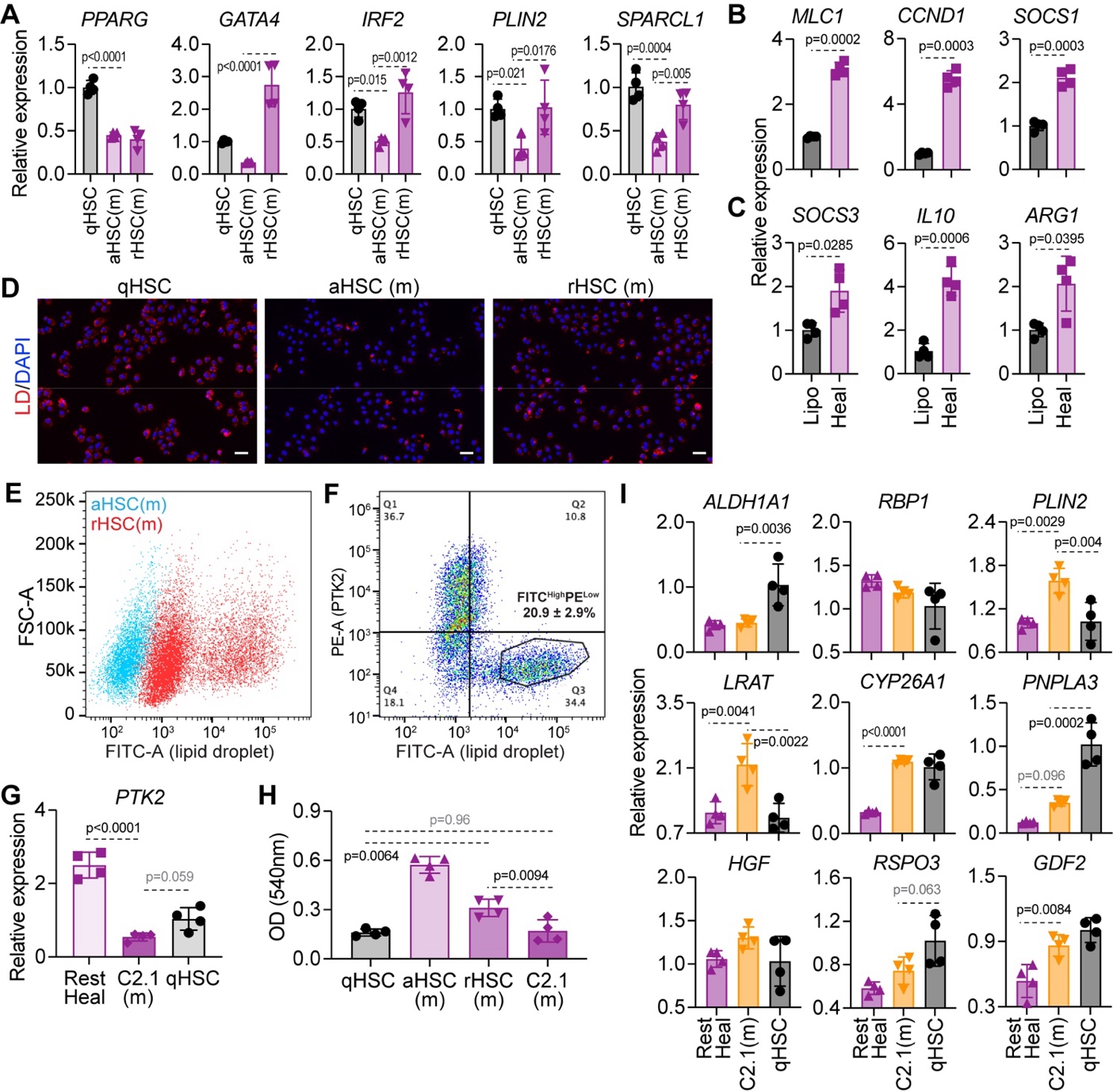
**

**Supplementary Figure S9. Reversion of MASLD-activated HSCs.**

(**A**). In the experiments described in Figure 8A, transcripts of the selected quiescence-related transcription factor and homeostatic function genes in qHSC, aHSC(m), and rHSC(m) were analyzed by qRT-PCR. Shown are mean ± SD from n=4 independent experiments.

(**B-C**). At day 12 post-medium switch, hepatocytes (B) and macrophages (C) were collected for analysis of transcript levels of HGF target genes by qRT-PCR. Shown are mean ± SD from n=4 independent experiments.

(**D**). Purified qHSC, aHSC(m), and rHSC(m) were seeded onto a matrigel-coated plates in the medium from which they were harvested for 6 hrs before cells were fixed for staining of lipid droplets by oil-red.

(**E**). Representative flow cytometry analysis of intracellular lipid content in HSCs purified from lipotoxic (aHSCs(m)) and healthy (rHSCs(m)) treated liver cultures at day 12 post-treatment.

(**F**). Representative flow cytometry analysis of intracellular lipid content (FITC channel) and PTK2 (PE channel) of rHSCs(m). A subset of lipid-high, PTK2-low cells was selected, with percentage shown from n=3 independent experiments.

(**G**). Expression of *PTK2* in the indicated cell groups was analyzed by qRT-PCR. Shown are mean ± SD from n=4 independent experiments.

(**H**). Analysis of collagen levels in the indicated cell groups by the hydroxyproline assay. Shown are mean ± SD from n=4 independent experiments.

(**I**). Analysis of transcript levels of the selected genes related to vitamin A metabolism and storage, as well as HSC homeostatic functions in the indicated cell groups by qRT-PCR. Shown are mean ± SD from n=4 independent experiments.

Statistical analysis was performed using Unpaired t tests with Welch’s correction or One-way ANOVA/Tukey’s post-hoc test to calculate exact p-values.
