# Supplementary material for: Single-cell analysis of heterogeneity in reverted hiPSC-derived human hepatic stellate cells
Source: JHEP Rep. 2025 Nov 10;8(2):101669. doi: 10.1016/j.jhepr.2025.101669 (PMC12803900; doi:10.1016/j.jhepr.2025.101669)
Supplement: Multimedia component 2 [file mmc2.docx]

**JHEP Reports**

**CTAT methods**

Tables for a “Complete, Transparent, Accurate and Timely account” (CTAT) are now mandatory for all revised submissions. The aim is to enhance the reproducibility of methods.

- Only include the parts relevant to your study
- Refer to the CTAT in the main text as ‘Supplementary CTAT Table’
- Do not add subheadings
- Add as many rows as needed to include all information
- Only include one item per row

**If the CTAT form is not relevant to your study, please outline the reasons why:**

|  |
| --- |

- 1. **Antibodies**

| **Name** | **Citation** | **Supplier** | **Cat no.** | **Clone no.** |
| --- | --- | --- | --- | --- |
| Rabbit monoclonal anti-CLO1A1 | PMID: 40634432 | Cell Signaling Technologies | 39952 | E6A8E |
| Rabbit polyclonal anti-COL1A1 | PMID: 32277974 | Rockland antibodies & assays | 600-401-D20 | polyclonal |
| Rabbit polyclonal anti-fibronectin | PMID: 39945227 | GeneTex | GTX112794 | polyclonal |
| Mouse monoclonal anti-αSMA | PMID: 36049612 | Sigma-Aldrich | A2547 | 1A4 |
| Rabbit polyclonal anti-RSP11 | PMID: 36049612 | Bethyl Laboratories | A303-936A | polyclonal |
| Mouse monoclonal anti-GAPDH | PMID: 36477534 | Proteintech | 60004-1-Ig | monoclonal |
| Mouse monoclonal anti-PTK2/FAK | PMID: 36626371 | ThermoFisher | MA5-15588 | 10H7 |
| Rabbit polyclonal anti-PCDH7 | PMID: 25494202 | Sigma-Aldrich | HPA046521 | polyclonal |
| Rabbit polyclonal anti-human ALB | PMID: 36049612 | Proteintech | CL2513A | polyclonal |
| BD Pharmingen™ DAPI Solution | PMID: 40311014 | BD biosciences | 564907 | N/A |
| Goat anti-Rabbit IgG (H+L) Secondary Antibody, Alexa Fluor® 594 conjugate | PMID: 39927690 | ThermoFisher | A-11012 | N/A |
| Goat anti-Rabbit IgG (H+L) Secondary Antibody, Alexa Fluor® 488 conjugate | PMID: 39927690 | ThermoFisher | A-11008 | N/A |
| Goat anti-mouse IgG (H+L) Secondary Antibody, Alexa Fluor® 594 conjugate | PMID: 39927690 | ThermoFisher | A-11005 | N/A |
| Goat anti-mouse IgG (H+L) Secondary Antibody, Alexa Fluor® 488 conjugate | PMID: 39927690 | ThermoFisher | A-11001 | N/A |
| Anti-IL10RB neutralizing antibody | PMID: 38829126 | R&D Systems | MAB874 | N/A |

- 1. **Cell lines**

| **Name** | **Citation** | **Supplier** | **Cat no.** | **Passage no.** | **Authentication test method** |
| --- | --- | --- | --- | --- | --- |
| iPSC-9 | PMID: 29249360 | homemade |  | P30-P40 | STR |
| iPSC-W3 | PMID: 29249360 | homemade |  | P30-P40 | STR |
| Primary hepatocytes |  | Lonza | HUCSD | P1 | STR |
| Primary Kupffer cells |  | Lonza | HLKC | P1 | STR |
| Primary hepatic stellate cells |  | Lonza | HUCLS | P3-P5 | STR |

- 1. **Organisms**

| **Name** | **Citation** | **Supplier** | **Strain** | **Sex** | **Age** | **Overall n number** |
| --- | --- | --- | --- | --- | --- | --- |
|  |  |  |  |  |  |  |

- 1. **Sequence based reagents**

| **Name** | **Sequence** | **Supplier** |
| --- | --- | --- |
| Human Primers for qRT-PCR |  | Integrated DNA Technologies |
| COL1A1 | Forward:  GAGGGCCAAGACGAAGACATC  Reverse:  CAGATCACGTCATCGCACAAC | Integrated DNA Technologies |
| ACTA2 | Forward:  AAAAGACAGCTACGTGGGTGA  Reverse:  GCCATGTTCTATCGGGTACTTC | Integrated DNA Technologies |
| FN1 | Forward:  CGGTGGCTGTCAGTCAAAG  Reverse:  AAACCTCGGCTTCCTCCATAA | Integrated DNA Technologies |
| ETS2 | Forward:  CCCCTGTGGCTAACAGTTACA  Reverse:  AGGTAGCTTTTAAGGCTTGACTC | Integrated DNA Technologies |
| GATA6 | Forward:  CTCAGTTCCTACGCTTCGCAT  Reverse:  GTCGAGGTCAGTGAACAGCA | Integrated DNA Technologies |
| IRF1 | Forward:  ATGCCCATCACTCGGATGC  Reverse:  CCCTGCTTTGTATCGGCCTG | Integrated DNA Technologies |
| LRAT | Forward:  CAACTTCACGCTCTTTAGTTCGG  Reverse:  GGCAACACGGTTGTCTCCT | Integrated DNA Technologies |
| PLIN2 | Forward:  ATGGCATCCGTTGCAGTTGAT  Reverse:  GGACATGAGGTCATACGTGGAG | Integrated DNA Technologies |
| GDF2 | Forward:  AGAACGTGAAGGTGGATTTCC  Reverse:  CGCACAATGTTGGACGCTG | Integrated DNA Technologies |
| HGF | Forward:  GCTATCGGGGTAAAGACCTACA  Reverse:  CGTAGCGTACCTCTGGATTGC | Integrated DNA Technologies |
| RSPO3 | Forward:  TGTGCAACATGCTCAGATTACA Reverse:  TGCTTCATGCCAATTCTTTCCA | Integrated DNA Technologies |
| PTK2 | Forward:  GCTTACCTTGACCCCAACTTG  Reverse:  ACGTTCCATACCAGTACCCAG | Integrated DNA Technologies |
| ALDH1A1 | Forward:  GCACGCCAGACTTACCTGTC  Reverse:  CCTCCTCAGTTGCAGGATTAAAG | Integrated DNA Technologies |
| RBP1 | Forward:  TCCAGTCACTCCCCGAAATG  Reverse:  AGGTACTCCTCGAAATTCTCGTT | Integrated DNA Technologies |
| CYP26A1 | Forward:  ATGAAGCGCAGGAAATACGG  Reverse:  AGGAGTCGTGCAGGTTAGAGA | Integrated DNA Technologies |
| PNPLA3 | Forward:  GAAGGCCAGGAGTCGGAAC  Reverse:  TGCCTATTTTGCCGGAGATGA | Integrated DNA Technologies |
| TIMP1 | Forward:  CTTCTGCAATTCCGACCTCGT  Reverse:  ACGCTGGTATAAGGTGGTCTG | Integrated DNA Technologies |
| FASLG | Forward:  TGCCTTGGTAGGATTGGGC  Reverse:  GCTGGTAGACTCTCGGAGTTC | Integrated DNA Technologies |
| ISG15 | Forward:  CTGTTCTGGCTGACCTTCG  Reverse:  GGCTTGAGGCCGTACTCC | Integrated DNA Technologies |
| RSAD2 | Forward:  TGGGTGCTTACACCTGCTG  Reverse:  GAAGTGATAGTTGACGCTGGTT | Integrated DNA Technologies |
| SCD | Forward:  TCTAGCTCCTATACCACCACCA  Reverse:  TCGTCTCCAACTTATCTCCTCC | Integrated DNA Technologies |
| ACLY | Forward:  TCGGCCAAGGCAATTTCAGAG  Reverse:  CGAGCATACTTGAACCGATTCT | Integrated DNA Technologies |
| HMGCR | Forward:  TGATTGACCTTTCCAGAGCAAG  Reverse:  CTAAAATTGCCATTCCACGAGC | Integrated DNA Technologies |
| FDPS | Forward:  TGTGACCGGCAAAATTGGC  Reverse:  GCCCGTTGCAGACACTGAA | Integrated DNA Technologies |
| FDFT1 | Forward:  CCACCCCGAAGAGTTCTACAA  Reverse:  TGCGACTGGTCTGATTGAGATA | Integrated DNA Technologies |
| CCL5 | Forward:  CCAGCAGTCGTCTTTGTCAC  Reverse:  CTCTGGGTTGGCACACACTT | Integrated DNA Technologies |
| GM-CSF | Forward:  TCCTGAACCTGAGTAGAGACAC  Reverse:  TGCTGCTTGTAGTGGCTGG | Integrated DNA Technologies |
| CYP3A4 | Forward:  CCGAGTGGATTTCCTTCAGCTG  Reverse:  TGCTCGTGGTTTCATAGCCAGC | Integrated DNA Technologies |
| TNF | Forward:  CTCTTCTGCCTGCTGCACTTTG  Reverse:  ATGGGCTACAGGCTTGTCACTC | Integrated DNA Technologies |
| IL1B | Forward:  ATGATGGCTTATTACAGTGGCAA  Reverse:  GTCGGAGATTCGTAGCTGGA | Integrated DNA Technologies |
| IL6 | Forward:  ACTCACCTCTTCAGAACGAATTG  Reverse:  CCATCTTTGGAAGGTTCAGGTTG | Integrated DNA Technologies |
| CXCL9 | Forward:  CCAGTAGTGAGAAAGGGTCGC  Reverse:  AGGGCTTGGGGCAAATTGTT | Integrated DNA Technologies |
| CCL1 | Forward:  CTCATTTGCGGAGCAAGAGAT  Reverse:  GCCTCTGAACCCATCCAACTG | Integrated DNA Technologies |
| CCL17 | Forward:  TTCTCTGCAGCACATCCACGCA  Reverse:  CTGGAGCAGTCCTCAGATGTCT | Integrated DNA Technologies |
| CXCL13 | Forward:  GCTTGAGGTGTAGATGTGTCC  Reverse:  CCCACGGGGCAAGATTTGAA | Integrated DNA Technologies |
| PDGFB | Forward:  CTCGATCCGCTCCTTTGATGA  Reverse:  CGTTGGTGCGGTCTATGAG | Integrated DNA Technologies |
| PPARG | Forward:  GGGATCAGCTCCGTGGATCT  Reverse:  TGCACTTTGGTACTCTTGAAGTT | Integrated DNA Technologies |
| GATA4 | Forward:  CGACACCCCAATCTCGATATG  Reverse: GTTGCACAGATAGTGACCCGT | Integrated DNA Technologies |
| IRF2 | Forward:  CATGCGGCTAGACATGGGTG  Reverse:  GCTTTCCTGTATGGATTGCCC | Integrated DNA Technologies |
| RBP1 | Forward:  TCCAGTCACTCCCCGAAATG  Reverse:  AGGTACTCCTCGAAATTCTCGTT | Integrated DNA Technologies |
| SPARCL1 | Forward:  ACGGTAGCACCTGACAACAC  Reverse:  ATGGTGGGAATCGTCTTCTGT | Integrated DNA Technologies |
| SOCS3 | Forward:  CCTGCGCCTCAAGACCTTC  Reverse:  GTCACTGCGCTCCAGTAGAA | Integrated DNA Technologies |
| IL10 | Forward:  GACTTTAAGGGTTACCTGGGTTG  Reverse:  TCACATGCGCCTTGATGTCTG | Integrated DNA Technologies |
| ARG1 | Forward:  GTGGAAACTTGCATGGACAAC  Reverse:  AATCCTGGCACATCGGGAATC | Integrated DNA Technologies |
| MCL1 | Forward:  CCAAGAAAGCTGCATCGAACCAT  Reverse:  CAGCACATTCCTGATGCCACCT | Integrated DNA Technologies |
| CCND1 | Forward:  GCTGCGAAGTGGAAACCATC  Reverse:  CCTCCTTCTGCACACATTTGAA | Integrated DNA Technologies |
| SOCS1 | Forward:  CACGCACTTCCGCACATTC  Reverse:  TAAGGGCGAAAAAGCAGTTCC | Integrated DNA Technologies |
| IL10RB | Forward:  GGAATGGAGTGAGCCTGTCTGT  Reverse:  AAACGCACCACAGCAAGGCGAA | Integrated DNA Technologies |
| shRNA-IL10RB | GCATTCAGACTGGGTAAACAT | Integrated DNA Technologies |

- 1. **Biological samples**

| **Description** | **Source** | **Identifier** |
| --- | --- | --- |
|  |  |  |

- 1. **Deposited data**

| **Name of repository** | **Identifier** | **Link** |
| --- | --- | --- |
| Gene Expression Omnibus | GSE304675 | https://www.ncbi.nlm.nih.gov/geo/query/acc.cgi?acc=GSE304675 |

- 1. **Software**

| **Software name** | **Manufacturer** | **Version** |
| --- | --- | --- |
| GraphPad Prism | GraphPad Software | 8.4.3. |
| ImageJ | NIH | 1.53s |
| R |  | 4.3.1 |
| Seurat |  | R 4.3.1 |
| Tidyverse |  | R 4.3.1 |
| Dplyr |  | R 4.3.1 |
| GGPlot2 |  | R 4.3.1 |
| CellRanger |  | 7.2.0 |
| Bioturing | BioTuring Inc., San Diego, CA | N/A |
| FlowJo | BD Biosciences | 10.8.0 |

- 1. **Other (*e.g*. drugs, proteins, vectors etc.)**

| **Assay kits** |  |  |
| --- | --- | --- |
| Triglyceride assay | Promega | J3160 |
| Human IL1β ELISA | R&D Systems | DY201 |
| Human IL6 ELISA | R&D Systems | DY206 |
| Human TNFα ELISA | R&D Systems | DY210 |
| Human TGFβ1 ELISA | R&D Systems | DY240 |
| Human TGFβ1 ELISA | R&D Systems | DY220 |
| Human HGF ELISA | R&D Systems | DY294 |
| Hydroxyproline assay kit | Millipore Sigma | MAK569 |
| vitamin A kit | CUSABIO | CSB-E07889h |
| Human IL-10 ELISA | R&D Systems | DY217 |
| Human ALB ELISA | R&D Systems | DY1455 |
| MDA colorimetric assay kit | ThermoFisher | EEA015 |
| Cellular ROS assay kit | Invitrogen | D399 |
| Total bile acid assay kit | Cell Biolabs | STA-631 |
| Glycogen-Glo assay | Promega | J5051 |
|  |  |  |
| **Chemicals** |  |  |
| Oil Red O solution | Sigma | O1391-250ML |
|  |  |  |
| **Growth factors** |  |  |
| Animal Free Human BMP-4 | Peprotech | AF-120-05ET |
| Human FGF1 | Peprotech | 100-17A |
| Animal-Free Human EGF | Peprotech | AF-100-15 |
| FGF-Basic (AA 10-155) | Life Technologies | PHG0024 |
| Human Flt3 Ligand | Peprotech | 300-19 |
| Human HGF | Peprotech | 100-39 |
| Human holo-transferrin | Sigma | T4132 |
| Human IL-3 | Peprotech | 200-03 |
| Human IL-6 | Peprotech | 200-06 |
| Human insulin | Sigma | 91077C |
| Human M-CSF | Peprotech | 300-25 |
| Human oncostatin M (OSM) | R&D systems | 295-OM-050 |
| Human PDGF-AB | Peprotech | 100-00AB |
| Human SCF | Peprotech | 300-07 |
| Human VEGF | Peprotech | 100-20 |
| Human PDGF-BB | Peprotech | 500-P47 |
|  |  |  |
| **Supplements and chemicals** |  |  |
| CHIR99021 | Stemcell Technologies | 72054 |
| B-27 | Life Technologies | 0080085-SA |
| B-27 (minus insulin) | Life Technologies | A1895601 |
| CD34+ Expansion Supplement (10X) | Stemcell Technologies | 0269135050-061 |
| GlutaMAX Supplement | Life Technologies | 35050-061 |
| Heparin, sodium salt from porcine instestine | Sigma | H3149 |
| Human AB serum | atlanta biologicals | s40110H |
| Human PB plasma | Stemcell Technologies | 70039 |
| Insulin-Transferrin-Selenium (ITS -G) (100X) | Life Technologies | 41400-045 |
| Insulin-Transferrin-Selenium-X (100X) | Life Technologies | 51500056 |
| JAK inhibitor | EMD millipore | 420099 |
| KnockOut™ Serum Replacement (KOSR) | Life Technologies | 10828028 |
| L-Ascorbic acid | Sigma | A4544 |
| Heparin, sodium salt from porcine instestine | Stemgent | 04-0074 |
| Lipid Mixture 1, Chemically Defined (100X) | Sigma | L0288 |
| Lonza hepatocyte culture medium | Lonza | CC-3198 |
| DMSO | ATCC | X-4 |
| glucose-free DMEM | Life Technologies | 21063029 |
| Palmitic acid | Sigma | P0500 |
| Non-essential amino acid | Life Technologies | 11140050 |
| Retinol | Sigma | R7632-100MG |
| ROCK inhibitor (Y-27632) | Stemcell Technologies | 72308 |
| Glucose | Life Technologies | A2494001 |
| Oleic acid | Sigma | O3008 |
| STEMdiff Definitive Endoderm Kit | Stemcell Technologies | 05110 |
| Dexamethasone | Sigma | D4902 |
| LPS | Santa Cruz Biotechnology | sc-3535 |
|  |  |  |
| **Cell culture reagents** |  |  |
| Accutase | Innovative Cell Technologies | AT 104-500 |
| Basement Membrane Matrix | Corning | 356230 |
| Gentamicin | Life Technologies | 15750078 |
| ReLeSR | Stemcell Technologies | 05872 |
| Mesoderm Induction Medium | Stemcell Technologies | 05221 |
| RPMI/1640 | Life Technologies | 22400071 |
| IMDM | Life Technologies | 12440046 |
| F12 medium | Life Technologies | 11765054 |
| Versene | Life Technologies | 15040066 |
| DMEM/F12 | Life Technologies | 11330032 |
| mTeSR1 | Stemcell Technologies | 85850 |
| mTeSR1 Plus | Stemcell Technologies | 100-0276 |
| Transwell permeable supports | Corning Costar | 3470/3460 |

- 1. **Please provide the details of the corresponding methods author for the manuscript:**

| Dr. Xianfang Wu, Department of Infection Biology, Lerner Research Institute, Cleveland Clinic Foundation, 9500 Euclid Avenue / NE20, Cleveland, OH 44195, United States; Phone: +1-216-445-1458; Fax: +1-216-444-0512; Email: [wux4@ccf.org](mailto:wux4@ccf.org). |
| --- |

**2.0 Please confirm for randomised controlled trials all versions of the clinical protocol are included in the submission. These will be published online as supplementary information.**

| N/A |
| --- |
