# Supplementary material for: Single-cell analysis of heterogeneity in reverted hiPSC-derived human hepatic stellate cells
Source: JHEP Rep. 2025 Nov 10;8(2):101669. doi: 10.1016/j.jhepr.2025.101669 (PMC12803900; doi:10.1016/j.jhepr.2025.101669)
Supplement: Multimedia component 4 [file mmc4.pdf]

Research article

JHEP Reports

Single-cell analysis of heterogeneity in reverted hiPSC-derived human hepatic stellate cells

Authors

Xinjia Wang, Eun Hee Ha, Lu Bian, …, Weibo Chen, Yujie Zhang, Xianfang Wu

Correspondence

wux4@ccf.org (X.F. Wu).

Graphical abstract

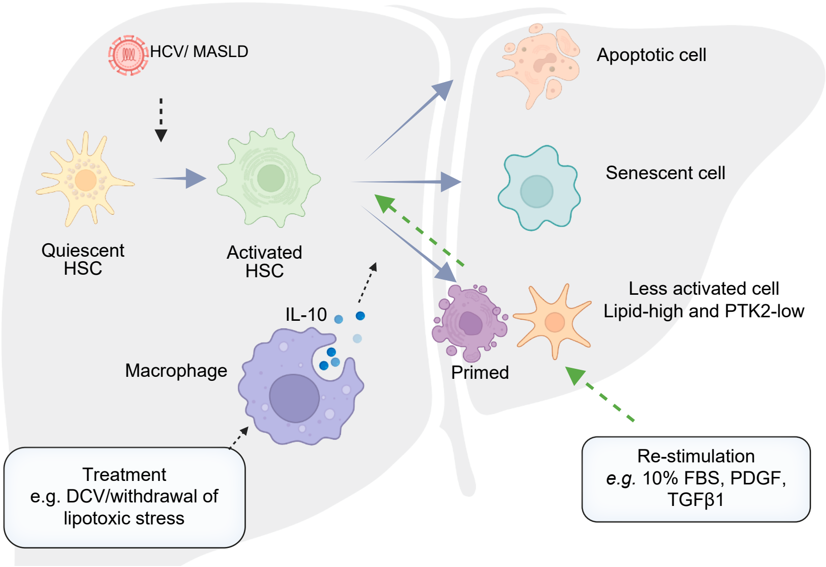

Highlights:

- HSCs revert toward quiescence after HCV cure or lipotoxic withdrawal.
- Reverted HSCs regain vitamin A and quiescent markers but remain primed.
- ScRNA-seq reveals heterogeneity and a lipid-high, PTK2-low rHSC subset.
- Macrophage IL-10 drives reversion via vitamin A metabolism genes (*LRAT* and *RBP1*).

Impact and implications:

Removing the cause of liver injury—curing hepatitis C or withdrawing lipotoxic stress—allows scar-forming liver cells (hepatic stellate cells) to partly revert to a healthier, vitamin-A-storing state; single-cell profiling reveals its heterogeneity and identify a subset nearing true quiescence. This rebound depends on intercellular interaction, in part on the immune signal IL-10 from macrophages, yet reverted cells remain easier to reactivate. These findings provide insights into dynamics of hepatic stellate cells and suggest potential therapeutic avenues for liver fibrosis by targeting stellate cell reversion.

https://doi.org/10.1016/j.jhepr.2025.101669
© 2025 The Author(s). Published by Elsevier B.V. on behalf of European Association for the Study of the Liver (EASL). This is an open access article under the CC BY license (http://creativecommons.org/licenses/by/4.0/).

JHEP|Reports

Single-cell analysis of heterogeneity in reverted hiPSC-derived human hepatic stellate cells

Xinjia Wang[1,†], **Eun Hee Ha**[1,†], Lu Bian[1], Zhuoying Feng[1], Fan Zhang[1], Kyle O'Shaughnessy[1,2], Lei Wang[1], Andrea Hochwald[1], Yifei Zheng[1], Weibo Chen[1], Yujie Zhang[3], Xianfang Wu[1,4,*]

JHEP Reports **2026**. vol. 8 | 1–13

Check for updates

Background & Aims: Activated HSCs are known to drive fibrogenesis, but their fate following injury resolution remains unclear. We aimed to investigate whether human activated HSCs revert to a less activated state, and to characterize features of such reversion using a human induced pluripotent stem cell (hiPSC)-derived multicellular liver model.

Methods: We used a hiPSC-derived liver culture containing hepatocytes, HSCs, and macrophages. HSCs were activated by HCV infection or a lipotoxic milieu modeling metabolic dysfunction-associated steatotic liver disease (MASLD) and subjected to injury resolution through antiviral treatment or replacement with a healthy medium. Reverted HSCs were characterized via gene expression profiling, functional assays, and single-cell RNA sequencing (scRNA-seq). The role of macrophage-derived IL-10 in HSC reversion was investigated through receptor knockdown and cytokine treatment experiments.

Results: Following either HCV clearance or withdrawal of lipotoxic stress, activated HSCs reverted to a less activated state, regaining lipid droplets and vitamin A storage while re-expressing quiescent HSC markers. scRNA-seq revealed heterogeneity among reverted HSCs, identifying subpopulations expressing apoptotic, senescent, or quiescent-like signatures. A distinct lipid-high, PTK2-low population closely resembled naïve quiescent HSCs. Functional assays demonstrated that rHSCs retained partial quiescence but exhibited heightened sensitivity to fibrogenic re-stimulation (n = 4, $p < 0.05$). Mechanistically, macrophage-derived IL-10 promoted HSC reversion by inducing vitamin A metabolism-related genes, including *LRAT* and *RBP1* (n = 4, $p < 0.01$).

Conclusions: Activated human HSCs demonstrate plasticity, reverting to a quiescent-like state following resolution of viral or metabolic injury, although they remain primed for reactivation. Macrophage-derived IL-10 plays a critical role in driving this reversion by regulating vitamin A metabolism. These findings provide insights into HSC dynamics and suggest potential therapeutic avenues for liver fibrosis by targeting HSC reversion.

© 2025 The Author(s). Published by Elsevier B.V. on behalf of European Association for the Study of the Liver (EASL). This is an open access article under the CC BY license (http://creativecommons.org/licenses/by/4.0/).

Introduction

Liver fibrosis, a hallmark of chronic liver disease, arises from the excessive accumulation of extracellular matrix (ECM) components, driven primarily by the activation of hepatic stellate cells (HSCs).[1] In response to persistent injury—such as viral infection, alcohol exposure, or metabolic stress—HSCs transition from a quiescent, vitamin A-storing state to a myofibroblast-like phenotype characterized by enhanced proliferation, contractility, and production of fibrogenic proteins such as collagen type I. This activation process is well established as the central driver of fibrosis progression, ultimately leading to cirrhosis if unchecked. However, the fate of activated HSCs (aHSCs) following the resolution of the underlying injury remains poorly defined,[1] particularly in human systems, limiting the development of therapies aimed at reversing fibrosis.

Evidence from rodent models and clinical observations suggests that liver fibrosis can regress when the causative insult is removed,[2–4] with activated HSCs undergoing apoptosis,[5,6] senescence,[1,7] or reversion to a less activated, quiescent-like state.[8,9] In mice, reverted HSCs regain some quiescent features, such as lipid-droplet storage, but retain a 'primed' phenotype, distinct from naïve quiescent HSCs, indicating incomplete restoration.[8,9] Although these findings highlight the plasticity of HSCs, their relevance to human liver fibrosis remains uncertain because of the species-specific differences and the lack of robust human-relevant models. Understanding whether human HSCs can revert to a functional, quiescent-like state—and the mechanisms governing this process—is critical for harnessing this plasticity to mitigate fibrosis.

To address this gap, we utilized a human induced pluripotent stem cell (hiPSC)-derived multicellular liver culture system,

* Corresponding author. Address: Department of Infection Biology, Lerner Research Institute, Cleveland Clinic, 9500 Euclid Avenue/NE20, Cleveland, OH 44195, USA. Tel.: +1 216 445 1458.
E-mail address: wux4@ccf.org (X.F. Wu).
† These authors contributed equally.
https://doi.org/10.1016/j.jhepr.2025.101669

previously developed by our laboratory, which integrates hiPSC-derived hepatocytes, HSCs, and macrophages to mimic the human liver microenvironment.[10] This platform enables the study of HSC dynamics in a physiologically relevant context. In this study, we investigated the fate of activated human HSCs following HCV infection and metabolic stress and its subsequent 'cure', characterizing their reversion potential and sensitivity to re-stimulation with both familiar and novel fibrogenic stimuli. Additionally, we explored the role of co-cultured macrophages in modulating HSC reversion, focusing on key molecular mediators. Our findings reveal that human HSCs exhibit significant reversion capacity, mediated in part by macrophage-derived IL-10, yet remain functionally distinct from naïve quiescent HSCs (qHSCs), offering new insights into HSC plasticity and potential therapeutic targets for fibrosis resolution.

Materials and methods

Single-cell RNA sequencing

Single-cell RNA sequencing (scRNA-seq) was performed at the Genomic Core of Rockefeller University using the 10 × Genomics Chromium platform following the manufacturer's protocol. Briefly, single-cell suspensions were prepared and adjusted to the desired concentration to achieve optimal cell capture efficiency (~10,000 cells per sample). Sequencing was performed on an Illumina NovaSeq 6000 platform using paired-end sequencing to achieve a depth of approximately 50,000 reads per cell.

The returned Fastq files of DMSO and DCV samples (DMSO-1, DMSO-2, DCV-1, and DCV-2) were analyzed with Cell Ranger and converted to Seurat objects by running the corresponding scRNA-seq codes provided by the Cleveland Clinic Center for Immunotherapy and Precision Immuno-Oncology (CITI) on a High Performance Computing (HPC) platform. The final integrated Seurat object generated from the four Fastq files was uploaded to BioTuring. All subsequent analyses and graphs (*e.g.* heatmap, differential gene analysis, gene ontology (GO) enrichment analysis, Umap, t-SNE, and violin plots) were generated with the BBrowserX software (BioTuring Inc., San Diego, CA, USA).

Statistical analysis

Detailed information regarding all statistical tests, the specific value of 'n' and its representation (*e.g.* number of cell clones or experimental replicates) can be found in the respective figure legends. Graphs depict results presented as means ± SD. Group comparisons were conducted using the unpaired *t* test with Welch's correction or one-way ANOVA/Tukey's *post-hoc* test, as appropriate, to calculate precise *p* values unless otherwise specified. Statistical analyses were carried out using Graph Pad PRISM 10 (GraphPad Software, San Diego, CA, USA). All *p* values are reported, and a $p > 0.05$ denotes statistical non-significance and is marked as gray.

Results

Establishment of a human-relevant *in vitro* system to study HSC activation and reversion

In our recent study modeling fatty liver disease, we developed a multicellular *in vitro* culture system by co-culturing hiPSC-derived hepatocytes, HSCs, and macrophages at an 8:1:1 ratio, closely reflecting the cellular composition of a healthy liver.[10] We termed this system the 'multicellular liver culture'. In this system, the co-cultured HSCs remain in a quiescent-like state, characterized by low expression levels of activation markers, while maintaining high levels of quiescence-related markers. Importantly, in response to various stimuli, these HSCs become activated and transdifferentiate into a myofibroblast-like phenotype, exhibiting high expression of activation markers. These features make the multicellular liver culture an ideal platform for investigating HSC activation and potentially the reversion of aHSCs.

To test this possibility, we infected liver cultures with HCV (Fig. S1A), a virus well known for its ability to induce stellate cell activation in patients with this infection.[11] We first characterized hepatocyte responses: progressively-increasing interferon-stimulated genes, lipid-droplet accumulation with upregulation of lipogenic enzymes, elevated triglyceride and cholesterol, and increased lipid-metabolism genes. Reactive oxygen species (ROS) and malondialdehyde (MDA) were also elevated, indicating oxidative stress and lipid peroxidation (Fig. S1A–K). In parallel, HSCs from HCV-infected cultures showed higher activation markers than HSCs from uninfected cultures and displayed increased cytokine transcripts (Fig. S1L–N). To define the role of macrophages, we compared cultures with and without them: HSC activation was markedly reduced without macrophages. In infected cultures, macrophages shifted from an M2-like (anti-inflammatory) to an M1-like (pro-inflammatory) phenotype, secreting interferon-β, transforming growth factor beta 1 (TGF-β1), and IL-1β, and upregulating *TNF* and *PDGFB* (Fig. S2A–F). Because neither HSCs nor macrophages are permissive to HCV,[12] these data indicate that infection-induced responses in hepatocytes—and the secondary inflammatory program in macrophages—drive HSC activation.

We next examined whether HCV cure reverses HSC activation. Liver cultures were infected with HCV for 1 week before being treated with either daclatasvir (DCV), an HCV nonstructural protein 5A (NS5A) inhibitor, or vehicle (Fig. 1A). As expected, DCV effectively cleared HCV infection (Fig. 1A and B), which by Day 12 reduced to background levels. This trend was accompanied by gradually decreasing levels of inflammatory cytokines (Fig. 1C). Importantly, DCV treatment had no noticeable effects on hepatic marker expression or functions (Fig. S1O–Q).

In vehicle-treated cultures, HSC activation markers continued to rise, whereas in DCV-treated cultures they peaked at Day 0 and then decreased significantly, reaching very low levels comparable to qHSCs by Day 12 (Fig. 1D). These findings indicate that HCV infection clearly induces HSC activation, while curing HCV reduces activation, suggesting a possible HSC reversion. This is further supported by the progressive decline in cytokines known to promote HSC activation (Fig. 1E), reinforcing the notion that HCV cure likely induces HSC reversion.

Detailed characterization of reverted HSCs

The above findings suggest that following HCV cure, aHSCs undergo reversion to a less activated state. Although protein levels of activation markers return to near-quiescent levels,

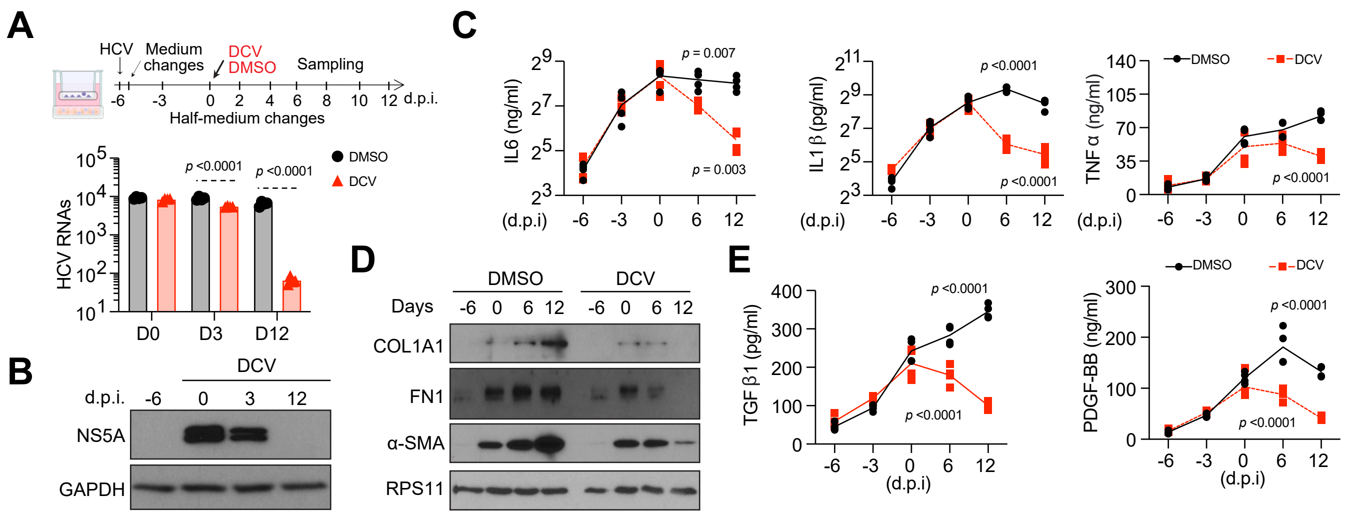

Fig. 1. Establishment of a human-relevant *in vitro* system to study HSC activation and reversion. Top: Schematic representation of HCV infection, DCV treatment, and sample collection. Bottom: HCV RNA quantification by qRT-PCR (n = 4, mean ± SD, ANOVA). (B) Western blot analysis of HCV nonstructural protein 5A (NS5A) and housekeeping protein GAPDH in purified hepatocytes. (C, E) ELISA analysis of selected cytokines in cell culture supernatants (n = 4, mean ± SD, ANOVA). (D) Western blot analysis of activation markers and housekeeping protein RPS11 in purified HSCs. α-SMA, alpha-smooth muscle actin; COL1A1, collagen type I alpha 1 chain; DCV, daclatasvir; FN1, fibronectin; GAPDH, glyceraldehyde 3-phosphate dehydrogenase; HSCs, hepatic stellate cells; PDGFB, platelet derived growth factor subunit B; qRT-PCR, quantitative real time-PCR; TGFβ1, transforming growth factor beta 1; TNFα, tumor necrosis factor-alpha; ALDH1A1, aldehyde Dehydrogenase 1A1; LRAT, lecithin-retinol acyltransferase; CYP26A1, cytochrome P450 26A1; PNPLA3, patatin-like phospholipase domain-containing protein 3; HGF, hepatocyte growth factor.

their transcript levels remain elevated except *FN1* (Fig. 2A). This indicates that reverted human HSCs are transcriptionally—and likely functionally—distinct from qHSCs, reflecting a partial but incomplete reversion to the quiescent state, consistent with findings from mouse models.[8,9]

To further characterize these reverted HSCs (rHSCs), we first compared their expression of key genes important for HSC quiescence[13] and homeostatic and hepatoprotective functions[14–18] with those in naïve qHSCs. Many genes were comparable to those observed in qHSCs (Fig. 2B and S3A), however, some exhibited either no restoration or incomplete restoration (Fig. 2B and S3A).

Next, we performed functional characterizations to compare rHSCs with quiescent and activated HSCs. Compared with aHSCs, rHSCs exhibited higher levels of vitamin A, although these levels remained significantly lower than those observed in qHSCs (Fig. 2C). They also showed higher ROS levels than qHSCs but slightly lower than aHSCs (Fig. 2D). Similarly, rHSCs displayed intermediate levels of proliferation and migration between qHSCs and aHSCs (Fig. 2E and F).

In addition to these bulk analyses, we performed a microscopic assessment of rHSCs at single-cell resolution. We stained lipid droplets, where vitamin A is stored, to evaluate heterogeneity within the rHSC population (Fig. 2G). Consistent with the bulk vitamin A quantification, Oil-red staining generally followed the same trend (Fig. 2H). However, when examining individual cells, we observed clear heterogeneity in red signal intensity within the rHSC population. Some cells displayed red signal levels comparable to qHSCs, while others resembled aHSCs (Fig. 2G). This is further supported by quantification of intracellular lipid using flow cytometry analysis (Fig. S3B). By contrast, before DCV treatment, the majority of HSCs display collagen type I alpha 1 chain (COL1A1)$^+$ and alpha-smooth muscle actin (α-SMA)$^+$ in immunostaining. Flow cytometry for α-SMA similarly showed a highly uniform positive population, indicating limited heterogeneity at baseline (Fig. S3C and D). These findings indicate that rHSCs undergo partial restoration of quiescent characteristics yet retain functional properties that are intermediate between quiescent and activated states.

Finally, we assessed the paracrine effects of rHSCs on co-cultured hepatocytes and macrophages. Following DCV treatment, *HGF* transcripts increased in rHSCs (Fig. 2B), partially restoring culture-wide HGF levels (Fig. 2I). Accordingly, hepatocytes and macrophages exhibited upregulation of canonical HGF-MET proto-oncogene, receptor tyrosine kinase (MET) target genes (Fig. S3E and F).

Single-cell RNA sequencing analysis to compare activated and reverted HSCs

Given the heterogeneous features of rHSCs, we performed single-cell RNA sequencing (scRNA-seq) to further investigate their molecular characteristics. As shown in Fig. S4A, at 12 days post DCV treatment, we prepared two control liver cultures treated with DMSO and two experimental liver cultures treated with DCV for scRNA-seq analysis. Across the four groups, a total of 15,218 individual HSCs were analyzed, with an average sequencing depth of 39,473 reads per cell (Fig. S4A).

Global transcriptomic analysis revealed distinct differences between DMSO and DCV groups (Fig. 3A). To evaluate the global and local clustering patterns, we generated Uniform Manifold Approximation and Projection (Umap) and *t*-distributed Stochastic Neighbor Embedding (t-SNE) plots. The Umap plot showed that DMSO- and DCV-treated cells clustered together in one large region (Fig. S4B), indicating a high degree of similarity in the global expression pattern between the two groups.

Distinct cluster separations between DMSO and DCV groups were observed in the t-SNE plots (Fig. 3B), indicating local differences between the two groups. We also identified a

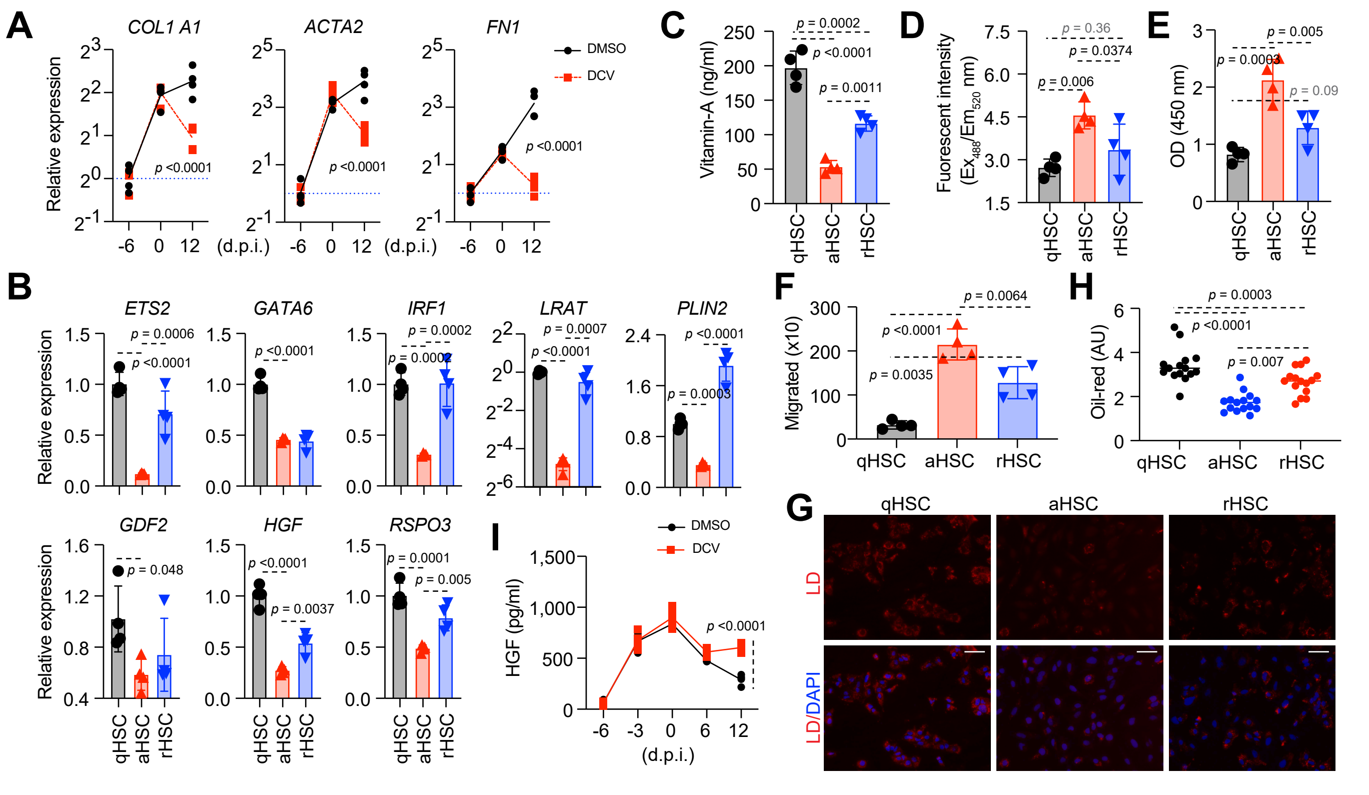

Fig. 2. Detailed characterization of reverted HSCs. In the experiments described in Fig. 1A, qHSCs were isolated from uninfected liver cultures; aHSCs and rHSCs were from infected liver cultures treated with DMSO or DCV (10 nM). (A) Transcript analysis of activation marker by qRT-PCR (n = 4, mean ± SD, ANOVA). (B) Transcript analysis of selected genes in qHSC, aHSC, and rHSC by qRT-PCR (n =, mean ± SD, ANOVA). Purified qHSC, aHSC, and rHSC were quantified for vitamin A by ELISA (C), reactive oxygen species (ROS) by DCFH-DA fluorescence assay (D), BrdU incorporation assay (E), and PDGF-BB-induced cell migration assay (F) (n = 4, mean ± SD, ANOVA). (G-H) Oil-red signals of purified qHSC, aHSC, and rHSC quantified using ImageJ (1.52k) (Scale bars, 100 μm, ANOVA). (I) ELISA analysis of HGF in cell culture supernatants (n = 4, mean ± SD, ANOVA). aHSCs: activated hepatic stellate cells; COL1A1, collagen type I alpha 1 chain; DCV, daclatasvir; ETS1/ETS2, ETS proto-oncogene 1/ETS proto-oncogene 2; FN1, fibronectin; GATA6, GATA binding protein 6; GDF2/BMP9, growth differentiation factor 2; HGF, hepatocyte growth factor; IRF1/IRF2, interferon regulatory factor 1/2; LRAT, lecithin-retinol acyltransferase; PDGFB, platelet derived growth factor subunit B; PLIN2, perilipin 2; qHSCs: quiescent hepatic stellate cells; qRT-PCR, quantitative real time-PCR; rHSCs, reverted hepatic stellate cells; RSPO3, R-spondin 3.

small mixed cluster at the bottom right side of the t-SNE plot, comprising both DMSO- and DCV-treated cells (Fig. S4C) as a hepatocyte contaminant resulting from HSC purification from liver cultures (Fig. S4D).

Using Louvain clustering, the DMSO group was subdivided into five distinct clusters (Fig. S4E). However, transcriptomic analysis focusing on HSC activation and quiescence markers revealed that these clusters expressed comparable levels of activation markers, while showing consistently low expression of quiescent markers (Fig. S4F). To further compare HSC status between the treatment groups, we selected a panel of well-established activation markers and assessed their expression levels using t-SNE visualization (Fig. 3C). For more quantitative analysis, we generated violin plots for each activation marker individually (Fig. 3D). The analysis revealed that all activation markers exhibited higher expression levels in the DMSO compared with the DCV group.

In contrast, HSC quiescent markers were expressed at higher levels in the DCV compared with the DMSO group (Fig. 3E and F), indicating a more quiescent-like phenotypes. These results were further supported by GO analysis, which demonstrated that the DMSO group exhibited significantly higher enrichment for GO terms associated with HSC activation and associated functionalities (Fig. S4G).

Finally, to further validate our findings, we compared our data with a published human liver scRNA-seq dataset.[19] Reverted hiPSC-derived HSCs (rHSCs; DCV group) clustered closer to patient quiescent HSCs (pHSCs) than hiPSC-derived activated HSCs (aHSCs; DMSO group), as evidenced by shorter distances in t-SNE space and concordant expression patterns in gene sets related to collagen regulation (Fig. S5A and B). Consistently, rHSCs in the DCV group display quiescence markers at levels comparable to pHSCs and clearly higher than aHSCs, whereas activation markers are markedly lower in both rHSCs and pHSCs relative to aHSCs (Fig. S5C–F).

These results collectively confirm the effectiveness of DCV treatment in reverting activated HSCs, as evidenced by the downregulation of activation markers and upregulation of quiescent markers.

Single-cell RNA sequencing analysis of reverted HSCs

Next, we used the same Louvain clustering to divide the DCV group into three distinct clusters (Fig. 4A and B). Global transcriptomic analysis revealed that all three DCV clusters exhibited distinct gene expression profiles compared with the DMSO group (Fig. 4C). Notably, clusters 1 and 3 shared a

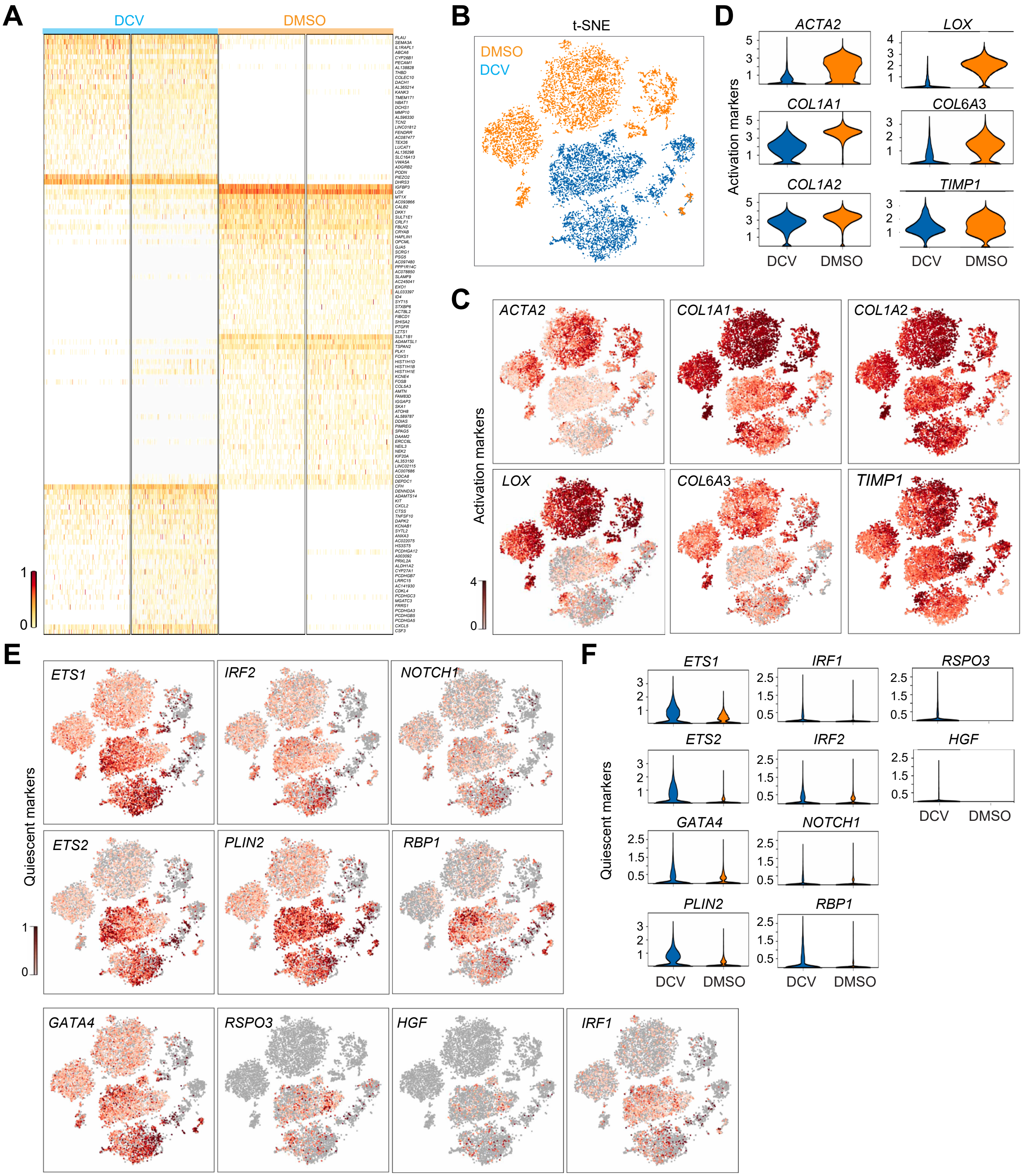

Fig. 3. Single-cell RNA sequencing analysis to compare activated and reverted HSCs. (A) Heatmap comparing differentially expressed genes between DMSO- and DCV-treated HSC populations with (p <0.05) and (log_2 fold change >0). The maximum number of marker genes (30) was selected per group. (B) *t*-SNE visualization of DMSO- and DCV-treated cells. *t*-SNE and violin plots were generated based on individual activation markers (C,D), quiescent and homeostatic markers (E,F). All scRNA-seq analyses were generated with the BBrowserX software (BioTuring Inc., San Diego, CA, USA). COL1A1/COL1A2/COL6A3, collagen type I alpha 1 chain/alpha 2 chain/collagen type VI alpha 3; DCV, daclatasvir; ETS1/ETS2, ETS proto-oncogene 1/ETS proto-oncogene 2; FN1, fibronectin; GATA4, GATA binding protein 4; HGF, hepatocyte growth factor; HSCs, hepatic stellate cells; IRF1/IRF2, interferon regulatory factor 1/2; NOTCH, Notch receptor 1; PLIN2, perilipin 2; RBP1, retinol-binding protein 1; RSPO3, R-spondin 3; scRNA-seq, single-cell RNA sequencing; *t*-SNE, *t*-distributed Stochastic Neighbor Embedding.

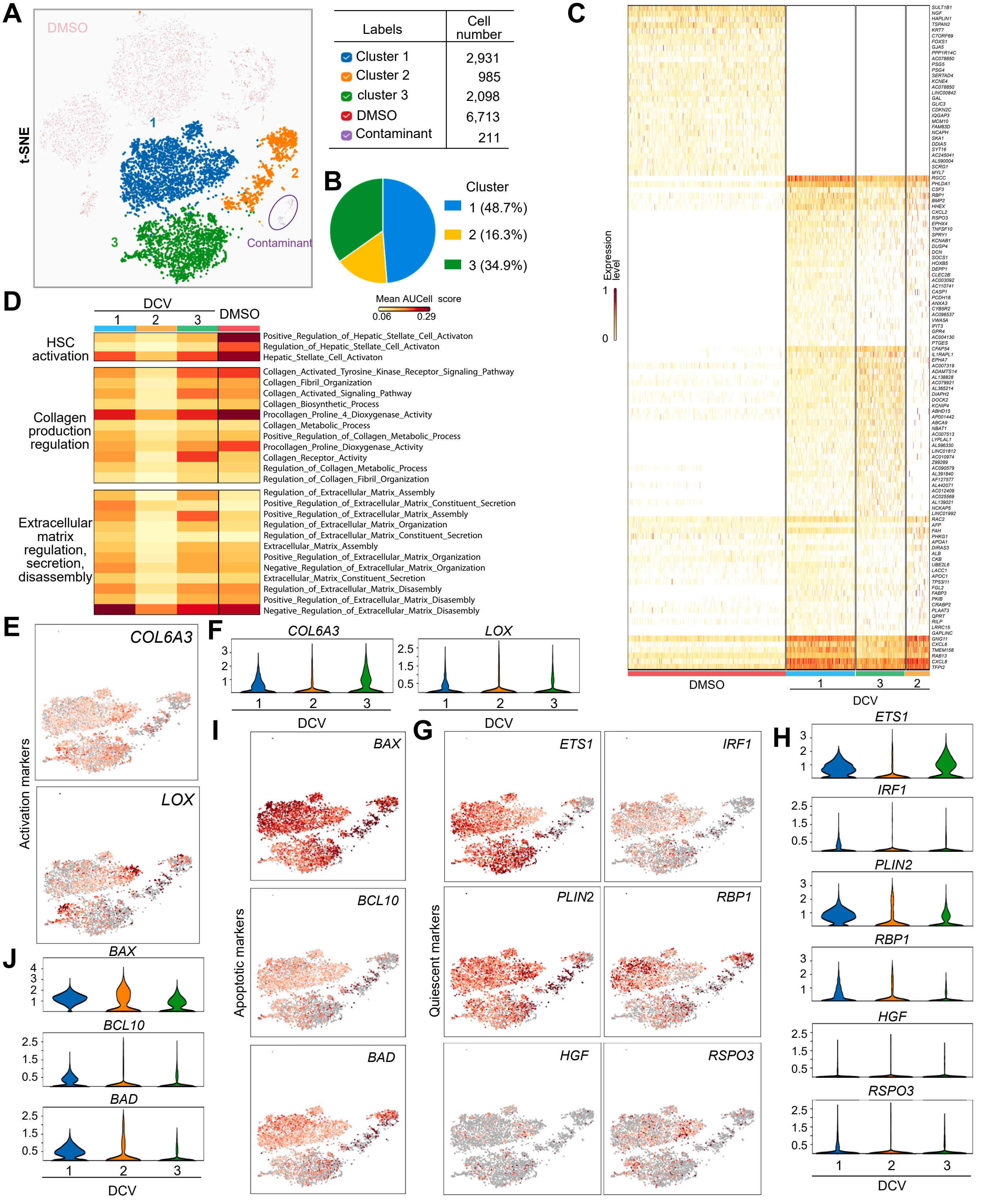

Fig. 4. Single-cell RNA sequencing analysis of reverted HSCs. (A) t-SNE plot with three rHSC subclusters, grouped via the Louvain clustering method (resolution = 0.1). (B) Pie chart of the rHSC subclusters. (C) Heatmap of differentially expressed genes between DMSO and rHSC subclusters. (D) GO enrichment test comparing DMSO to rHSC subclusters. t-SNE and violin plots demonstrating relative expression levels of activation markers (E,F), quiescence-associated markers (G,H), and apoptotic markers (I,J). COL1A1/COL1A2/COL6A3, collagen type I alpha 1 chain/alpha 2 chain/collagen type VI alpha 3; DCV, daclatasvir; ETS1/ETS2, ETS proto-oncogene 1/ETS proto-oncogene 2; GO, Gene Ontology; HGF, hepatocyte growth factor; HSCs, hepatic stellate cells; LOX, lysyl oxidase; PLIN2, perilipin 2; RBP1, retinol-binding protein 1; rHSCs, reverted hepatic stellate cells; RSPO3, R-spondin 3; *t*-SNE, *t*-distributed Stochastic Neighbor Embedding.

higher number of commonly expressed genes compared with cluster 2 (Fig. 4C and S6A). This trend was further supported by GO analysis, indicating a greater similarity between clusters 1 and 3 (Fig. 4D). Further examination of the GO plot revealed that cluster 2 had the lowest expression levels of genes associated with HSC activation or activation-related processes (Fig. 4D). These analyses suggest that cluster 2 represents the least activated and likely the most reverted HSC group among the three DCV clusters.

In rodent models of fibrosis, aHSCs have been shown to undergo apoptosis, senescence, or a less activated, quiescent-like state.[8,9] Based on this paradigm, we focused on evaluating HSC quiescent and activation markers, as well as reversion-associated apoptotic and senescent markers.[1] Analysis of activation markers revealed that cluster 2 contained fewer cells expressing these markers compared with clusters 1 and 3 (Fig. 4E,F and S6B,C), consistent with the GO analysis (Fig. 4D).

When analyzing quiescent markers, we observed mixed outcomes. Some quiescent markers showed higher expression levels in cluster 2 compared with the other clusters, while others exhibited relatively low expression in cluster 2 (Fig. 4G,H and S6D,E). Further analysis of HSC reversion-associated markers, including apoptotic, senescent genes, and senescence-associated secretory phenotype (SASP) genes, revealed substantial heterogeneity across the three clusters (Figs. 4I,J and S6F–K).

Overall, these scRNA-seq analyses highlight the remarkable heterogeneity of rHSCs, revealing that the reversion process does not uniformly restore quiescence. Instead, rHSCs encompass a spectrum of cellular states, likely reflecting varying degrees of activation, quiescence, apoptosis, and senescence.

Identification of a small population within rHSCs resembling quiescent HSCs

As cluster 2 exhibited the lowest levels of activation markers, we focused on cluster 2 for further analysis. We utilized Louvain clustering to further subdivide cluster 2 into two distinct subclusters, named cluster 2.1 and cluster 2.2 (Fig. 5A). Global transcriptomic analysis revealed that cluster 2.1 displayed distinct gene expression profiles compared with the other clusters (Fig. S7A). To gain further insights, we focused on genes associated with HSC activation and quiescence, which revealed a similarly distinct expression profile in cluster 2.1 (Fig. S7B). Cluster 2.1 exhibited higher levels of quiescent markers and lower levels of activation markers, suggesting that cluster 2.1 may represent a more quiescent-like cell population. This was further supported by a direct comparison between cluster 2.1 and the remaining cells in the DCV group (Rest DCV) (Fig. 5B). Additionally, GO analysis revealed a distinct gene expression profile in cluster 2.1, characterized by the lowest expression of genes associated with HSC activation and ECM regulation (Fig. 5C).

To identify cellular markers that could distinguish cluster 2.1 from the rest of the DCV group, we performed a deeper transcriptomic comparison between these two populations. This analysis identified *PTK2* gene as a suitable marker to differentiate cluster 2.1 from other cells (Fig. 5D and E). PTK2 is expressed at significantly higher levels in aHSCs than in qHSCs (Fig. S7C and D).

Because PTK2 is not a cell-surface protein, it cannot be used directly for cell purification. We therefore examined its relationship to intracellular lipid, a hallmark of qHSCs, within the DCV population. Flow cytometry revealed a ~10% of lipid-high, PTK2-low subset (Fig. S7E), closely matching the proportion of cluster 2.1 observed by single-cell analysis with the DCV group (Fig. 5A). We accordingly sorted the top ~10% lipid-high cells and operationally defined them as the C2.1 fraction for downstream analyses. Comparing PTK2 expression between the C2.1 fraction and the Rest DCV population revealed significantly lower PTK2 at both the transcript and protein levels in C2.1, further validating the isolation strategy (Fig. 5F,G and S7F).

Next, we performed functional characterizations to compare C2.1 with the Rest DCV cells. C2.1 exhibited collagen levels comparable to qHSCs (Fig. 5H) and higher vitamin A levels compared with the Rest DCV cells, with levels approaching those of qHSCs (Fig. 5I).

We further compared genes involved in vitamin A metabolism and storage between the two populations.[20] Consistent with the analysis shown in Fig. 5B, both populations expressed *RBP1* and *PLIN2* at relatively higher levels compared with qHSCs. Notably, other genes were also expressed at higher levels in C2.1, making their expression profiles closer to qHSCs (Fig. 5J and S7G).

Together, these findings indicate that C2.1 exhibits features more closely resembling qHSCs, particularly with respect to collagen levels, vitamin A content, and the gene expression profiles related to vitamin A metabolism and storage.

rHSCs are more sensitive to re-stimulation

Seeing similarities between C2.1 and naïve qHSCs, we next asked what distinguishes rHSCs from naïve qHSCs. A key factor was their sensitivity to re-stimulation. In mouse carbon tetrachloride (CCl_4)-induced fibrosis models, reverted HSCs respond more strongly to fibrogenic signals than naïve qHSCs.[8,9] To determine whether this heightened sensitivity also occurs in human rHSCs, we compared the responsiveness of human rHSCs and qHSCs.

As shown in Fig. 6A, rHSCs were isolated from liver cultures infected with HCV and then cured with DCV, while control qHSCs came from cultures exposed to HCV in the presence of DCV for the same duration. Both HSC types were plated on Matrigel-coated dishes and treated with supernatant from HCV-infected cultures to assess activation. Consistent with findings from mouse models,[8,9] human rHSCs—despite expressing activation markers at levels comparable to qHSCs—displayed a heightened response, as evidenced by increased transcript and protein levels of activation markers (Fig. 6B and C). To investigate whether this enhanced sensitivity is specific to stimuli encountered previously, we conducted similar experiments using various new stimuli. Remarkably, rHSCs also exhibited a stronger response to these novel stimuli (Fig. 6D).

These results suggest that human rHSCs, similar to their mouse counterparts, exhibit an intrinsically heightened sensitivity to both previously encountered and novel fibrogenic stimuli, highlighting the persistent priming effect of prior activation.

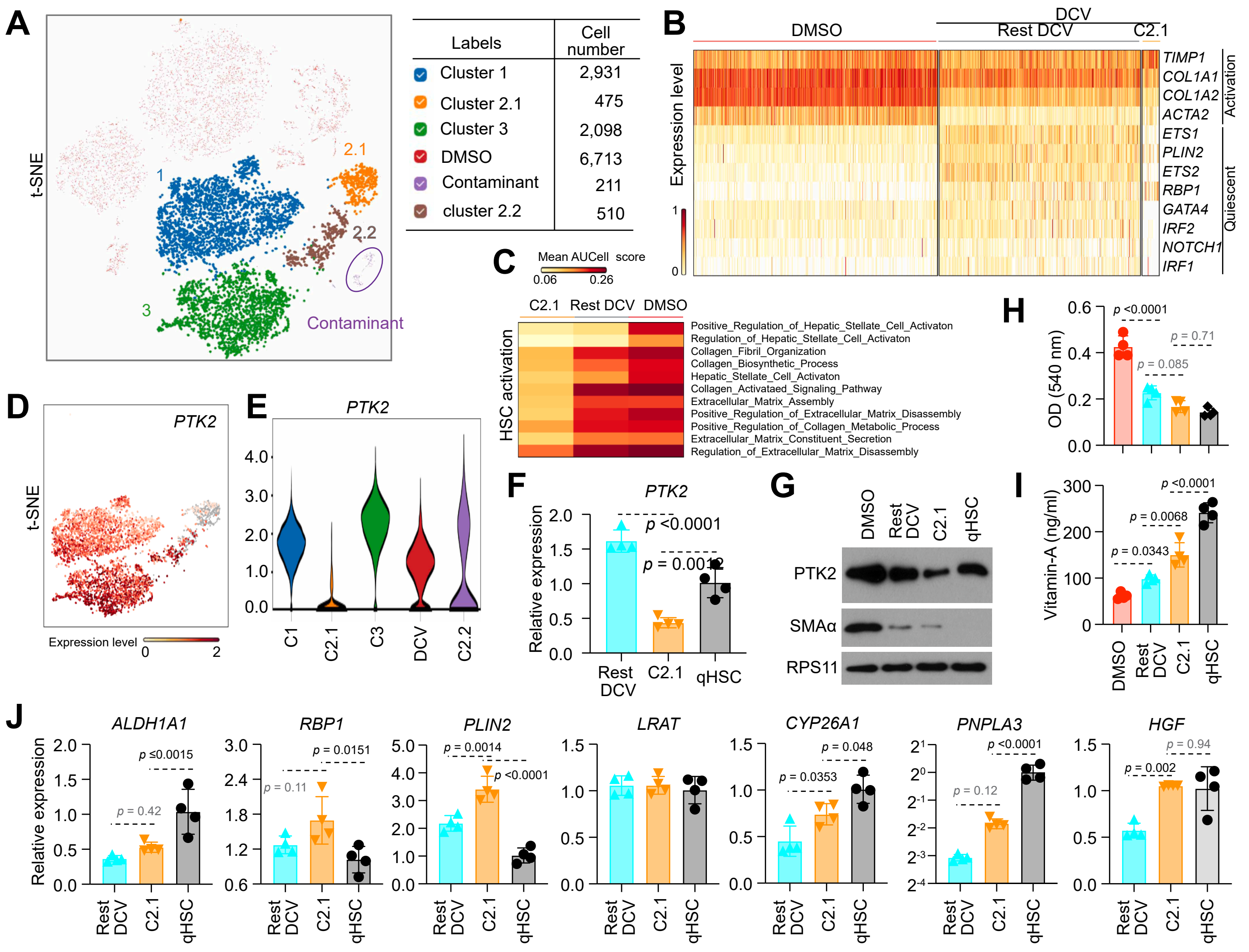

Fig. 5. Identification of a small population within rHSCs resembling quiescent HSCs. (A) t-SNE plot of subcluster 2.1 and 2.2. (B) Heatmap of HSC activation and quiescent markers. (C) GO enrichment test between DMSO, cluster 2.1 (C2.1), and the remaining DCV population (Rest DCV). t-SNE plot (D) or violin plot (E) of expression levels of PTK2 in different DCV clusters. PTK2 in indicated cell groups was analyzed by qRT-PCR (F, n = 4, mean ± SD, ANOVA) and Western blot (G). Analysis of collagen levels (H), and intracellular vitamin A levels (I) in indicated cell groups by hydroxyproline assay and ELISA, respectively (n = 4, mean ± SD, ANOVA). (J) Transcript levels of selected genes related to vitamin A metabolism and storage by qRT-PCR (n = 4, mean ± SD, ANOVA). α-SMA/ACTA2, alpha-smooth muscle actin; COL1A1/COL1A2/COL6A3, collagen type I alpha 1 chain/alpha 2 chain/collagen type VI alpha 3; DCV, daclatasvir; ETS1/ETS2, ETS proto-oncogene 1/ETS proto-oncogene 2; GATA4, GATA binding protein 4; GO, Gene Ontology; HSCs, hepatic stellate cells; IRF1/IRF2, interferon regulatory factor 1/2; NOTCH, Notch receptor 1; PLIN2, perilipin 2; PTK2, protein tyrosine kinase 2; qHSCs, quiescent hepatic stellate cells; qRT-PCR, quantitative real time-PCR; Rest DCV, remaining cells in the DCV group; RBP1, retinol-binding protein 1; rHSCs, reverted hepatic stellate cells; *t*-SNE, *t*-distributed Stochastic Neighbor Embedding.

Critical roles of macrophages in HSC reversion

Given the complexity and heterogeneity of rHSCs, we sought to identify key factors that drive their reversion across the full DCV population. Because HSCs constantly interact with neighboring cells in the liver, we examined whether these interactions influence HSC reversion. In mouse CCl_4-induced fibrosis models, depleting Kupffer cells delays ECM degradation and impairs HSC reversion, indicating their active role in fibrosis resolution.[21] However, whether human macrophages/Kupffer cells similarly promoter HSC reversion following the removal of the causative agents remains unclear.

To test this, we treated multicellular liver cultures with conditioned supernatant from HCV-infected cultures. DCV was included throughout to eliminate any residual infectivity, ensuring the supernatant served only as an activating stimulus for HSCs rather than causing new infection. On Day 6, the supernatant was replaced with basal medium to mimic a rapid 'HCV cure', under two conditions: macrophages retained or macrophages depleted. Twelve days after macrophage manipulation, HSCs were purified and designated as rHSCs (macrophages retained) and rmHSCs (reverted HSCs after macrophage depletion) (Fig. 7A). We analyzed the expression of markers related to HSC quiescence and homeostatic functions, and found that macrophage removal markedly impaired the restoration of these markers (Fig. 7B and S8A), indicating a failure of HSC reversion. This was further supported by vitamin A quantification, which showed that rmHSCs exhibited comparable vitamin A levels to aHSCs (Fig. 7C).

Kupffer cell polarization plays a crucial role in regulating HSC activation and deactivation.[22] Studies show that M2-polarized Kupffer cells secrete IL-10, which can trigger apoptosis in M1 cells and help maintain the M1/M2 balance.[23]

This balance is critical for controlling liver inflammation and fibrosis. However, it is still unclear whether Kupffer cell-derived IL-10 directly promotes the reversion of activated HSCs to a quiescent state.

Following 'HCV cure' (Fig. 7A), we observed a steady increase in IL-10 secretion within the liver cultures, with macrophages as the predominant source (Fig. 7D and S8B). Using lentivirus-based shRNA, we downregulated IL-10 receptor (*IL10*RB) in HSCs within liver cultures (Fig. S8C). Vitamin A quantification revealed that blocking IL-10 signaling in HSCs impaired the reversion of aHSCs, resulting in reduced vitamin A levels (Fig. 7E). A similar inhibitory effect was observed with an IL-10 neutralizing antibody (Fig. 7E and S8D). Conversely, the addition of exogenous IL-10 to liver cultures slightly increased vitamin A levels (Fig. 7F).

To assess physiological relevance of this finding, we analyzed published liver transcriptomic datasets spanning patients with HCV infection with and without antiviral therapy and cohorts with metabolic dysfunction-associated steatotic liver disease (MASLD) and alcohol-associated liver disease (ALD).[24–26] Hepatic *IL10* transcripts increased after anti-HCV treatment and declined with disease progression in MASLD and ALD (Fig. S8E), consistent with a possible role for IL-10 in promoting HSC reversion.

Finally, to elucidate the mechanism, we treated hiPSC-derived aHSCs with IL-10 and found that several genes related to vitamin A metabolism and storage were upregulated in response to IL-10 (Fig. 7G). Under the same conditions, IL-10 treatment downregulated the transcript levels of activation markers, while upregulating apoptotic markers (Fig.7H and I). Activated primary human HSCs showed similar IL-10 responses, albeit with donor-to-donor variability (Fig. S8F–H).

Together, these findings suggest that Kupffer cell-derived IL-10 plays a direct role in promoting HSC reversion, likely by inducing the expression of vitamin A metabolism-related genes and modulating the expression of activation and apoptotic markers.

Reversion of MASLD-activated HSCs

Beyond HCV infection, multiple conditions can trigger HSC activation and liver fibrosis—notably MASLD. In our multicellular liver model, a lipotoxic milieu elicited MASLD-like phenotypes, including robust HSC activation.[10] To test whether 'treating' this MASLD-like state promotes HSC reversion, we replaced the lipotoxic milieu with a healthy medium and performed the same analyses described above to assess reversion (Fig. 8A).

Following healthy medium replacement, activation markers in HSCs from the MASLD model (rmHSC) declined progressively, reaching levels comparable to naïve qHSCs by Day 12 (Fig. 8A and B). This reversion was accompanied by decreases in profibrotic cytokines (Fig. 8C), partial-to-complete restoration of quiescence-associated markers (Fig. 8D and S9A), and upregulation of canonical HGF-MET target genes in hepatocytes and macrophages (Fig. S9B and C). rmHSC also displayed heterogeneity in intracellular lipid content (Fig. 8E and S9D,E) and showed normalization toward qHSC baselines for intracellular lipids, vitamin A stores, and cell motility (Fig. 8E–G), paralleling the phenotype observed after HCV cure.

Using the same gating strategy, we identified within the reverted population a lipid-high, PTK2-low subset (~20%), termed C2.1(m) (Fig. S9F and G). This subset exhibited higher vitamin A content (Fig. 8H), lower collagen expression (Fig. S9H), and elevated transcripts of genes involved in vitamin A metabolism and homeostatic/hepatoprotective functions (Fig. S9I), consistent with a more quiescent state, analogous to that observed after HCV cure.

Finally, blocking IL-10 signaling—either by *IL10*RB downregulation in HSCs or with an IL-10–neutralizing antibody—impaired restoration of vitamin A in the MASLD reversal paradigm, indicating that IL-10 similarly supports reversion in this context (Fig. 8I).

Discussion

Our study establishes that hiPSC-derived HSCs activated by either HCV infection or MASLD-like lipotoxic stress can undergo reversion when the injurious stimulus is removed. By integrating functional assays with scRNA-seq, we demonstrate that rHSCs exhibit a hybrid phenotype—partially restoring quiescent-like features while retaining distinct characteristics that differentiate them from naïve qHSCs. Importantly, we demonstrate that macrophage-derived IL-10 is a key regulator of this process, acting through transcriptional programs that enhance vitamin A metabolism and suppress fibrogenic activation.

By extending prior rodent studies[8,9] to a human-relevant system, we highlight both the parallels and unique features of HSC plasticity. Similar to mouse models, rHSCs exhibit a 'primed' phenotype with incomplete transcriptional reversion and heightened sensitivity to re-stimulation. Our single-cell analysis further reveals that reversion produces a heterogeneous landscape, spanning cells with quiescent, apoptotic, or senescent signatures. Notably, a distinct subset (lipid-high, PTK2-low), ranging from 10% to 20%, more closely resembles naïve qHSCs, suggesting that a fraction of rHSCs may achieve higher restoration. Identifying and enriching this population could be key to therapeutic strategies that drive fibrosis regression more effectively.

The incorporation of both HCV and MASLD into our model underscores the generalizability of HSC reversion across etiologies. Withdrawal of lipotoxic stress led to phenotypic and functional reversion highly analogous to that observed after HCV cure. These results reinforce that HSC plasticity is not limited to viral injury, but extends to metabolic liver disease. Moreover, the observation that IL-10 promotes reversion in both contexts emphasizes its centrality as a mediator of fibrosis regression.

Despite these restorative tendencies, rHSCs remain transcriptionally and functionally distinct from naïve qHSCs. Elevated transcript levels of activation markers and incomplete restoration of certain quiescence-related factors indicate that reversion does not fully erase the molecular imprint of prior activation. Functionally, rHSCs exhibit intermediate levels of ROS, proliferation, and migration compared to quiescent and activated states, reflecting a poised phenotype.

A striking feature of rHSCs is their heightened sensitivity to re-stimulation. This enhanced responsiveness mirrors observations in CCl_4-induced fibrosis mouse models,[8,9] suggesting that prior activation likely leaves a lasting epigenetic or

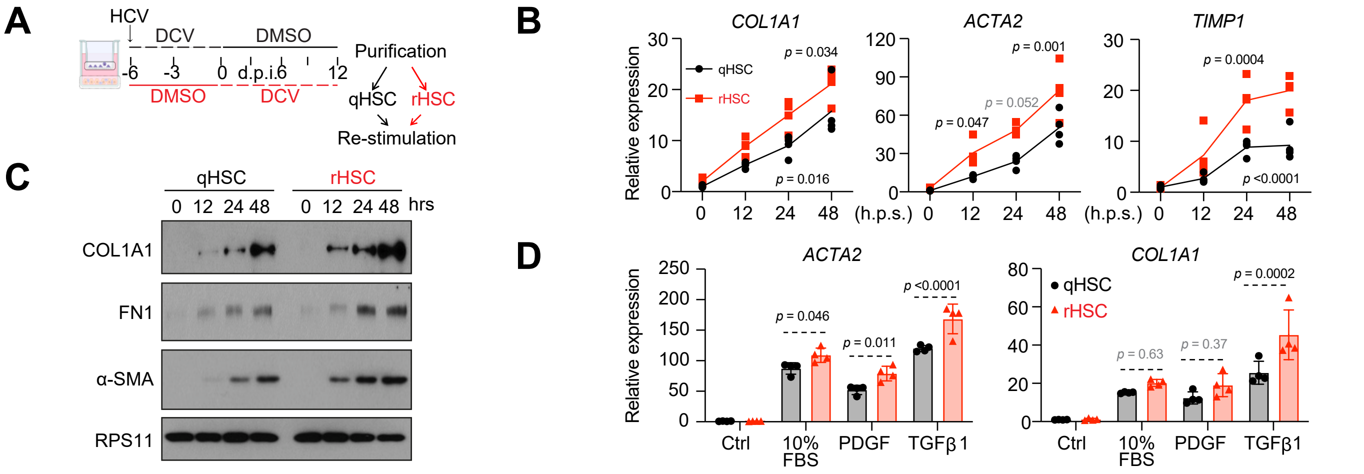

Fig. 6. rHSCs are more sensitive to re-stimulation. (A) A schematic overview of HCV infection, DCV treatment, cell purification, and re-stimulation. Transcript levels of selected activation markers by qRT-PCR (B, n = 4, mean ± SD, ANOVA) or for the selected activation marker and housekeeping protein RPS11 by Western blot (C). (D) Transcript levels of activation markers ACTA2 and COL1A1 at 48 h post 10% FBS, PDGF-BB, or TGFβ1 treatment (n = 4, mean ± SD, ANOVA). α-SMA/ACTA2, alpha-smooth muscle actin; COL1A1/COL1A2/COL6A3, collagen type I alpha 1 chain/alpha 2 chain/collagen type VI alpha 3; DCV, daclatasvir; FN1, fibronectin; PDGFB, platelet derived growth factor subunit B; qRT-PCR, quantitative real time-PCR; rHSCs, reverted hepatic stellate cells; TGFβ1, transforming growth factor beta 1.

transcriptional memory in HSCs. This finding has significant clinical implications: while fibrosis may regress following the removal of causative agents, the liver may remain predisposed to rapid fibrogenesis upon re-exposure to injury. This

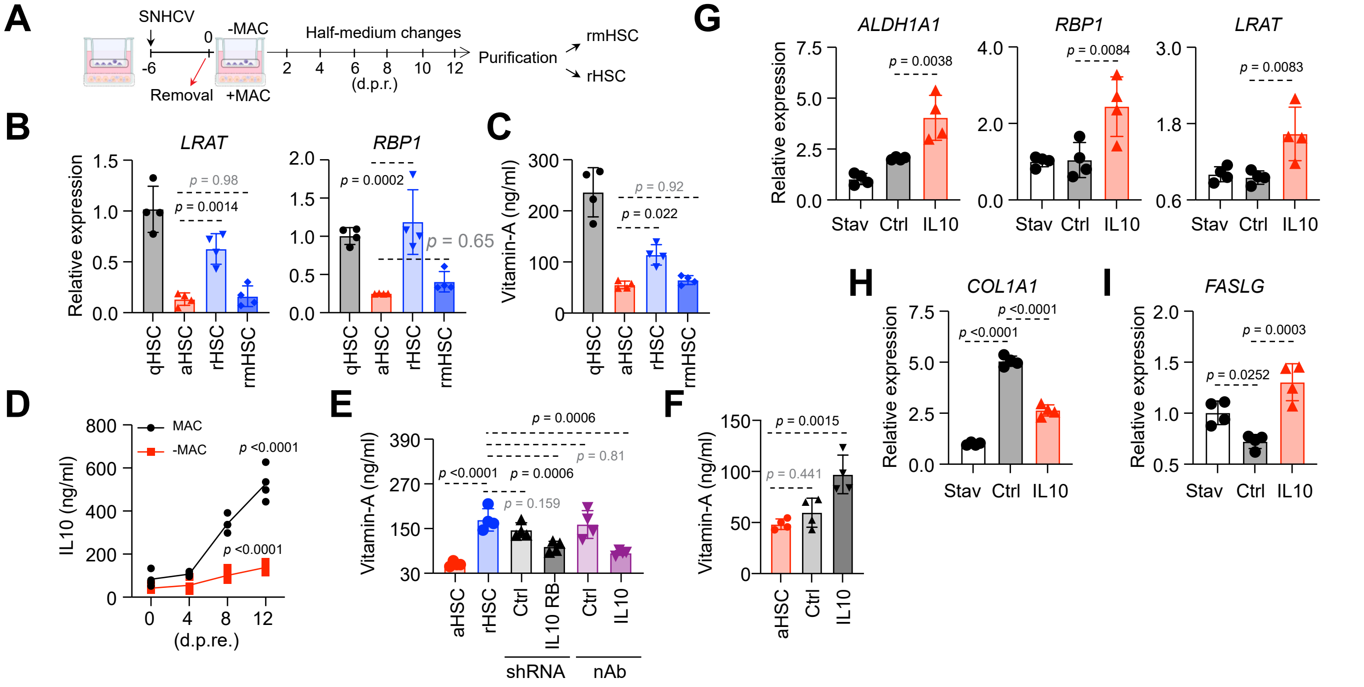

Fig. 7. Critical roles of macrophages in HSC reversion. (A) A schematic of supernatant treatment, macrophage removal, cell purification, and re-stimulation. (B) Transcript levels of *LRAT* and *RBP1* in indicated cell groups by qRT-PCR (n = 4, mean ± SD, ANOVA). (C) ELISA analysis of intracellular vitamin A levels in indicated cell groups (n = 4, mean ± SD, ANOVA). (D) ELISA analysis of IL-10 secretion post macrophage removal (n = 4, mean ± SD, ANOVA). (E) ELISA analysis of the intracellular vitamin A levels in HSCs (n = 4, mean ± SD, ANOVA) purified after the following treatments: HCV-infected liver cultures with 10 nM BMS-790052, followed by switch of basal medium, shRNA transduction, and re-seeding in previous liver culture with IL-10 blocking antibody at medium switch. (F) ELISA analysis of vitamin A levels in HSCs (n = 4, mean ± SD, ANOVA) purified after the following treatments: HCV-infected liver culture with 10 nM BMS-790052, followed by BSA or 40 ng/ml IL-10. (G-I) qHSCs were activated by culturing in 10% FBS, then serum starved before re-exposure to 10% FBS, with BSA or IL-10. At 48 h, cells were analyzed for transcript levels of genes associated with vitamin A metabolism and storage (G), activation markers (H), and apoptotic markers (I) by qRT-PCR (n = 4, mean ± SD, ANOVA). aHSC, activated hepatic stellate cells; ALDH1A1, aldehyde dehydrogenase 1 family member a1; COL1A1, collagen type I alpha 1 chain; FASLG, fas ligand; HSCs, hepatic stellate cells; LRAT, lecithin-retinol acyltransferase; qHSCs, quiescent hepatic stellate cells; qRT-PCR, quantitative real time-PCR; RBP1, retinol-binding protein 1; rHSCs, reverted hepatic stellate cells; rmHSC, reverted hepatic stellate cells from MASLD; shRNA, short hairpin RNA.

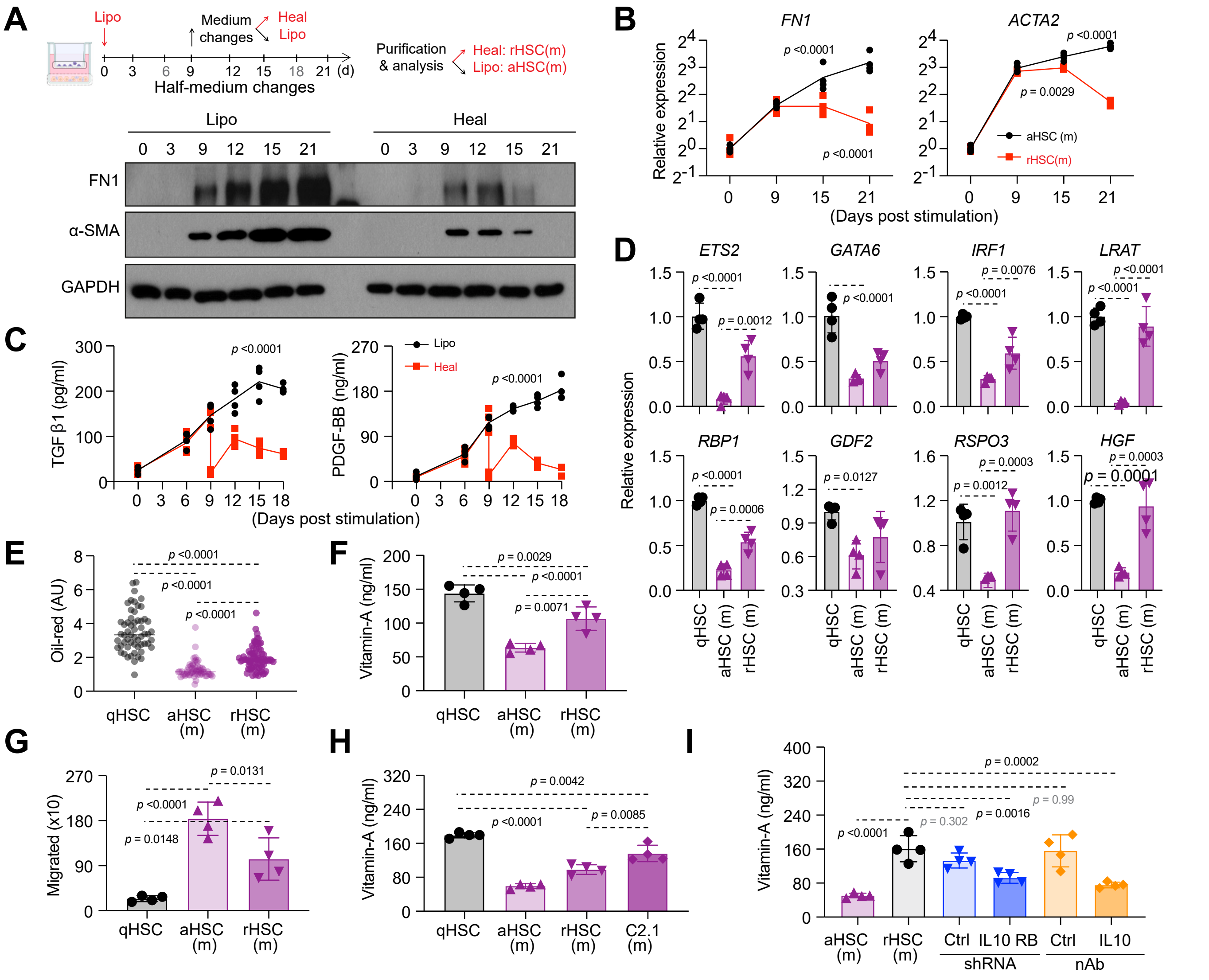

Fig. 8. Reversion of MASLD-activated HSCs. (A) Top: Schematic of treatment and medium changes. For controls, aHSCs(m) were from lipotoxic milieu cultures; qHSCs from healthy milieu cultures. Bottom: HSCs were collected for Western blot analysis. (B) Analysis of the HSC activation marker transcripts by qRT-PCR (mean ± SD, n = 4). (C) ELISA analysis of selected cytokines (n = 4, mean ± SD, ANOVA). (D) Transcript levels of selected quiescence-related genes in qHSC, aHSC(m), and rmHSC by qRT-PCR (n = 4, mean ± SD, ANOVA). (E–G) Oil-red signals (E), quantification of vitamin A (F), and PDGF-BB-induced cell migration assay (G) of purified qHSC, aHSC(m), and rHSC(m) (n = 4, mean ± SD, ANOVA). (H) Quantification of intracellular vitamin A (n = 4, mean ± SD, ANOVA). (I) Quantification of intracellular vitamin A in HSCs (n = 4, mean ± SD, ANOVA) purified after the following treatments: lipotoxic milieu-treated liver cultures, followed by healthy milieu, shRNA-IL10 transduction, and re-seeding in previous liver culture with IL-10 neutralization at medium switch (n = 4, mean ± SD, ANOVA). aHSCs, activated hepatic stellate cells; α-SMA/ACTA2, alpha-smooth muscle actin; ETS2, ETS proto-oncogene 2; FN1, fibronectin; GAPDH, glyceraldehyde 3-phosphate dehydrogenase; GATA6, GATA binding protein 6; GDF2/BMP9, growth differentiation factor 2; HGF, hepatocyte growth factor; HSCs, hepatic stellate cells; IRF1, interferon regulatory factor 1; LRAT, lecithin-retinol acyltransferase; MASLD, metabolic dysfunction-associated steatotic liver disease; PDGFB, platelet derived growth factor subunit B; qHSCs, quiescent hepatic stellate cells; qRT-PCR, quantitative real time-PCR; RBP1, retinol-binding protein 1; rmHSC, reverted hepatic stellate cells from MASLD; RSPO3, R-spondin 3; TGFβ1, transforming growth factor beta 1.

vulnerability underscores the need for strategies that not only promote reversion, but also mitigate this primed state to prevent fibrosis recurrence.

The roles of human macrophages in facilitating HSC reversion emerged as a pivotal finding. Depletion of macrophages impaired the restoration of quiescent markers and vitamin A levels, while IL-10—a key cytokine secreted by M2-polarized macrophages—directly promoted reversion. These results build on rodent studies implicating Kupffer cells in fibrosis resolution[21] and provide a mechanistic link in humans: IL-10 signaling enhances HSC plasticity, tipping the balance toward deactivation. This macrophage-HSC crosstalk highlights the multicellular nature of fibrosis resolution and suggests that therapeutic modulation of macrophage polarization

or IL-10 delivery could amplify reversion, offering a novel avenue for antifibrotic interventions.

Although our hiPSC-derived liver culture system recapitulates key aspects of human liver biology,[10] it has several limitations. It lacks the full complexity of the *in-vivo* microenvironment, such as endothelial cells, immune cells beyond macrophages, and systemic influences.[27] Using HCV and MASLD as stimuli captures important disease aspects but does not cover all fibrogenic causes, such as alcohol, HBV, drugs/toxins. The heterogeneity of rHSCs, although a strength of our single-cell approach, also complicates the identification of uniform therapeutic targets. Lastly, the short culture duration raises the possibility that incomplete reversion of rHSCs may reflect insufficient recovery time. Still, by 12 days post-HCV cure, at the protein level, the expression of key activation markers returns to levels comparable to those observed in naïve qHSCs, suggesting a substantial degree of reversion within this timeframe.

Looking forward, our findings open several research avenues. Elucidating the epigenetic mechanisms underlying the heightened sensitivity of rHSCs to re-stimulation could reveal targets to 'reset' their primed state. Further exploration of C2.1's quiescent-like properties—potentially through lineage tracing or advanced sorting techniques—may clarify whether these cells represent a stable endpoint of reversion or a transient state. In addition, modulating the expression of key transcription factors—particularly those not fully restored in rHSCs (*e.g.* GATA binding protein 4 [*GATA4*], peroxisome proliferator-activated receptor gamma [*PPARG*])—may offer strategies to promote a more complete return to quiescence. Finally, validating the IL-10-mediated reversion pathway in patient-derived samples or *in-vivo* models could accelerate its translation into clinical strategies.

In conclusion, this study demonstrates that activated human HSCs process significant reversion capacity across multiple etiologies, driven in part by macrophage-derived IL-10, yet they remain distinct from naïve quiescent HSCs due to their heterogeneity and sensitivity to re-stimulation. These insights advance our understanding of HSC plasticity and suggest that strategies enhancing reversion while overcoming residual priming may pave the way for durable fibrosis resolution in patients.

Affiliations

[1]*Department of Infection Biology, Lerner Research Institute, Cleveland Clinic, Cleveland, OH, USA;* [2]*Department of Genetics and Genome Sciences, School of Medicine, Case Western Reserve University, Cleveland, OH, USA;* [3]*Department of Pathology School of Medicine, Case Western Reserve University and University Hospitals Cleveland Medical Center, Cleveland, OH, USA;* [4]*Cleveland Clinic College of Medicine at Case Western Reserve University, Cleveland, OH, USA*

Abbreviations

α-SMA/ACTA2, alpha-smooth muscle actin; aHSCs, activated hepatic stellate cells; ALD, alcohol-associated liver disease; ALDH1A1, aldehyde dehydrogenase 1 family member a1; ASGR1, asialoglycoprotein receptor 1; BMM, basal maintenance medium; CCl_4, carbon tetrachloride; CITI, Cleveland Clinic Center for Immunotherapy and Precision Immuno-Oncology; COL1A1/COL1A2/COL6A3, collagen type I alpha 1 chain/alpha 2 chain/collagen type VI alpha 3; CYP26A1, cytochrome p450 family 26 subfamily a member 1; DCV, daclatasvir; ECM, extracellular matrix; EGF, epidermal growth factor; ETS1/ETS2, ETS proto-oncogene 1/ETS proto-oncogene 2; FASLG, fas ligand; FN1, fibronectin; GAPDH, glyceraldehyde 3-phosphate dehydrogenase; GATA4, GATA binding protein 4; GATA6, GATA binding protein 6; GDF2/BMP9, growth differentiation factor 2; GO, Gene Ontology; HGF, hepatocyte growth factor; hiPSC, human induced pluripotent stem cells; HPC, high performance computing; HSCs, hepatic stellate cells; IL10RB, interleukin 10 receptor beta; IRF1/IRF2, interferon regulatory factor 1/2; KOSR, knockout serum replacement; LOX, lysyl oxidase; LRAT, lecithin-retinol acyltransferase; MASLD, metabolic dysfunction-associated steatotic liver disease; MDA, malondialdehyde; MET, MET proto-oncogene, receptor tyrosine kinase; NOTCH, Notch receptor 1; NS5A, nonstructural protein 5A; PDGFB, platelet derived growth factor subunit B; pHSCs, patient quiescent HSCs; PLIN2, perilipin 2; PPARG, peroxisome proliferator-activated receptor gamma; PTK2, protein tyrosine kinase 2; qHSCs, quiescent hepatic stellate cells; RBP1, retinol-binding protein 1; Rest DCV, remaining cells in the DCV group; rHSCs, reverted hepatic stellate cells; rmHSC, reverted hepatic stellate cells from MASLD; ROS: reactive oxygen species; RSPO3, R-spondin 3; SASP, senescence-associated secretory phenotype; scRNA-seq, single-cell RNA sequencing; shRNA, short hairpin RNA; SPARCL1, SPARC like 1; TGFβ1, transforming growth factor beta 1; TNFα: tumor necrosis factor-alpha; *t*-SNE, *t*-distributed Stochastic Neighbor Embedding; Umap, Uniform Manifold Approximation and Projection.

Financial support

XJW, EHH, LB, ZYF, FZ, KOS, LW, AH, YFZ, WBC, and XFW were supported by National Institutes of Health grants R00-AI141742 (XFW), DP2-AI170515 (XFW), R01-DK125100 (subcontract to XFW), and R01- AA031226 (XFW), and by Seed Fund (XFW) provided by the Cleveland Clinic Foundation. YJZ was supported by the Department of Pathology School of Medicine, Case Western Reserve University and University Hospitals Cleveland Medical Center. The funders had no role in study design, data collection and analysis, decision to publish, or preparation of the manuscript.

Conflicts of interest

The authors declare that they have no conflict of interest.
Please refer to the accompanying ICMJE disclosure forms for further details.

Authors' contributions

Study concept and design, XJW, EHH, XFW. Methodology, XJW, EHH, LB, YJZ, XFW. Analysis and interpretation of data, XJW, EHH, XFW. Acquisition of data, XJW, EHH, LB, FZ, ZYF, AH, YFZ, LW, WBC, XFW. Drafting of the manuscript, XJW, EHH, XFW. Critical revision of manuscript, XJW, EHH, LB, FZ, KOS, ZYF, LW, AH, WBC, YJZ. Funding acquisition, XFW. Supervision, XFW.

Data availability

The single-cell RNA sequencing data have been deposited in the Gene Expression Omnibus (GEO) under accession GSE304675. All analysis code—including R scripts, shell (sh) scripts, and HPC-associated workflows—have been released on GitHub at the time of publication. Upon publication, interactive plots and analyses will also be made publicly available on BioTuring (Talk2Data).

Acknowledgements

We thank C. Rice (Rockefeller University) for the Jc1 HCV infectious clone, Huh-7.5 cells, and HCV NS5A (9E10) antibody, and Cleveland Clinic CITI Department for the assistance with the Cleveland Clinic's internal High Performance Computing (HPC) platform and scRNA-seq data analysis.

Supplementary data

Supplementary data to this article can be found online at https://doi.org/10.1016/j.jhepr.2025.101669.

References

Author names in bold designate shared co-first authorship

[1] Tsuchida T, Friedman SL. Mechanisms of hepatic stellate cell activation. Nat Rev Gastroenterol Hepatol 2017;14:397–411.

[2] Chang TT, Liaw YF, Wu SS, et al. Long-term entecavir therapy results in the reversal of fibrosis/cirrhosis and continued histological improvement in patients with chronic hepatitis B. Hepatology 2010;52:886–893.
[3] Poynard T, McHutchison J, Manns M, et al. Impact of pegylated interferon alfa-2b and ribavirin on liver fibrosis in patients with chronic hepatitis C. Gastroenterology 2002;122:1303–1313.
[4] Dixon JB, Bhathal PS, Hughes NR, et al. Nonalcoholic fatty liver disease: improvement in liver histological analysis with weight loss. Hepatology 2004;39:1647–1654.
[5] Iredale JP, Benyon RC, Pickering J, et al. Mechanisms of spontaneous resolution of rat liver fibrosis. Hepatic stellate cell apoptosis and reduced hepatic expression of metalloproteinase inhibitors. J Clin Invest 1998;102:538–549.
[6] **Oh Y**, **Park O**, **Swierczewska M**, et al. Systemic PEGylated TRAIL treatment ameliorates liver cirrhosis in rats by eliminating activated hepatic stellate cells. Hepatology 2016;64:209–223.
[7] Krizhanovsky V, Yon M, Dickins RA, et al. Senescence of activated stellate cells limits liver fibrosis. Cell 2008;134:657–667.
[8] **Troeger JS**, **Mederacke I**, Gwak GY, et al. Deactivation of hepatic stellate cells during liver fibrosis resolution in mice. Gastroenterology 2012;143:1073. 83.e1022.
[9] Kisseleva T, Cong M, Paik Y, et al. Myofibroblasts revert to an inactive phenotype during regression of liver fibrosis. Proc Natl Acad Sci U S A 2012;109:9448–9453.
[10] **Park J**, **Zhao Y**, **Zhang F**, et al. IL-6/STAT3 axis dictates the PNPLA3-mediated susceptibility to non-alcoholic fatty liver disease. J Hepatol 2023;78:45–56.
[11] McCaughan GW, George J. Fibrosis progression in chronic hepatitis C virus infection. Gut 2004;53:318–321.
[12] **Zhang F**, **Zhao Y**, **Bian L**, et al. IL1β signaling mediates the interaction between hepatitis B and C viruses. Hepatology 2025. https://doi.org/10.1097/HEP.0000000000001525.
[13] Liu X, Xu J, Rosenthal S, et al. Identification of lineage-specific transcription factors that prevent activation of hepatic stellate cells and promote fibrosis resolution. Gastroenterology 2020;158:1728. 44.e1714.
[14] **Sugimoto A**, **Saito Y**, **Wang G**, et al. Hepatic stellate cells control liver zonation, size and functions via R-spondin 3. Nature 2025;640:752–761.
[15] Filliol A, Saito Y, Nair A, et al. Opposing roles of hepatic stellate cell subpopulations in hepatocarcinogenesis. Nature 2022;610:356–365.
[16] **Trinh VQ**, **Lee TF**, Lemoinne S, et al. Hepatic stellate cells maintain liver homeostasis through paracrine neurotrophin-3 signaling that induces hepatocyte proliferation. Sci Signal 2023;16. eadf6696.
[17] **Zhao D**, **Huang Z**, **Li X**, et al. GDF2 and BMP10 coordinate liver cellular crosstalk to maintain liver health. eLife 2024;13. RP95811.
[18] Schwabe RF, Brenner DA. Hepatic stellate cells: balancing homeostasis, hepatoprotection and fibrogenesis in health and disease. Nat Rev Gastroenterol Hepatol 2025;22:481–499.
[19] **Payen VL**, **Lavergne A**, Alevra Sarika N, et al. Single-cell RNA sequencing of human liver reveals hepatic stellate cell heterogeneity. JHEP Rep 2021;3:100278.
[20] D'Ambrosio DN, Clugston RD, Blaner WS. Vitamin A metabolism: an update. Nutrients 2011;3:63–103.
[21] Duffield JS, Forbes SJ, Constandinou CM, et al. Selective depletion of macrophages reveals distinct, opposing roles during liver injury and repair. J Clin Invest 2005;115:56–65.
[22] Tacke F, Zimmermann HW. Macrophage heterogeneity in liver injury and fibrosis. J Hepatol 2014;60:1090–1096.
[23] Wan J, Benkdane M, Teixeira-Clerc F, et al. M2 Kupffer cells promote M1 Kupffer cell apoptosis: a protective mechanism against alcoholic and nonalcoholic fatty liver disease. Hepatology 2014;59:130–142.
[24] Boldanova T, Suslov A, Heim MH, et al. Transcriptional response to hepatitis C virus infection and interferon-alpha treatment in the human liver. EMBO Mol Med 2017;9:816–834.
[25] Govaere O, Cockell S, Tiniakos D, et al. Transcriptomic profiling across the nonalcoholic fatty liver disease spectrum reveals gene signatures for steatohepatitis and fibrosis. Sci Transl Med 2020;12. eaba4448.
[26] **Massey V**, **Parrish A**, **Argemi J**, et al. Integrated multiomics reveals glucose use reprogramming and identifies a novel hexokinase in alcoholic hepatitis. Gastroenterology 2021;160:1725. 40.e1722.
[27] Starokozhko V, Groothuis GMM. Challenges on the road to a multicellular bioartificial liver. J Tissue Eng Regen Med 2018;12:e227–e236.

Keywords: Hepatic stellate cells (HSC); HSC reversion; Single-cell RNA sequencing; Heterogeneity; Re-stimulation; HSC-macrophage interaction; IL-10 signaling; Multicellular liver culture; Disease modeling.
Received 2 April 2025; received in revised form 23 October 2025; accepted 30 October 2025; Available online 10 November 2025

Journal of Hepatology, Volume *8*

Supplemental information

Single-cell analysis of heterogeneity in reverted hiPSC-derived human hepatic stellate cells

Xinjia Wang, Eun Hee Ha, Lu Bian, Zhuoying Feng, Fan Zhang, Kyle O'Shaughnessy, Lei Wang, Andrea Hochwald, Yifei Zheng, Weibo Chen, Yujie Zhang, and Xianfang Wu

Single-cell analysis of heterogeneity in reverted hiPSC-derived human hepatic stellate cells

Xinjia Wang, Eun Hee Ha, Lu Bian, Zhuoying Feng, Fan Zhang, Kyle O'Shaughnessy, Lei Wang, Andrea Hochwald, Yifei Zheng, Weibo Chen, Yujie Zhang, and Xianfang Wu

Table of contents

Supplementary materials and methods 2

Fig. S1 9

Fig. S2 11

Fig. S3 12

Fig. S4 13

Fig. S5 14

Fig. S6 15

Fig. S7 17

Fig. S8 18

Fig. S9 20

Supplementary materials and methods

Stem cell maintenance and differentiation.

Human induced pluripotent stem cells (hiPSCs) including iPSC-9 and iPSC-W3 were cultured on growth factor-reduced matrigel according to the manufacturer's recommendations in a feeder-independent mTeSR1-based medium (Stemcell Technologies, Canada). Cultures were replenished with fresh medium daily. Cells were passaged every 4 - 6 days as clumps using ReLeSR. For all the experiments in this study, hiPSCs were used between passages 30 and 40.

The two iPSC lines iPSC-9 and iPSC-W3 were generated from de-identified fibroblast cells purchased from a commercial supplier (ATCC) and their use was reviewed and approved by Cleveland Clinic Institutional Biosafety Committee (IBC 2101). Key experimental findings obtained from both iPSCs are comparable. Data shown in this manuscript are from iPSC-W3 line.

Establishment of a multicellular coculture.

hiPSC-derived hepatocytes, HSCs, and macrophages were differentiated separately and assembled into a coculture as described previously [10]. Cocultures were maintained in a basal maintenance medium (BMM) that is consisted of glucose-free DMEM supplemented with 4% knockout serum replacement (KOSR), 2% B-27, 3U/ml heparin, 200μg/ml Transferrin, 30ng/ml EGF, 5μM retinol, and 0.5μM dexamethasone. The 12-well plate containing the cocultures was placed on an orbital shaker platform at a speed of 30 rpm/min. The coculture was replenished with fresh medium every two days, or as indicated in the figure legend.

Separation of hepatocytes from hepatic stellate cells.

Hepatocytes were isolated from cocultures as described previously [10]. Briefly, hiPSC-derived co-cultures of hepatocytes and HSCs were washed once with Versene and then

incubated in Versene at 37°C for 20–25 minutes to loosen cell–cell contacts. Cultures were subsequently washed with pre-warmed DMEM/F12 and incubated with a pre-warmed collagenase mixture (2.0 mg/ml collagenase, 1.0 mg/ml dispase, 100 U/ml DNase, 0.2% DMSO in HBM) at 37°C for 35–40 minutes. During this incubation, cells were gently pipetted to disrupt clumps and facilitate dissociation. Following digestion, cells were collected by adding Versene and centrifuged at 400 g for 5 minutes at room temperature. The pellet was resuspended in Versene and incubated again at 37°C for 45 minutes. By the end of this incubation, the majority of cells were dissociated into single cells. If necessary, the suspension was passed through a 100 μm cell strainer to remove residual clumps.

To separate hepatocytes from HSCs, single-cell suspensions were first blocked in 1% BSA and incubated on ice for 1 hour. Cells were then stained with mouse anti-ASGR1 antibody, followed by incubation with anti-mouse IgG microbeads, and subjected to magnetic separation at 4°C. Hepatocytes were collected from the magnetically bound fraction, while HSCs were obtained from the flow-through after an additional round of hepatocyte depletion. The purified hepatocytes and HSCs were subsequently collected for downstream analysis such as single cell RNA sequencing, immunofluorescent staining, western blot, and RNA extraction.

Lentivector-based transduction.

Lentiviral particles-based shRNAs targeting *IL10RB* gene were cloned into the pLKO.1-puro vector using standard techniques. To generate lentiviral particles, packaging plasmids were transfected into Lenti-293T cells using Lipofectamine 2000 according to the manufacturer’s directions, and the medium was changed to DMEM supplement with 3% ESC-qualified FBS (Life Technologies) at 6 hr post-transfection. The medium containing the lentiviral particles was harvested at 24 and 48 hrs post transfection and

filtered through a 0.2 μm filter. Subsequently, lentiviral particles were further concentrated (100:1) using Lenti-X Concentrator according to the manufacturer's directions.

To transduce hiPSC-derived HSCs, cells were exposed twice to a mixture of lentivectors and maintenance medium (1:10 ratio) in the presence of polybrene (4μg/ml) and a JAK inhibitor (Pyridone 6, 100nM), with each exposure lasting 4 hrs. Following transduction, thorough washing with pre-warmed DMEM/F12 was performed, and the cells were maintained in complete coculture maintenance medium until the experiments ended.

***Immunofluorescence analysis*.**

Cells were fixed in 4% para-formaldehyde in phosphate-buffered saline (PBS) at room temperature for 10 min and blocked with PBTG (PBS containing 10% normal goat serum, 1% bovine serum albumin (BSA), 0.1% Triton-X100) at room temperature for 2 to 3 hrs. Cells were incubated with primary antibodies (diluted in PBTG) at 4°C overnight or 2 hr at room temperature. Isotype mouse or rabbit IgGs were used as negative controls. After four washes with PBS, Alexa-594 conjugated secondary antibodies (1:1000 diluted in PBTG) were added and incubated in the dark at room temperature for 1 hr, followed by four washes with PBS.

To stain lipid droplets in hiPSC-derived HSCs, purified cells were cultured on a matrigel-coated plate in the respective medium for 6 to 8 hrs. Subsequently, the cells were fixed with 10% formalin for 30 minutes, followed by a 10-minute wash with 60% isopropanol at room temperature. Cells were then stained with 0.18% freshly prepared oil-red in PBS for 4 min at room temperature and washed thoroughly with double-distilled water four times. Nuclei were stained with DAPI for 1 min at room temperature. Images were captured using an Olympus IX73 Inverted Fluorescence Microscope System (Tokyo, Japan).

To quantify lipid content at the single-cell level, we analyzed fluorescence microscopy images in which lipid droplets and nuclei were co-stained and captured in a single channel. Using ImageJ software, each image was first converted to grayscale, and nuclei were segmented using Otsu thresholding followed by morphological filtering and watershed-based separation to identify individual cells. We then measured the lipid signal intensity within each segmented nucleus, extracting key parameters including nuclear area, mean lipid intensity, and the range of lipid signal (maximum and minimum intensity) for downstream analysis. Primary antibodies used for Immunofluorescence analysis are listed in **Supplementary CTAT Table**.

Quantitative real-time RT-PCR (RT-qPCR).

Total RNA was isolated from cell lysates using the RNAeasy Mini Kit (Qiagen, Germany) or PureLink RNA Mini Kit (Invitrogen) followed by reverse transcription using RevertAid First Strand cDNA Synthesis (Thermo Fisher). Gene expression was quantified using the AzuraView GreenFast qPCR Blue Mix (Azura Genomics) on a LightCycler 480 Instrument (Roche Life Science, Germany) or a QuantStudio3 Instrument (Applied Biosystems) with gene-specific primers shown in **Supplementary CTAT Table**.

PCR conditions were as follows: initial denaturation step at 50°C for 2 min and 95°C for 10 min, then 45 cycles of 95°C for 15 sec, 56°C for 15 sec, and 72°C for 20 sec; followed by a melting step of 95°C for 10s, 65°C for 10s and a 0.07°C/s decrease from 95°C; and finally, a cooling step of 50°C for 5s. A melting-curve analysis confirmed PCR product specificity. The fold changes in mRNA expression were determined using the ΔΔCt method relative to the values in control samples as indicated in figure legends, after normalization to housekeeping genes (RPS11 or GAPDH). Unless stated otherwise, results are presented as means ± standard deviation (SD). Comparisons between groups/cells were made using the two-tailed t-test with Welch's corrections or One-way

ANOVA/Tukey's post-hoc test to calculate exact p-values, unless stated otherwise. Statistical analysis was performed in Graph Pad PRISM 10.

Western blot analysis.

Cells were directly lysed in 2x SDS lysis buffer (2mL Tris-HCl (pH 6.8), 50% glycerol, 10% SDS, 0.5% bromophenol blue, and freshly added 10% β-mercaptoethanol) and cell lysates were separated by 7.5%, 10%, or 12% sodium dodecyl sulfate-polyacrylamide gel electrophoresis, in MES or MOPS buffer, followed by transfer onto polyvinylidene fluoride (PVDF) membrane (EMD Millipore). GAPDH or RPS11 were used as housekeeping protein controls for comparison between different groups or treatments, as indicated in the figures and figures legends. Primary antibodies used for western blot analysis are listed in **Supplementary CTAT Table**.

Hepatitis C Virus (HCV) stock.

The HCV stock was prepared through a sequential process. Initially, the wildtype (WT) Jc1 HCVcc was generated by harvesting supernatant containing viral particles at 72 hours post electroporation of Huh-7.5 cells with *in vitro* transcribed HCV RNA. Subsequently, the WT stock was employed to infect naïve Huh-7.5 cells and subjected to serial passaging, surpassing 40 passages to establish the SAV HCVcc stock. All virus stocks were aliquoted and stored at -80°C. For infection, liver cultures were exposed to HCVcc at the indicated MOIs for 6 hrs. Following this, a thorough washing with DMEM/F12 was conducted, and the cells were then transitioned to a maintenance medium.

Quantification of HCV infection.

To quantify intracellular HCV RNA copies, RNA extraction from infected cells was carried out using the RNeasy kit (Qiagen, Germany) or PureLink RNA Mini Kit (Invitrogen), followed by cDNA synthesis using the RevertAid first strand cDNA synthesis kit (Thermo

Scientific). Relative quantification of HCV RNA copies was performed by qPCR using a specific primer pair targeting HCV NS3. The expression levels were normalized to uninfected cells, and the results were presented as fold changes.

ELISAs.

To measure the secreted levels of human cytokines, including pro-inflammatory cytokines (IFNβ1, IL6, TNFα, and IL1β), profibrotic cytokines (TGFβ1 and PDGF-BB), and HGF in the liver cultures under different experiment settings, supernatant was collected at indicated time points and cytokine levels were quantified by ELISA kits (R&D Systems), according to the manufacturers' instructions. To quantify albumin secretion, liver cultures were washed thoroughly with DMEM/F12 medium three times, followed by an incubation with albumin-free medium for 3 hrs; they were then analyzed by ELISA kits (R&D Systems), according to the manufacturers' instructions. Quantifications of human vitamin A in different HSCs were performed using a human vitamin A ELISA kit (CUSABIO), according to the manufacturer' instructions.

Collagen quantification.

Intracellular collagen levels in different HSCs were quantified by a colorimetric hydroxyproline assay kit (Millipore Sigma), according to the manufacturer's instructions.

Cell proliferation*.*

The proliferation of different HSCs was assessed using a BrdU cell proliferation assay kit (Cell Signaling Technologies), according to the manufacturer's instructions.

Cellular reactive oxygen species (ROS)*.*

The levels of ROS in different HSCs were assessed using a DCFA/H2DCFDA kit and detected by a VarioSkan LUX Multi-Mode *Plate Reader (Thermo Scientific), following the manufacturers' instructions.*

Flow cytometry.

Purified reverted HSCs were stained with BioTracker 488 green lipid droplet dye (Millipore Sigma) for 30 minutes in the dark at 37°C prior to flow cytometry sorting. Based on fluorescence intensity, the cells were sorted into two populations: the top 10% and the remaining 90%. These sorted populations were subsequently subjected to downstream analyses, including qRT-PCR, western blotting, intracellular vitamin A quantification, and collagen measurement.

Hepatic function assays.

Hepatic functional characterizations included the quantification of intracellular glycogen levels (Promega) and extracellular levels of total bile acids (Cell Biolabs). These were performed using the purified hepatocytes from liver cultures, following the manufacturers' instructions.

Culture of primary human HSCs.

pHSCs were purchased from Lonza and cultured according to the manufacturer's instructions using the recommended stellate cell growth medium and supplements. To ensure identity and quality, the purity of pHSCs was assessed by flow cytometry using anti-αSMA staining. Only preparations that displayed high purity were used for experiments. Cells were used between passages 2 and 5 to avoid phenotypic drift and discarded beyond passage 5.

Induction of MASLD-like phenotypes in liver cultures.

Liver cultures are maintained in basal medium supplemented with the normal physiological level of insulin (0.7 nM) and glucose (6 mM) to mimic healthy condition (healthy) or high levels of insulin (7 nM), glucose (25 nM), and free fatty acids (oleic acid 68 μM and palmitic acid, 45 μM) to mimic plasma concentrations of these factors in MASH patients (lipotoxic) for the indicated days.

Fig. S1

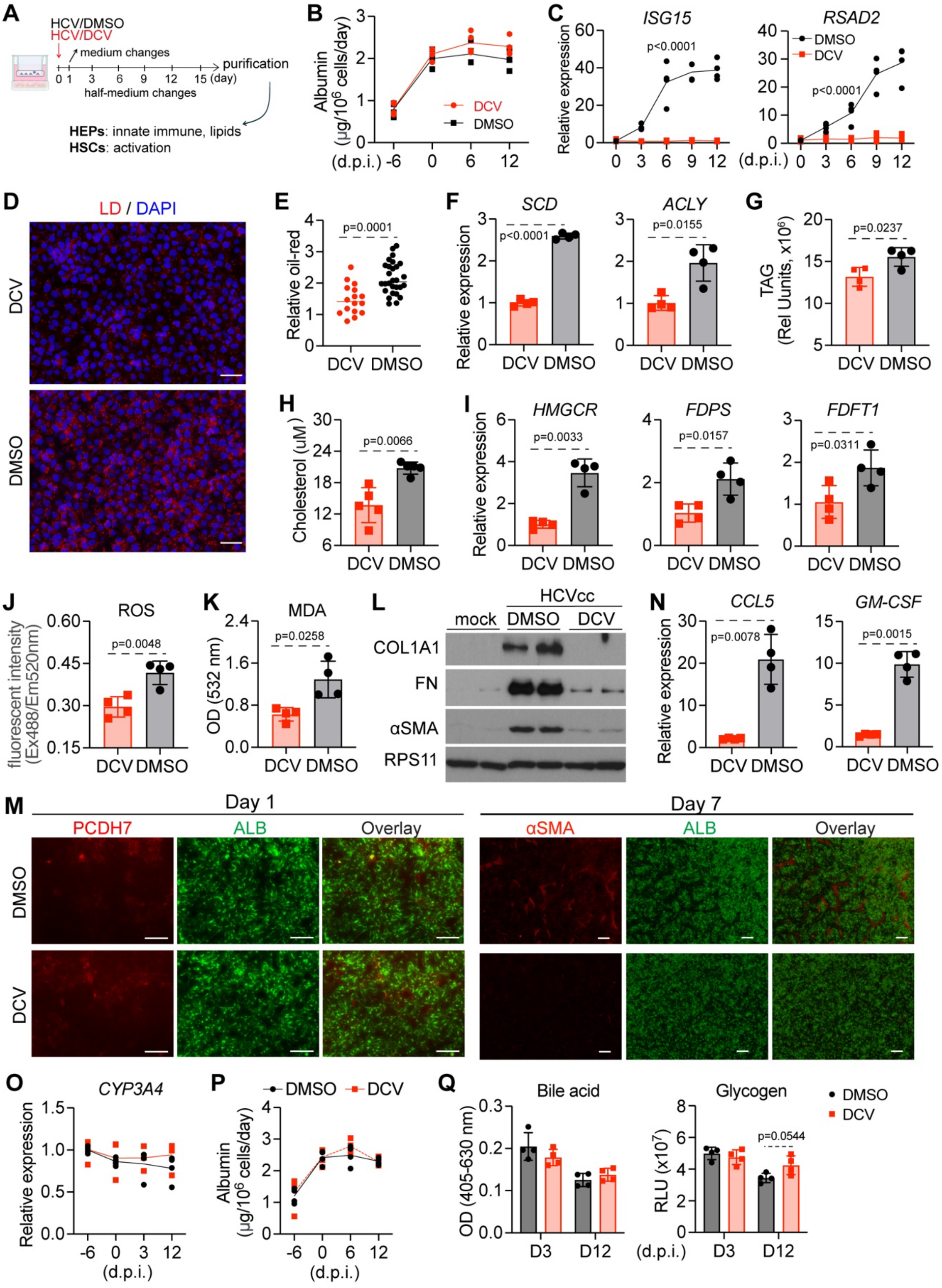

Fig. S1. Establishment of a human-relevant *in vitro* system to study HSC activation and reversion.

(**A**). Schematic representation of HCV infection and medium change. Liver cultures were infected with SAV HCVcc (MOI=1.0) in the presence or absence of DCV (BMS-790052,

10nM) for overnight before the inoculum was removed. At day 15 post infection, Hepatocytes (HEPs) and HSCs were purified for downstream analyses.

(**B**). To quantify albumin secretion at the indicated time points, the culture medium was replaced with albumin-free medium for 3 hrs and then analyzed by ELISA (B). Shown are mean ± SD from n=4 independent experiments.

(**C**). Analysis of transcript levels of ISG15 and RSAD2 in hepatocytes purified at the indicated time points by qRT-PCR. Shown are mean ± SD from n=4 independent experiments.

(**D-E**). At day 15 post infection, hepatocytes were purified and re-seeded onto matrigel-coated plates for 4 hrs before cells were fixed for oil-red staining (D, scale bars, 100μm) and red-signals were quantified using ImageJ software (1.52k) (E).

(**F, I**). At day 15 post infection, hepatocytes were purified for analysis of transcript levels of the selected lipogenesis-related genes (F) or cholesterol-related genes (I) by qRT-PCR. Shown are mean ± SD from n=4 independent experiments.

(**G, H**). At day 15 post infection, hepatocytes were purified for analysis of triacylglycerol (TAG, G) or cholesterol (H) by Triglyceride-Glo and Cholesterol-Glo kit, respectively. Shown are mean ± SD from n=4 independent experiments.

(**J-K**). At day 15 post infection, hepatocytes were purified for analysis of reactive oxygen species (ROS) by DCFH-DA fluorescence assay (Ex488/Em520nm, J), and for malondialdehyde (MDA) by ELISA kit. Shown are mean ± SD from n=4 independent experiments.

(**L, N**). At day 15 post infection, HSCs were purified for western blot analysis of activation markers and housekeeping protein RPS11 (L), and for analysis of transcript levels of the selected cytokine genes by qRT-PCR (N). Shown in (N) are mean ± SD from n=4 independent experiments.

(**M**). At day 1 and 7 post infection, liver cultures (the bottom compartment containing hepatocytes and HSCs) were fixed for immunofluorescent staining of HSC marker PCDH7 and α-SMA (Scale bars, 100μm).

(**O-P**). Analysis of transcript levels of *CYP3A4* in hepatocytes purified from the experiments described in Figure 1A by qRT-PCR (O). To quantify albumin secretion at the indicated time points, the culture medium was replaced with albumin-free medium for 3 hrs and then analyzed by ELISA (P). Shown are mean ± SD from n=4 independent experiments.

(**Q**). In the experiments described in Figure 1A, culture supernatants were collected to quantify total bile acids (C). Purified hepatocytes were used to assess intracellular glycogen levels (B). Shown are mean ± SD from n=4 independent experiments.

Statistical analysis was performed using Unpaired t tests with Welch's correction or One-way ANOVA/Tukey's post-hoc test to calculate exact p-values.

Fig. S2

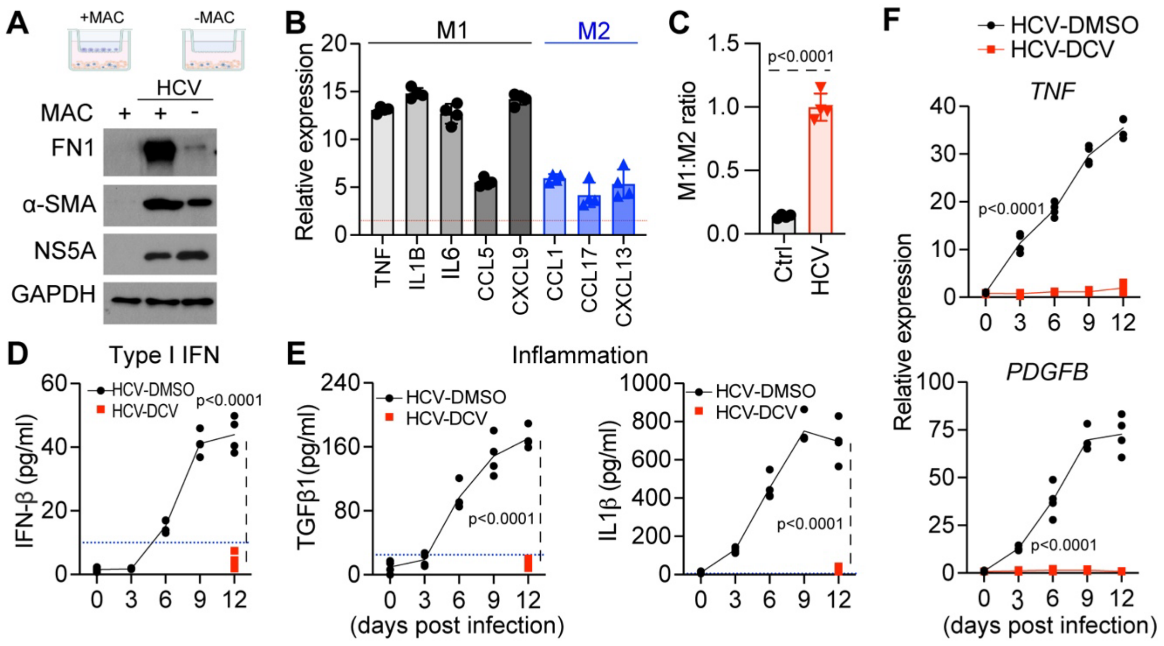

Fig. S2. Critical roles of macrophages to HCV infection-induced HSC activation.

(**A**). Top: Schematic representation of liver cultures with or without macrophages. Bottom: Liver cultures were infected with infected with SAV HCVcc (MOI=1.0) for one week and cells from the bottom compartments were collected for western blot analysis of HSC activation markers, HCV NS5A, and housekeeping protein GAPDH.

(**B**). Liver cultures were infected with SAV HCVcc (MOI=1.0) in the presence or absence of DCV (BMS-790052, 10nM) for 7 days. Macrophages were isolated and subject to qRT-PCR analysis of the selected marker genes that are associated with M1 or M2 macrophages. Shown are mean ± SD normalized to uninfected control from 4 independent experiments.

(**C**). Macrophages from the experiments in (B) were analyzed for cell surface markers CD86 (M1) and CD200R (M2) by flow cytometry. The percentages of CD86 or CD200R positive cells from 4 independent experiments were used to calculate M1:M2 ratio.

(**D-E**). From the experiments in (A), supernatants were collected for analysis of the selected cytokines by ELISA. For cultured with DCV, only samples from the last time points were included. Shown are mean ± SD from n=4 independent experiments.

(F). From the experiments in (A), macrophages were collected at the indicated time points for analysis of transcript levels of *TNF* and *PDGFB* by qRT-PCR. Shown are mean ± SD from n=4 independent experiments.

Statistical analysis was performed using Unpaired t tests with Welch's correction or One-way ANOVA/Tukey's post-hoc test to calculate exact p-values.

Fig. S3

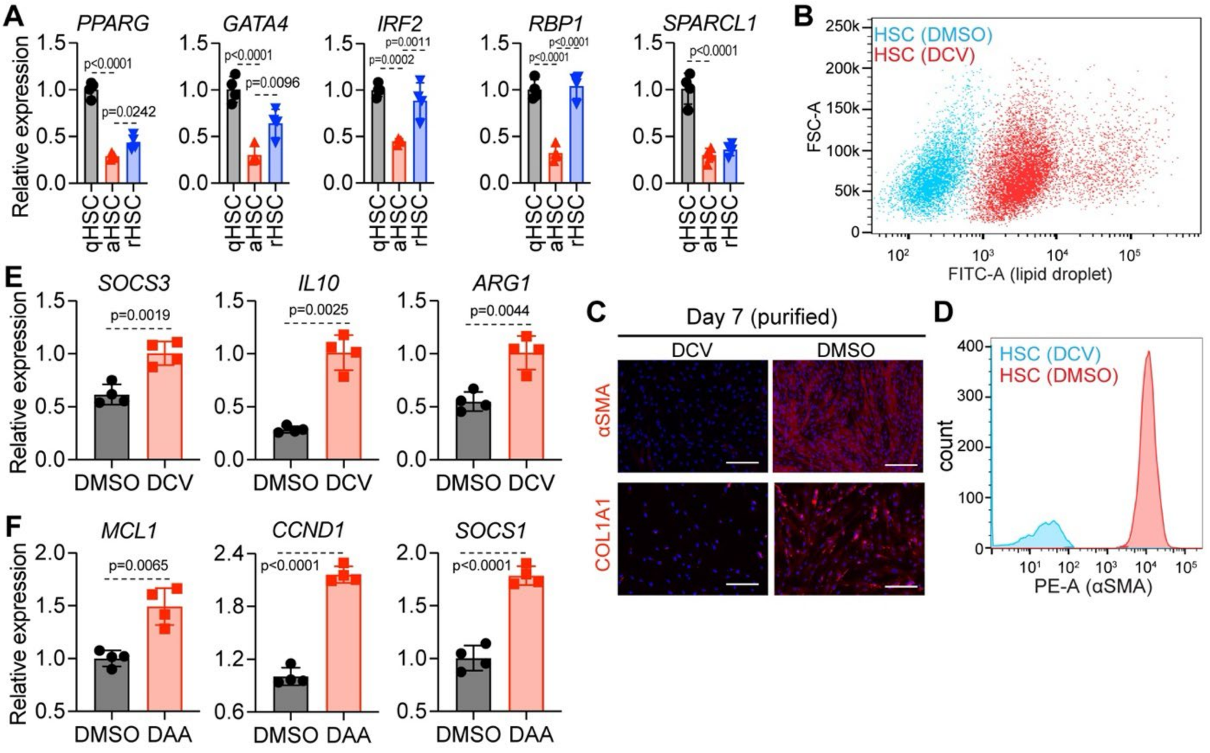

Fig. S3. Detailed characterization of reverted HSCs.

In the experiments described in Figure 1A, quiescent HSCs (qHSCs) were isolated from uninfected liver cultures; activated HSCs (aHSCs) were from infected liver cultures treated with DMSO; and reverted HSCs (rHSCs) were infected liver cultures treated with DCV (10nM). All HSCs were purified from liver cultures at 12 days post-treatment.

(**A**). Analysis of transcripts of the selected quiescence-related transcription factor and homeostatic function genes in qHSC, aHSC, and rHSC by qRT-PCR. Shown are mean ± SD from n=4 independent experiments.

(**B**). Representative flow cytometry analysis of intracellular lipid content in HSCs purified from DMSO (aHSCs) and DCV (rHSCs) treated liver cultures at day 12 post-treatment.

(**C-D**). At day 7 post-HCV exposure, HSCs in liver cultures with DMSO and DCV were purified and re-seeded on matrigel or poly-lysine-coated plates for 6 hrs before being fixed for immunofluorescent staining of HSC activation markers (C); or were subjected to flow cytometry analysis of αSMA (D). Nucleus were stained with DAPI (Scale bars, 100μm).

(**E-F**). At day 12 post-treatment, hepatocytes (E) and macrophages (F) were collected for analysis of transcript levels of HGF target genes by qRT-PCR. Shown are mean ± SD from n=4 independent experiments.

Statistical analysis was performed using Unpaired t tests with Welch's correction or One-way ANOVA/Tukey's post-hoc test to calculate exact p-values.

Fig. S4

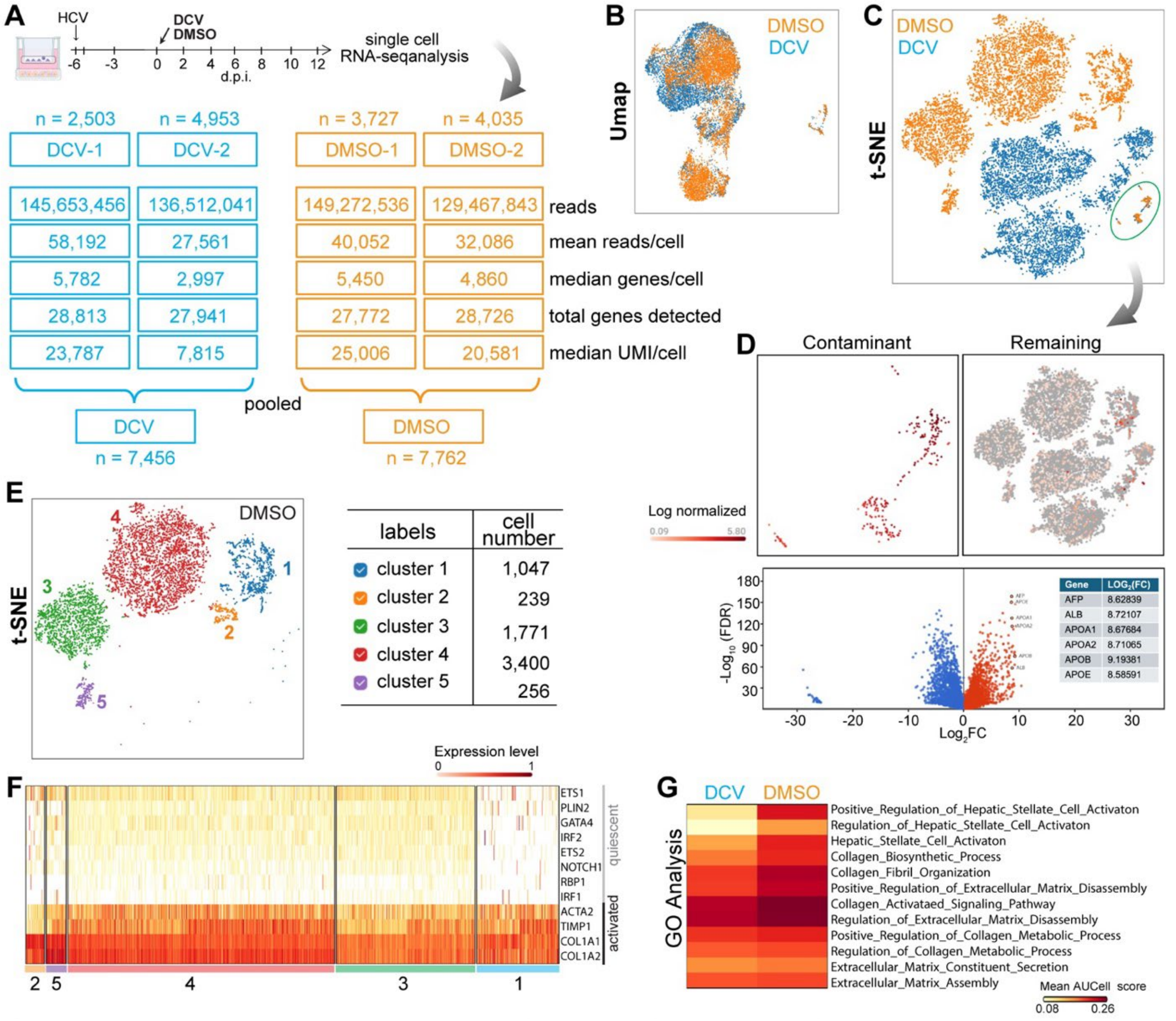

Fig. S4. Single cell RNA-sequencing analysis to compare activated and reverted HSCs.

(**A**). Schematic of the timeline of the experiments, including the date of administering DMSO and DCV, along with the subsequent summary consisting of the detailed parameters used for running single cell RNA sequencing analysis.

(**B**). Umap visualization of DMSO and DCV groups.

(**C**). t-SNE visualization of DMSO and DCV groups with the hepatocyte contamination region outlined in green. (**D**). Differential expression analysis between the contamination region and all other remaining single cell RNA sequencing population.

(**E**). t-SNE visualization of DMSO subclustered into five clusters via Louvain sub-clustering method with 0.1 resolution. Among 6,830 DMSO treated cell population, 1,047 cells, 239 cells, 1,771 cells, 3,400 cells, and 256 cells are subclustered from clusters 1 through 5, respectively.

(**F**). Heatmap depicting the gene expression profiles for activation and quiescent markers for the five DMSO subclusters.

(**G**). Gene ontology analysis of DMSO and DCV groups with the selected GO terms associated with HSC activation, collagen, and extracellular matrix.

Fig. S5

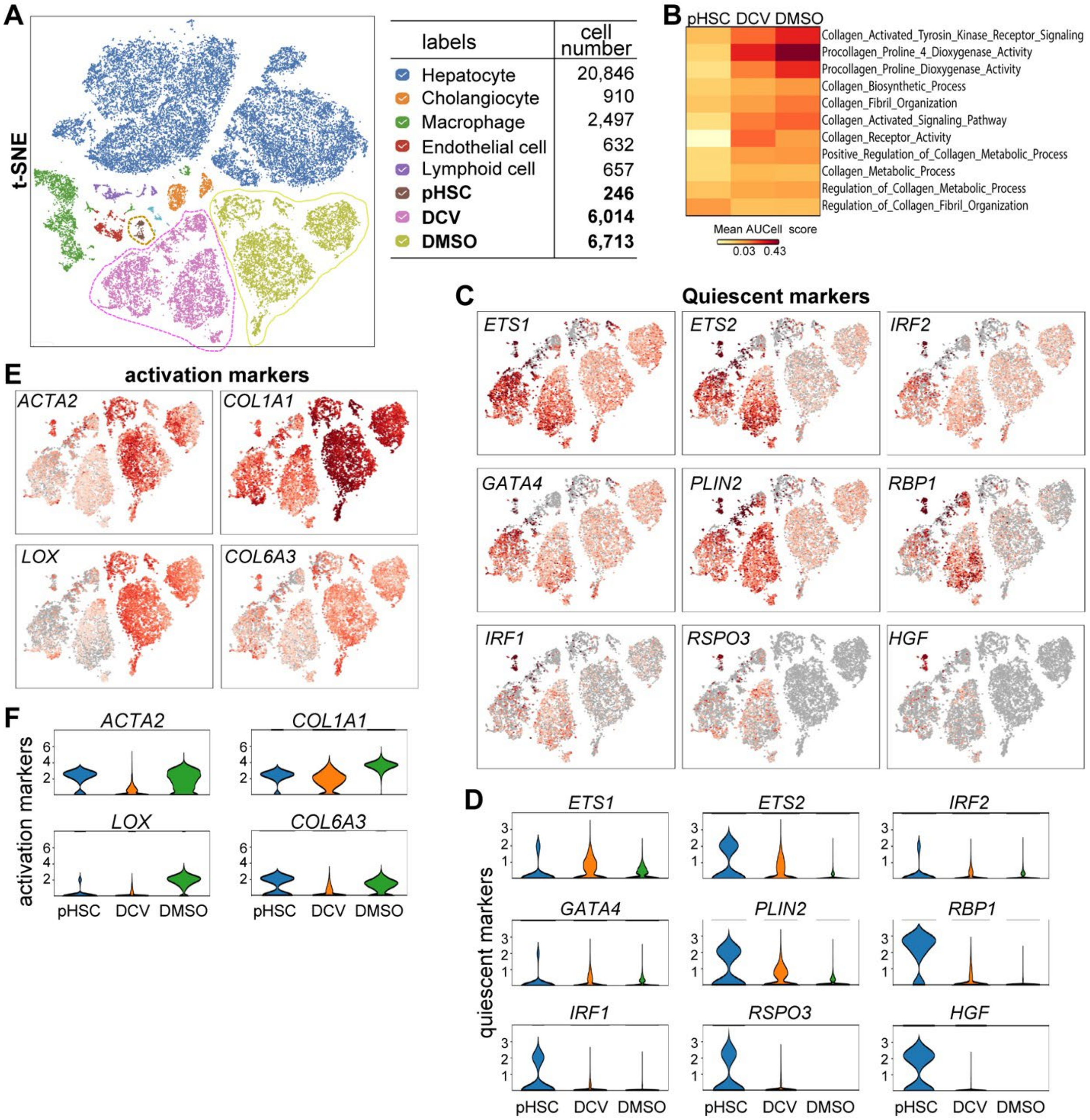

Fig. S5. Comparison between hiPSC-derived reverted HSCs with primary HSCs.

(**A**). t-SNE visualization of hiPSC-derived HSCs in DMSO (aHSCs) and DCV (rHSCs) groups with various human hepatic cells (data from GSE158723) including primary HSCs (pHSCs). Different HSC populations were circled in different colors: aHSCs in yellow-green; rHSCs in pink, and pHSCs in brown.

(**B**). Gene ontology enrichment test of collagen production and regulation genes comparing different HSC populations.

(**C-D**). t-SNE plots (C) and violin plots (D) of individual quiescent-related markers and their relative gene expression between different HSC populations.

(**E-F**). t-SNE plots (E) and violin plots (F) of individual activation markers and their relative gene expression between different HSC populations.

Fig. S6

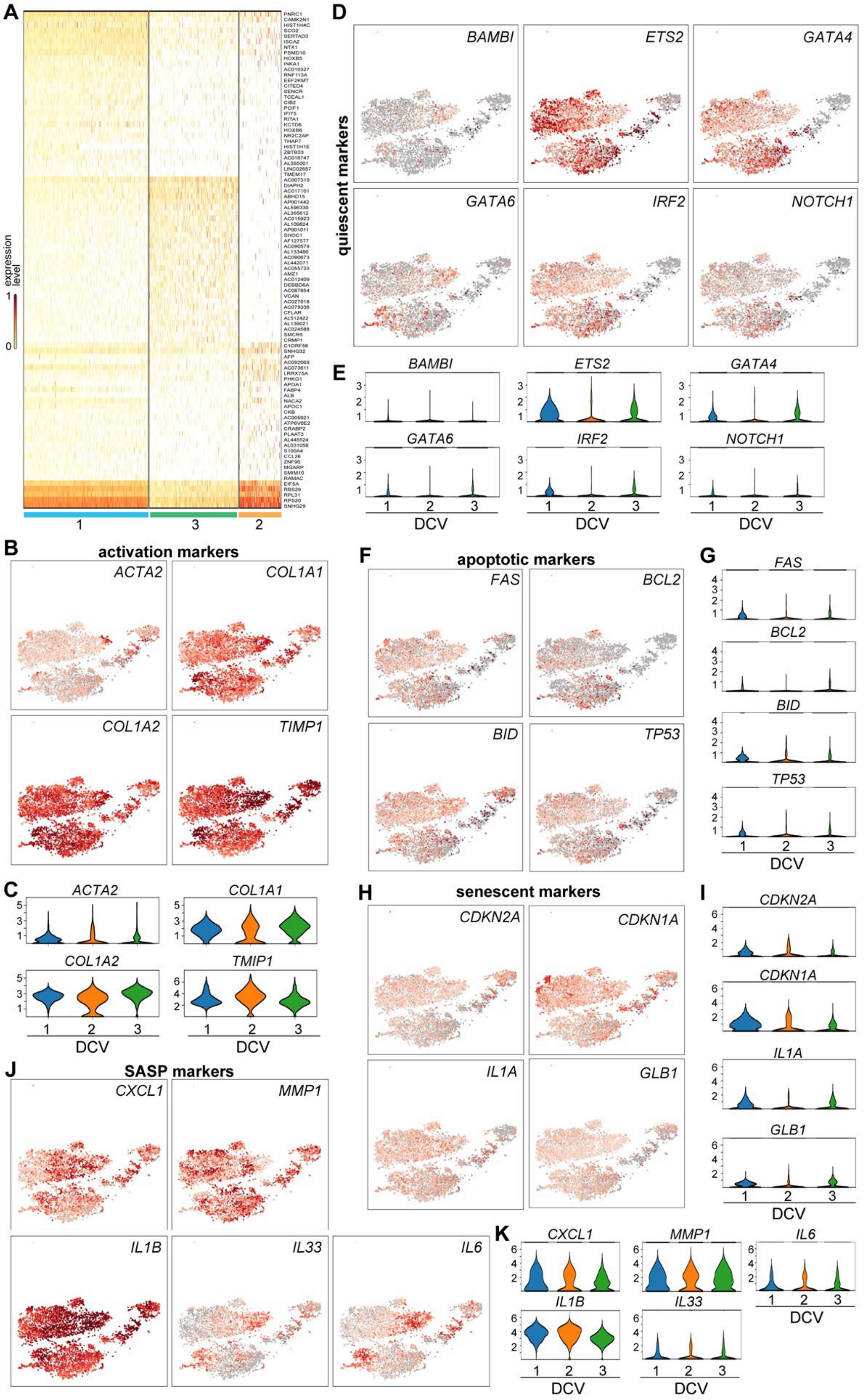

Fig. S6. Single cell RNA-sequencing analysis of reverted HSCs.

(**A**). Heatmap depicting the relative gene expression levels between the three subclusters of the DCV treated group with (p-value<0.05) and (log_2 fold change>0), as well as the number of marker genes selected to maximum value (30 marker genes per group).

(**B-C**). t-SNE plots (B) and violin plots (C) of individual activation markers and their relative gene expression between different clusters within DCV group.

(**D-E**). t-SNE plots (D) and violin plots (E) of individual quiescent-related markers and their relative gene expression between different clusters within DCV group.

(**F-G**). t-SNE plots (F) and violin plots (G) of individual apoptosis-related markers and their relative gene expression between different clusters within DCV group.

(**H-I**). t-SNE plots (H) and violin plots (I) of individual senescence-related markers and their relative gene expression between different clusters within DCV group.

(**J-K**). t-SNE plots (J) and violin plots (K) of individual SASP-related markers and their relative gene expression between different clusters within DCV group.

Fig. S7

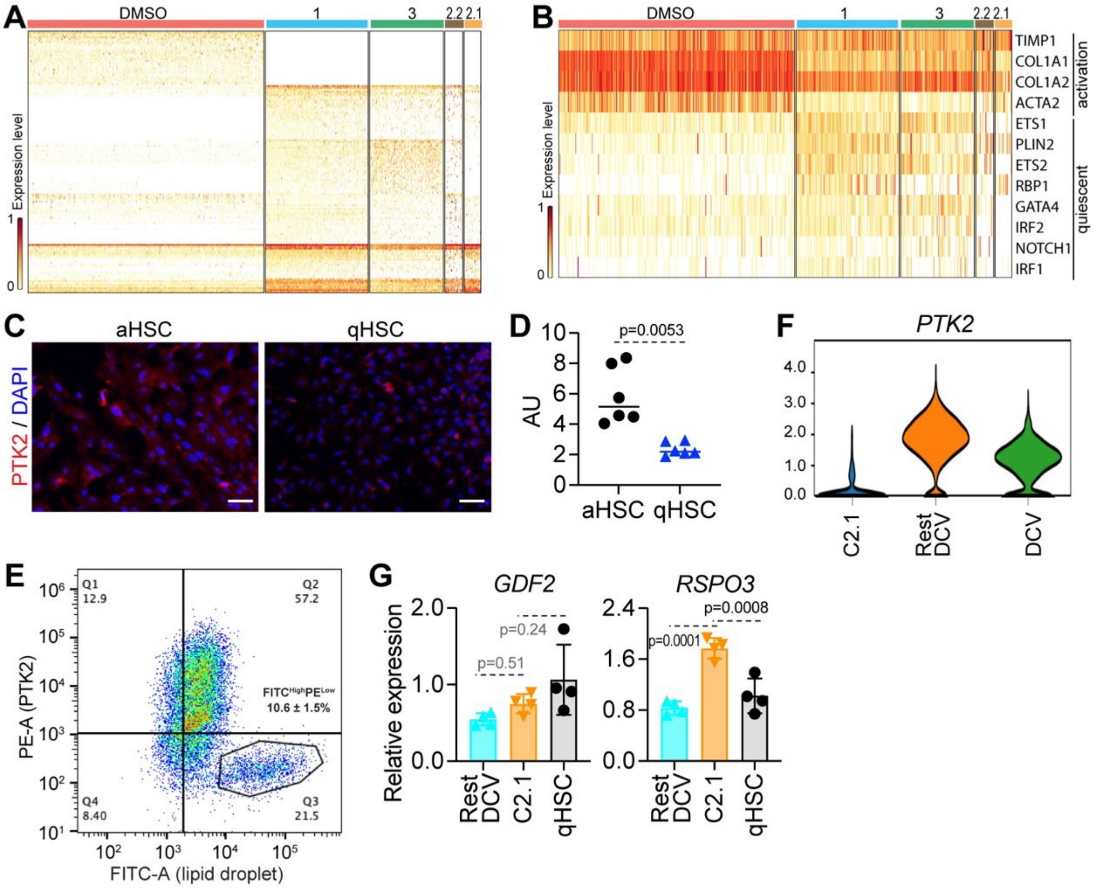

Fig. S7. Identification of a small population within rHSCs resembling quiescent HSCs.

(**A**). Heatmap demonstrating differentially expressed genes between DMSO and the four subclusters of DCV, with (p-value<0.05) and (log_2 fold change>0), and number of marker genes set to maximum per group (30 genes).

(**B**). Heatmap demonstrating the difference in gene expression levels of HSC activation and quiescent markers between DMSO and the four subclusters of DCV.

(**C-D**). Representative immunofluorescent staining of PTK2 in hiPSC-derived activated HSCs (aHSC, activated by culturing in 10% FBS for 4 days) and quiescent HSCs (qHSC) (C, scale bars, 100μm) and red signals were quantified using ImageJ software (1.52k) (D, arbitrary unit/AU).

(**E**). Representative flow cytometry analysis of intracellular lipid content (FITC channel) and PTK2 (PE channel) of reverted HSCs (rHSCs). A subset of lipid-high, PTK2-low cells was selected, with percentage shown from n=3 independent experiments.

(**F**). Violin plots of PTK2 levels in different HSC populations.

(**G**). Analysis of transcript levels of the selected genes related to HSC homeostatic functions in the indicated cell groups by qRT-PCR. Shown are mean ± SD from n=4 independent experiments.

Statistical analysis was performed using Unpaired t tests with Welch's correction or One-way ANOVA/Tukey's post-hoc test to calculate exact p-values.

Fig. S8

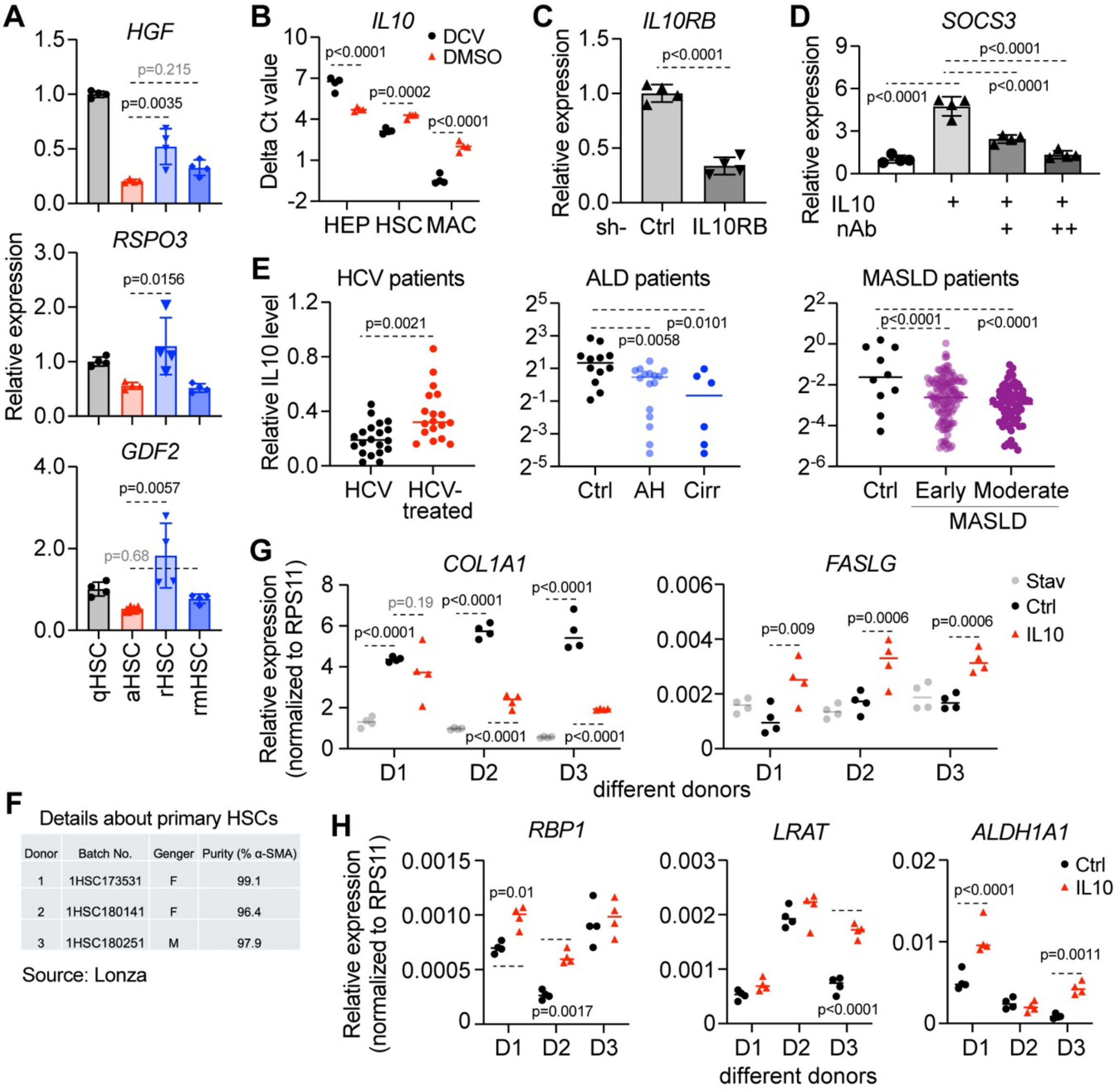

F Details about primary HSCs

Donor	Batch No.	Genger	Purity (% α-SMA)
1	1HSC173531	F	99.1
2	1HSC180141	F	96.4
3	1HSC180251	M	97.9

Source: Lonza

Fig. S8. Critical roles of macrophages in HSC reversion.

(**A**). In the experiments shown in Figure 7A, transcript levels of genes related to HSC homeostatic functions including *HGF*, *RSPO3*, and *GDF2* in the indicated cell groups were analyzed by qRT-PCR. Shown are mean ± SD from n=4 independent experiments.

(**B**). In the experiments described Figure S1A, at day 15 post-viral exposure, individual cell types were purified to analyze transcript levels of *IL-10*. Shown are mean ± SD of delta Ct from n=4 independent experiments.

(**C**). hiPSC-derived activated HSCs were transduced with lentivirus-based control shRNA or shRNA against *IL10RB* for 48 hrs before cells were harvested for analysis of *IL10RB* transcript. Shown are mean ± SD from n=4 independent experiments.

(**D**). hiPSC-derived hepatocytes were treated with either BSA control, or IL-10 (20ng/ml) in the presence of increasing concentration of IL-10 neutralizing antibody (0, 100, 300ng/ml) for 24 hrs before cells were collected for analysis of *SOCS3* transcript. Shown are mean ± SD from n=4 independent experiments.

(**E**). Analysis of transcript levels of IL-10 in liver biopsy samples from HCV patients with and without treatment (GSE84346), from MASLD patients (GSE135251), and from ALD patients (GSE142530).

(**F-H**). Activated pHSCs from three different donors (F) were serum starved (stav) for 24 hours before being re-exposed to 10% FBS, in the presence of BSA control or IL10 (40ng/ml). At 48 hours post re-exposure, the cells were collected to analyze the transcript levels of genes associated with activation marker and apoptotic marker (G), and of genes associated with vitamin A metabolism and storage (H), by qRT-PCR. Shown are mean ± SD of expression normalized to RPS11, from n=4 independent experiments.

Statistical analysis was performed using Unpaired t tests with Welch's correction or One-way ANOVA/Tukey's post-hoc test to calculate exact p-values.

Fig. S9

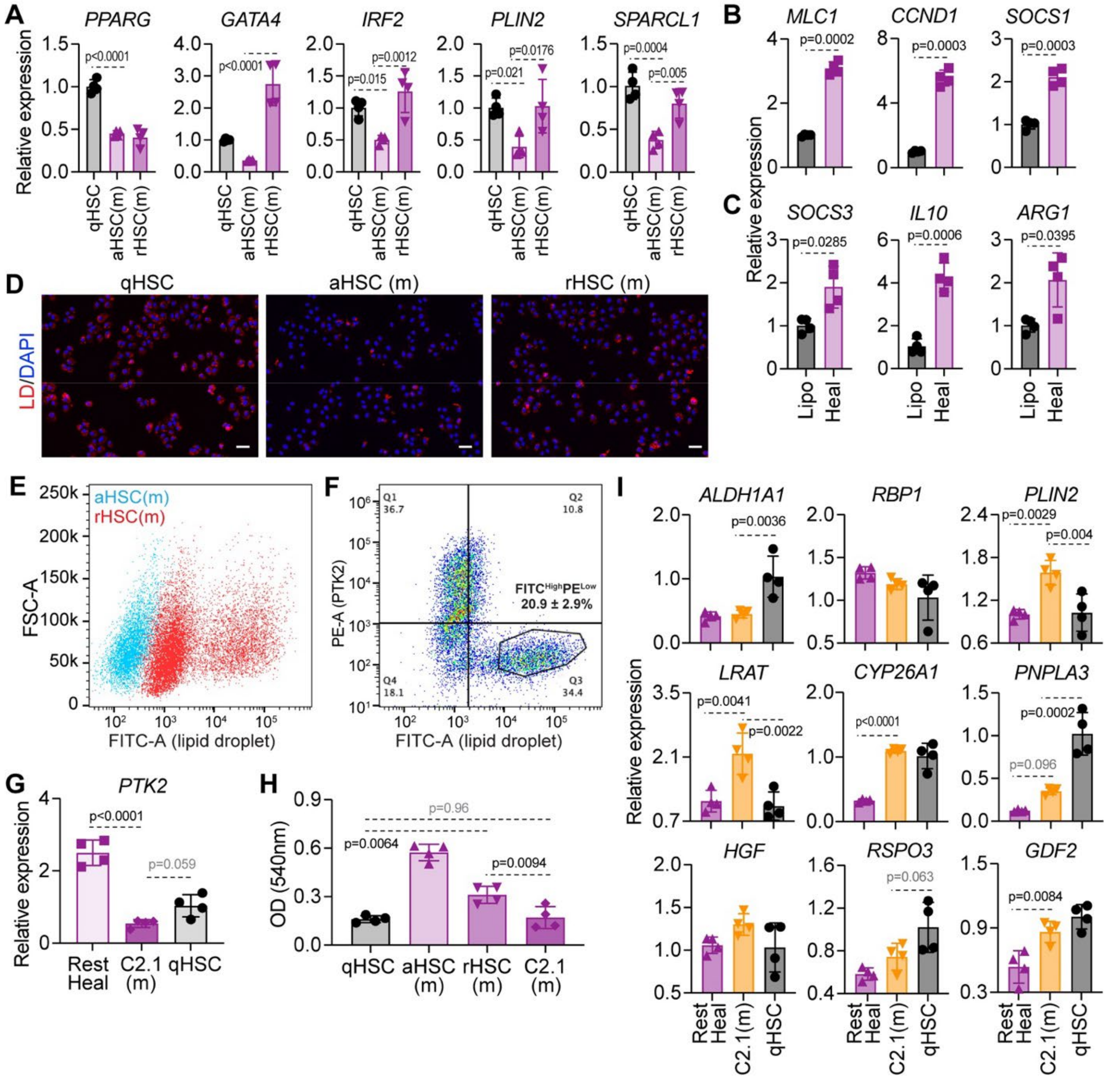

Fig. S9. Reversion of MASLD-activated HSCs.

(**A**). In the experiments described in Figure 8A, transcripts of the selected quiescence-related transcription factor and homeostatic function genes in qHSC, aHSC(m), and rHSC(m) were analyzed by qRT-PCR. Shown are mean ± SD from n=4 independent experiments.

(**B-C**). At day 12 post-medium switch, hepatocytes (B) and macrophages (C) were collected for analysis of transcript levels of HGF target genes by qRT-PCR. Shown are mean ± SD from n=4 independent experiments.

(**D**). Purified qHSC, aHSC(m), and rHSC(m) were seeded onto a matrigel-coated plates in the medium from which they were harvested for 6 hrs before cells were fixed for staining of lipid droplets by oil-red.

(**E**). Representative flow cytometry analysis of intracellular lipid content in HSCs purified from lipotoxic (aHSCs(m)) and healthy (rHSCs(m)) treated liver cultures at day 12 post-treatment.

(**F**). Representative flow cytometry analysis of intracellular lipid content (FITC channel) and PTK2 (PE channel) of rHSCs(m). A subset of lipid-high, PTK2-low cells was selected, with percentage shown from n=3 independent experiments.

(**G**). Expression of *PTK2* in the indicated cell groups was analyzed by qRT-PCR. Shown are mean ± SD from n=4 independent experiments.

(**H**). Analysis of collagen levels in the indicated cell groups by the hydroxyproline assay. Shown are mean ± SD from n=4 independent experiments.

(**I**). Analysis of transcript levels of the selected genes related to vitamin A metabolism and storage, as well as HSC homeostatic functions in the indicated cell groups by qRT-PCR. Shown are mean ± SD from n=4 independent experiments.

Statistical analysis was performed using Unpaired t tests with Welch's correction or One-way ANOVA/Tukey's post-hoc test to calculate exact p-values.
